# Anti-Inflammatory Activity of a Cyclic Tetrapeptide in Mouse and Human Experimental Models

**DOI:** 10.3390/pharmaceutics12111030

**Published:** 2020-10-28

**Authors:** Michał Zimecki, Jolanta Artym, Wojciech Kałas, Leon Strządała, Katarzyna Kaleta-Kuratewicz, Jan Kuryszko, Andrzej Kaszuba, Krzysztof Kaczmarek, Janusz Zabrocki

**Affiliations:** 1 Institute of Immunology and Experimental Therapy, Polish Academy of Sciences, ul. Weigla 12, 53-112 Wrocław, Poland; jolanta.artym@hirszfeld.pl (J.A.); wojciech.kalas@hirszfeld.pl (W.K.); leon.strzadala@hirszfeld.pl (L.S.); 2 Department of Biostructure and Animal Physiology, Wrocław University of Environmental and Life Sciences, ul. C. K. Norwida 25, 50-375 Wrocław, Poland; katarzyna.kaleta-kuratewicz@upwr.edu.pl (K.K.-K.); jan.kuryszko@upwr.edu.pl (J.K.); 3 Department of Dermatology, Pediatric and Oncological Dermatology, Medical University of Łódź, Al. Kościuszki 4, 90-419 Łódź, Poland; andrzej.kaszuba@icloud.com; 4 Institute of Organic Chemistry, Łódź University of Technology, ul. Żeromskiego 116, 90-924 Łódź, Poland; krzysztof.kaczmarek@p.lodz.pl (K.K.); janusz.zabrocki@p.lodz.pl (J.Z.)

**Keywords:** cyclic tetrapeptide, tacrolimus, pimecrolimus, oxazolone, toluene diisocyanate, colitis, pleurisy, prostaglandin receptors, PGE2

## Abstract

A cyclic tetrapeptide Pro-Pro-Pheβ^3^ho-Phe (4B8M) was tested for immunosuppressive activity and potential therapeutic utility in several in vitro and in vivo mouse and human models. The tetrapeptide was less toxic for mouse splenocytes in comparison to cyclosporine A (CsA) and a parent cyclolinopeptide (CLA). The tetrapeptide demonstrated potent anti-inflammatory properties in antigen-specific skin inflammatory reactions to oxazolone and toluene diisocyanate as well to nonspecific irritants such as salicylic acid. It also inhibited inflammatory processes in an air pouch induced by carrageenan. In addition, 4B8M proved effective in amelioration of animal models corresponding to human diseases, such as nonspecific colon inflammation induced by dextran sulfate and allergic pleurisy induced by ovalbumin (OVA) in sensitized mice. The tetrapeptide lowered expression of EP1 and EP3 but not EP2 and EP4 prostaglandin E2 (PGE2) receptors on lipopolysaccharide-stimulated Jurkat T cells and ICAM-1 expression on human peripheral blood mononuclear cells (PBMC). Its anti-inflammatory property in the carrageenan reaction was blocked by EP3 and EP4 antagonists. In addition, 4B8M induced an intracellular level of PGE2 in a human KERTr keratinocyte cell line. In conclusion, 4B8M is a low toxic and effective inhibitor of inflammatory disorders with potential therapeutic use, affecting the metabolism of prostanoid family molecules.

## 1. Introduction

Immunosuppressive drugs, such as cyclosporine A (CsA), tacrolimus, and rapamycin, demonstrate undesirable side-effects upon prolonged application [1]. The search for new drugs devoid of side-effects, particularly peptides from natural sources and their synthetic analogs, represents a challenge for medical chemistry. Isolated in 1959 from linen seeds, a strongly hydrophobic cyclic nonapeptide, cyclolinopeptide A (CLA) [2], was described as a strong immunosuppressor with a potency comparable to that of CsA [3]. The mechanism of action of CLA was proposed to be similar to that of CsA, since CLA was shown to form a complex with cyclophilin A, leading to inactivation of calcineurin [4,5]. CLA suppressed both the humoral and cellular immune response and graft-versus-host reaction; prolonged acceptance of alogeneic skin grafts; ameliorated the symptoms of post-adjuvant polyarthritis in rats and hemolytic anemia in New Zealand black mice; and, as in the case of CsA, diminished IL-1 and IL-2 production [6]. However, high hydrophobicity of CLA was one of the obstacles for potential application of the compound in therapy. Synthesis of new analogs of CLA aimed to improve their bioaccessibility and activity led to the following conclusions. Linear CLA analogs, bearing alanine residue in successive positions of the peptide chain, also demonstrated suppressive activities [6]. Of interest, the activity of linear CLA analogs gradually decreased with shortening of the peptide chain from the N-end and, at the same time, displaying an increase of activity for C-terminal tetra- and tripeptides [7]. The introduction of a single, hydrophilic threonine residue into the CLA molecule did not result in improved solubility in water. However, an improvement in solubility could be achieved by introducing a sulfonic group in the “*para*” position of the aromatic ring of one or two phenyloalanine residues without loss of biological activity [8,9]. In addition, Pro-Pro-Phe-Phe or Pro-Phe-Phe CLA fragments seemed to be responsible for the immunosuppressive activity [10,11]. Further studies aimed to confirm the role of the *cis*-peptide bond between proline residues. Therefore, we synthesized a series of analogs where this bond was replaced with a 1,5-disubstituted tetrazole ring as a proper mimetic of amide bonds in the *cis* configuration. The obtained peptides exhibited strong immunosuppressive activities. It could be also concluded that the Pro-Pro-Phe-Phe fragment was crucial for the biological activity of CLA [12].

In order to improve solubility of CLA in aqueous milieu, we performed syntheses of analogs where leucine residues in position 5 and/or 8 were replaced with its hydroxymethyl analog. The obtained peptides, despite diminution of the biological activity by 25% as compared with native CLA, were characterized by a better (four-fold) solubility in water [13]. A replacement of proline residues by ß^2^-isoproline and ß^3^-homoproline [14] allowed us to obtain a series of nine CLA analogs of particularly strong inhibitory properties, comparable to CsA, in the cellular immune response. The majority of these analogs were practically devoid of cell toxicity.

The aim of this study was to present, for the first time, an evaluation of the immunosuppressive activities of a modified cyclic tetrapeptide Pro-Pro-Phe-β^3^hoPhe (denoted as 4B8M) using mouse and human cells in in vitro models as well as in vivo models of the immune response in mice. Appropriate reference compounds or commercially available therapeutics were used in the experimental models. In addition, we attempted to establish a plausible mechanism of action using molecular techniques, cytofluorometric assays, and in vivo models. Due to the enormous volume of the experimental data, we present only a short overview of the results in the main body of text, whereas preliminary in vitro data, a description of additional experiments, and most of the histological documentation are contained in the Supplementary Materials.

## 2. Materials and Methods

### 2.1. Mice

CBA, BALB/c, and C57Bl/6 mice of both sexes, 8–12 weeks old, weighing 19–22 g, delivered by the Breeding Centre of Laboratory Animals at the Institute of Occupational Medicine, Łódź, Poland, were used for the study. Mice were housed in a cage at 21–22 °C with a 12/12-h light/dark cycle and had free access to commercial laboratory chow and filtered tap water. The local ethics committee at the Institute of Immunology and Experimental Therapy, Wrocław, Poland, approved the study (permissions #41/2008 19 November 2008, 61/2009 18 November 2009, 62/2009 18 November 2009, 22/2010 19 May 2010, 52/2010 17 November 2010, 21/2012 18 April 2012, 10/2013 20 March, 2013, and 4/2014 15 January, 2014.

### 2.2. Reagents

Cyclosporin A (CsA) (Sandimmun, Neoral, Sandoz, Basel, Switzerland) in ampoules, RPMI-1640 medium (Cibi/Life Technologies, Inchinnan, UK), fetal calf serum (FCS), serum-free keratinocyte medium, bovine pituitary extract derived from Gibco Thermo Fisher Scientific (Waltham, MA, USA), glutamate, HEPES, sodium pyruvate, antibiotic and antimycotic solution, cyclolinopeptide (CLA), lipopolysaccharide (LPS) from *Escherichia coli* 0:111, concanavalin A (Con A), phytohemagglutinin A (PHA), ovalbumin (OVA), indomethacin, aspirin (acetylsalicylic acid), MTT (93-[4-dimethylthiazol-2-yl]-2,5-diphenyltetrazolium bromide), oxazolone, salicylic acid, toluene diisocyanate (TDI), recombinant Epidermal Growth Factor (EGF), carrageenan (C-1013, Lot 102 K0871), AH23848 (A8227, Lot 090M4618V) (EP4 receptor antagonist), L-798106 (L4545, Lot 020M4617V) (EP3 receptor antagonist), Giemsa and May-Grünwald reagents, hematoxylin, eosin, and toluidine blue were derived from Sigma-Aldrich (St. Louis, MO, USA); acetone was from Acros Organics, Poznań, Poland; and isotonic solution of sodium chloride buffered with phosphates (PBS) was from the Laboratory of General Chemistry, Institute of Immunology and Eperimental Therapy, Wrocław. Narcotan^®^ (halotane) and Pentasa^®^ (5-aminosalicylic acid (5-ASA)) were from Ferring-Leciva (Jesenice, Czech Republic). Hydrocortisonum^®^ (hydrocortisone acetate) was from Jelfa (Jelenia Góra, Poland), Dexaven^®^ (dexamethasone) was from Polfa-Warszawa (Warszawa, Poland), Protopic^®^ (tacrolimus) 0.1% ointment was from Astellas (Dublin, Ireland), Elidel^®^ (pimecrolimus) 1% creme was from Novartis, and DMSO (dimethylsulfoxide) was from Honeywell-Fluka, Toruń, Poland. Culture plates and flasks were purchased from Corning Incorporated (Tewksbury, MA, USA), and Nunc Lab-Tek II Chamber Slides were purchased from Thermo Scientific (Rockford, IL, USA). Cells were grown at 37 °C in 5% CO_2_ (NuAire, Plymouth, MN, USA). A Trypsin-EDTA (ethylenediaminetetraacetic acid, disodium salt) solution (Institute of Immunology and Experimental Therapy, Wrocław) was used to collect the cells.

The peptide 4B8M was synthesized and delivered by one of us (J.Z.), Łódź University of Technology. Synthesis of the peptide was described in a US patent [15]. The peptide was initially dissolved in DMSO and subsequently in the culture medium at a concentration of 1 mg/mL. The peptide solution was stored at −20 °C until use. For topical administration, the peptide was dissolved by means of 1 h stirring in a 50:50 weight mixture of Vaseline and paraffin (obtained from a drug store). A microscopic evaluation showed no presence of crystals in the mixture containing up to 1% of the peptide.

### 2.3. Cell cultures for Molecular Studies

Jurkat T cells and peripheral blood mononuclear cells (PBMC) cells were cultured in a RPMI-1640 medium supplemented with 5% (Jurkat) or 10% (PBMC) FCS, glutamate, HEPES, sodium pyruvate, and an antibiotic and antimycotic solution. KERTr cells were cultured in a serum-free keratinocyte medium supplemented with 20 µg/mL bovine pituitary extract and 20 ng/mL recombinant EGF. For the cell culture studies, 4B8M was suspended in DMSO for 20 mg/mL. Then, it was diluted slowly in warm RPMI-1640 media supplemented with 10% of FCS and 5% of DMSO to obtain a working stock solution of 4B8M (500 µg/mL).

### 2.4. Cytofluorimetry

For cytofluorimetric analyses, the cells were seeded at densities of 2.5 × 10^5^/well and treated for 24 h. After 24 h, cells were collected and incubated with appropriate antibodies. For Intercellular Adhesion Molecule 1 (ICAM-1) expression fluorescein isothiocyanate (FITC)-conjugated anti-ICAM-1 antibody (Santa Cruz, Dallas, TX, USA; 15.2 clone, 1:25) or FITC-conjugated normal mouse IgG1 (Santa Cruz) for 30 min in room temperature (RT) were used.

For intracellular cyclooxygenase (COX)-1 and COX-2 expression, the Intracellular Flow Cytometry (FCM) System (Santa Cruz) was used according to the supplier’s instructions. Cells were incubated with phycoerythrin (PE)-conjuga–ted anti-COX-1, anti-COX-2 or normal rabbit IgG isotype control antibodies (all Santa Cruz, 1:50, 1 h, 4 °C). Washed cells were collected and analyzed on a FACS Callibur cytofluorimeter (Becton Dickinson Biosciences, Franklin Lakes, NJ, USA) and WinMDI 2.9 software.

### 2.5. Western Blotting

Whole cell lysates were prepared using a cold radioimmunoprecipitation (RIPA) buffer supplemented with a SigmaFAST Protease Inhibitor Cocktail (Sigma-Aldrich, St. Luis, MS, USA). The cell lysates were then sonicated using a Sonopuls HD 2070 ultrasonic homogenizer (Bandelin, Berlin, Germany). Protein concentration was determined by the Pierce bicinchoninic acid assay (BCA) Protein Assay Kit (Thermo Fisher Scientifific, Waltham, MA, USA). Protein lysates were separated by SDS-PAGE using 12% resolving gels and transferred (semi-dry, 1 mA/cm^2^) to a poly(vinylidene fluoride) PVDF membrane (0.45 µm pore size; Merck Millipore, Burlington, MA, USA). Membranes were blocked with 1% casein (Sigma-Aldrich) for 1 h at RT, washed with TBST (20 mM Tris, 150 mM NaCl, 0.1% Tween 20 (BioShop Canada, Burlington, NO, Canada)), and subsequently incubated overnight with a primary antibody at 4 °C. After probing with an horseradish peroxidase (HRP)-conjugated secondary antibody for 1 h at RT, proteins of interest were detected using enhanced chemiluminescence (ECL), Western Blotting Substrate (Pierce, Thermo Fisher Scientifific, Waltham, MA, USA). The following antibodies were used in this study: EP1 goat polyclonal IgG (L-15, Sc-22646, 2 μg/mL), EP2 goat polyclonal IgG (E-13, Sc-22196, 1 μg/mL), EP3 rabbit polyclonal IgG (H-200, Sc-20676, 2 μg/mL), EP4 rabbit polyclonal IgG (H-160 Sc-20677, 2 μg/mL) (all Santa Cruz), and secondary polyclonal goat anti-rabbit immunoglobulins/HRP (Dako, Brookings, SD, USA, P0448, 1:2000) and polyclonal rabbit anti-goat immunoglobulins/HRP (Dako, P0449, 1:1000).

### 2.6. Toxicity Test

Mouse splenocytes at a density of 2 × 10^5^/100 µL/well, resuspended in the culture medium, were cultured for 24 h in a cell culture incubator with the preparations at the indicated concentrations. Cell survival was determined by the MTT colorimetric method [16].

### 2.7. Colorimetric MTT Assay for Cell Growth and Kill

Briefly, 25 µL of MTT (5 mg/mL) stock solution was added per well at the end of cell incubation and the plates were incubated for 3 h in a cell culture incubator. Then, 100 µL of the extraction buffer (20% SDS with 50% DMF [dimethylformamide], pH 4.7) was added. After additional overnight incubation, the optical density (OD) was measured at 550 nm with the reference wavelength of 630 nm in a Dynatech 5000 spectrophotometer. The results are given as a percentage of viable cells as compared with appropriate DMSO or controls.

### 2.8. Carrageenan Foot Pad Test

CBA 3-month-old male mice were used for the test. The reaction was initiated by interaction of carrageenan (a complex glycan) with skin mastocytes, which release histamine and other mediators to attract phagocytes, such as macrophages and neutrophils, constituting cell infiltration accounting for skin edema with a peak intensity after 3–4 h. The mice were injected with 4B8M (250 µg/mouse) intraperitoneally (i.p.) 1 h before carrageenan administration. PGE receptor antagonists AH23848 (EP4 receptor antagonist) and L-798106 (EP3 receptor antagonist) were given at doses of 40 µg/mouse intramuscularly (i.m.) 45 min following 4B8M, and 2% carrageenan solution in 0.9% NaCl was administered subcutaneously (s.c.) into hind foot pads. The footpad thickness was measured after 3 h with a spring caliper with an accuracy of 0.05 mm (Mitutoyo, Kawasaki, Japan).

### 2.9. Contact Sensitivity to Oxazolone

The test was performed as described by others [17], with some modifications. The reaction was initiated by uptake of oxazolone (the antigen) by skin dendritic cells, which migrate then to draining lymph nodes for antigen presentation to T cells. A second exposure to the antigen attracts antigen-specific T cells to the site of antigen deposition by means of chemokines and initiates an effectual phase of this reaction by attraction of neutrophils for antigen elimination. The accompanying inflammatory processes increase edema of the tissue (auricles) with a peak response at 24 h following antigen challenge (the delayed type hypersensitivity). Mice (6 or 7 per experimental group) were shaved on the abdomen, and after 24 h, 100 µL of 0.5% oxazolone in acetone was applied topically. 4B8M peptide was applied as an ointment at a concentration of 0.07–0.1%, topically, on both sides of the ears. The contact sensitivity reaction was elicited 5 days later by application of 50 µL of 1% oxazolone in acetone on both sides of the ears. Reference compounds (Hydrocortisonum^®^, Protopic^®^ and Elidel^®^) were used in commercially available forms in a similar fashion. The compounds were applied 24 h after elicitation of contact sensitivity. The ear edema was measured 24 h later using a spring caliper. The results were presented as an antigen-specific increase of the ear thickness, i.e., the ear thickness of background (BG) mice (given only the eliciting dose of the antigen) was subtracted.

### 2.10. Inflammatory Skin Reaction to Toluene Diisocyanate (TDI)

The test was performed as described elsewhere [18] with minor modifications. This type of antigen predisposes mice to developing cutaneous inflammatory responses dependent on development of antibody response to TDI. Mice, treated topically with subsequent doses of TDI gradually develop higher levels of antibodies. After a sufficient concentration of antibodies is attained, a challenge with antigen (TDI) elicits an inflammatory reaction, beginning with response at 4 h and developing thereafter, generated by antigen-antibody complexes affecting permeability of capillary blood vessels and attracting neutrophils. The mice were shaved on the abdomen (2 × 2 cm area), and after 24 h, 100 µL of 3% TDI in acetone was applied over 3 consecutive days (the sensitizing dose of the antigen). After 14 days, the reaction was elicited by application of 50 µL of 0.3% TDI (the eliciting dose of antigen) on both sides of the ears. The procedure was repeated 5 times every 3 days, on days 14, 17, 20, 23, and 27. Twenty-four hours after each challenge with TDI, the ear thickness was measured with a spring caliper, and 24 h after the last elicitation of the reaction (28th day of the test), the parameters of the inflammatory reaction were determined. 4B8M peptide was applied in the form of a 0.1% ointment, topically, on both sides of the ears (total volume of 50-100 µL per one ear), 1 h following each challenge with TDI. The reference drugs (Protopic^®^ and Elidel^®^) were applied in a similar way.

### 2.11. Ovalbumin (OVA)-Induced Pleurisy

The previously described method was used [19]. The immunization of mice with OVA preferentially elicits humoral immune response, characterized by the presence of antigen-specific Th2 type cells and IgE antibodies. A second challenge with OVA in an intrapleural cavity triggers an inflammatory response in pleural cavity and lungs initiated by cytokines (IL-5) and mediators from degranulated eosinophils and mastocytes. The mice were sensitized i.p. with 50 μg of OVA in Maalox (aluminii hydroxydum 3.5 g, magnesi hydroxydum 4.0 g in 100 mL; Rhone-Poulenc Rorer, Paris, France) as an adjuvant. After 14 days, the mice were injected with 12.5 μg OVA in 50 μL of 0.9% NaCl into the pleural cavity by means of a syringe with a 2-mm needle length. Mice from the BG group were given only a second (eliciting) dose of OVA; 2 h before administration of the eliciting dose of the antigen, the mice were given per os (by gastic gavage) 250 μg of 4B8M in olive oil. The reference compounds—dexamethasone (125 μg/mouse) and indomethacin (250 μg/mouse)—were also given per os. In another experiment, the mice were given 4B8M peptide at a dose of 250 μg/mouse i.p. On the next day, mice were subjected to halothane anesthesia and bled via the retroorbital plexus, followed by cervical dislocation. The draining lymph nodes and exudates from the inflamed pleural cavity were also isolated.

### 2.12. Dextran Sulfate-Induced Colitis

The test was performed according to a previously described method [20], with some modifications. This model mimics colitis described in patients of still unknown etiology and has no immune specific basis. Rather, a destruction of mucosal colonic membrane and disintegration of crypts results from a direct cytotoxic effect of dextran sulfate on colonic mucosa. C57Bl/6 mice were given a 4% solution of dextran sulfate in tap drinking water acidified to pH 3.5 (with hydrochloric acid to limit growth of *Pseudomonas* species) for 6 days. Then, the mice were given only the acidified water. The tetrapeptide 4B8M was administered to mice intragastrically, by means of a stomach tube, at a dose of 200 µg in 0.2 mL, daily on days 1–5. Pentasa^®^ was given in the same way at a dose of 1.5 mg (0.15 mL). The control group received an appropriately diluted DMSO solution (as in 4B8M preparation). On day 12 of the experiment, the mice were subjected to halothane anesthesia and bled via the retroorbital plexus, followed by cervical dislocation. The thymuses and colon were isolated.

### 2.13. Determination of Thymocyte and Lymph Node Cell Number and Viability

Thymuses, spleen, and draining lymph nodes were isolated and placed in disposable Petri dishes with sterile, cold PBS. The cells were released from the lymphatic organs by passing them through a nylon mesh. Then, the cells were resuspended in PBS containing 0.2% trypan blue. The numbers of viable and dead cells were counted in a Bürker hemocytometer in a light microscope. The total number of cells, number of viable and dead cells, and percentage of viable and dead cells were shown. The mean values ± standard error (SE) for each group without subtraction of BG values were shown.

### 2.14. Analysis of Peripheral Blood Cell Picture

The mice were subjected to halothane anesthesia, and blood was obtained from the retroorbital plexus. The blood smears were prepared on microscopic glasses. After drying out, the smears were stained with Giemsa and May-Grünwald reagents. The smears were subsequently evaluated at 1000× magnification (in immersion oil) in a Nicon ATC 2000 microscope. Up to 100 cells were counted per glass/preparation. The results were presented as a percentage of main cell types in the peripheral blood (mature neutrophils and neutrophil precursors—bands, eosinophils, lymphocytes, and monocytes). Mean values for each group were shown.

### 2.15. Determination of Cell Composition in the Pleural Exudates

The exudates from pleural cavities were obtained by adding with a pipette to each cavity 1 mL of 0.9% NaCl and aspiration of the liquid to tubes; 20 µL of the cell suspension was diluted 20× with Turk’s solution, and cells were counted in Bürker’s hemocytometer. Then, the exudates were centrifuged and supernatants saved at −20 °C for cytokine determination. From the cell pellets, smears were prepared on microscopic glasses, stained with Giemsa and May-Grünwald reagents, and inspected in 1000× magnification (in immersion oil). The following cell types were taken into account: mastocytes, monocytes, macrophages, lymphocytes, basophils, eosinophils, neutrophils, and immature forms of neutrophils (bands). The results were presented as a percentage of each cell type in the exudates cell population.

### 2.16. Histological Analysis

The mice auricles, skin fragments, lungs, and colons were fixed in 4.0% neutral buffered formalin for 48 h, dehydrated in an alcohol series, cleared in xylene, and embedded in paraffin. Paraffin blocks were sliced in a microtome (Microm HM310, Walldorf, Germany) into 6-µm sections. The paraffin sections were stained with hematoxylin and eosin (H&E) and with toluidine blue in order to detect mastocytes in the mice skin. The histological and quantitative (in case of mice auricles) analysis was conducted in a light microscope Nikon Eclipse 80i with the aid of imagine software NIS-Elements at 400× magnification. The number of neutrophils, macrophages, lymphocytes, and mast cells in the connective tissue of auricles in random high-power fields (HPF, area 0.071 mm^2^) was estimated. For each studied group, 25 determinations of the abovementioned cell types were performed. Mean values for each group without subtraction of the background values were shown.

### 2.17. Statistics

Each experimental group consisted of 7–8 mice, and each experiment was repeated 3–4 times. The results are presented as mean values ± SE (standard error). A Brown–Forsyth’s test was used to determine the homogeneity of variance between groups. In addition, the occurrence of dependencies between group means and variances were checked. When the above assumptions (normal or symmetric distribution of variables, homogeneity of variance, and lack of dependencies between means and variances) were completed, an analysis of variance (one-way ANOVA) was applied, followed by post hoc comparisons with Tukey’s test to estimate the significance of the differences between groups. Nonparametric data were evaluated with the Kruskal–Wallis’ (K-W) analysis of variance, as indicated in the text. Significance was determined at *p* < 0.05. A statistical analysis was performed using STATISTICA 7 for Windows.

## 3. Results

### 3.1. Preliminary Studies

**Figure 1:**
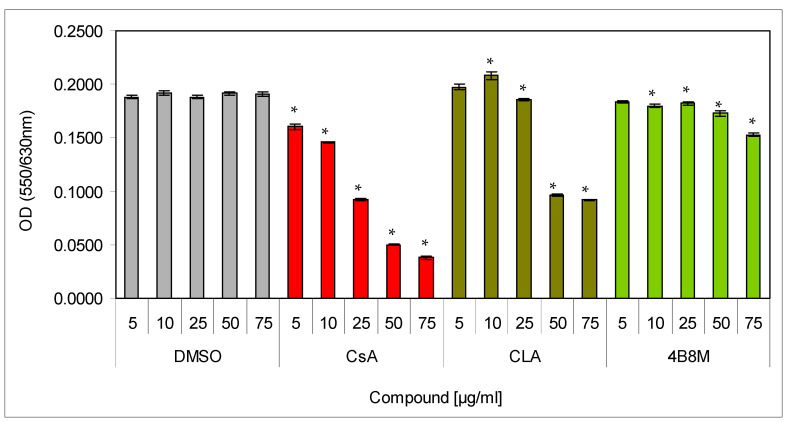
Toxicity of the peptide against mouse splenocytes: Splenocytes, resuspended in the culture medium, were cultured for 24 h in a cell culture incubator with the preparations at indicated concentrations. Cell survival was determined by the MTT (93-[4-dimethylthiazol-2-yl]-2,5-diphenyltetrazolium bromide) colorimetric method. Mean values OD ± SE for respective groups are presented; * dimethylsulfoxide (DMSO) vs. compounds *p* < 0.05 (ANOVA).

The 4B8M peptide was tested for its immunosuppressive activity and potential therapeutic utility in a number of in vitro and in vivo mouse models. Low cell toxicity is a desirable feature of a potential therapeutic drug. Therefore, we initially evaluated the cytotoxic effect of the peptide, together with reference compounds (CsA and CLA), in the 24 h cultures of mouse splenocytes. The compounds were tested at the 5–75 µg/mL concentration range. The cell viability was determined using the MTT colorimetric method. The results (Figure 1) clearly revealed strong, dose-dependent toxicity of CsA already at 25 µg/mL. The toxicity of CLA was also substantial and evident at 50 µg/mL (about 50% cell death). In contrast, the 4B8M peptide demonstrated only a trace toxicity at 50 µg/mL and only about 20% inhibition of cell survival at 75 µg/mL.

Other results (see the Supplementary Materials) showed that 4B8M inhibited mitogen-stimulated proliferation: mouse lymphocytes in the spleen (Figure S1A,B) and human PBMC (Figure S2). The peptide also inhibited LPS-induced tumor necrosis factor alpha (TNF-α) (Figure S3A) production by human whole blood cell cultures. On the contrary, the peptide increased IL-6 production by human whole blood cell cultures (Figure S3B). The peptide strongly suppressed in vitro (Figure S4A) and in vivo humoral immune response to sheep red blood cells (SRBC) (Figure S4B) and cellular immune response to OVA at intraperitoneal administration (Figure S5).

### 3.2. Contact Sensitivity to Oxazolone

**Figure 2:**
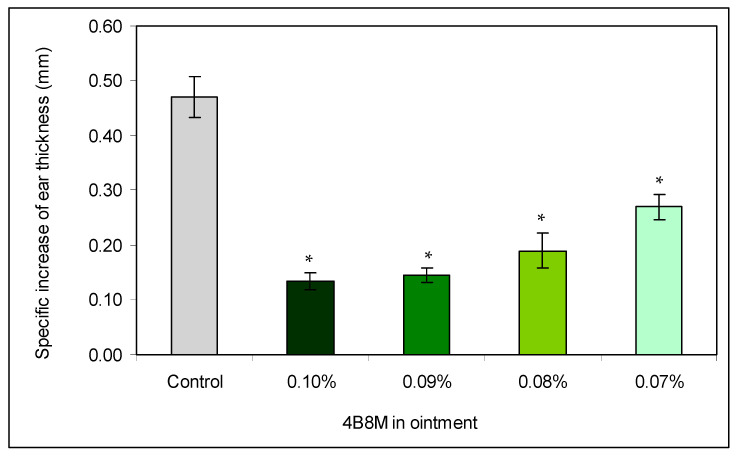
The dose-dependence efficacy of the peptide in inhibition of oxazolone-induced ear edema: the mice were topically sensitized with oxazolone. The reaction was elicited 5 days later by application of oxazolone on both sides of the auricles. BG mice were not sensitized and treated only with the eliciting dose of the antigen. Control: inflamed mice were not treated with the peptide. The peptide, in ointment at a concentration of 0.07–0.1%, was applied on the auricles on each side of the ear 24 h after application of the eliciting dose of oxazolone. The measurement of the ear edema was performed 24 h later, and the results are presented as the antigen-specific increase of the edema (by subtracting BG values). Mean values ± SE for respective groups are presented. * Control vs. 4B8M *p* < 0.05 (ANOVA).

In a first experiment, we wished to check the concentration-dependent therapeutic efficacy of the peptide to suppress antigen-specific auricle edema at topical administration. Figure 2 presents the effects of treatment of mice 24 h after application of the eliciting dose of oxazolone with decreasing concentrations of the peptide on antigen-specific increases of the ear edema. The results indicate that 0.1 and 0.09% ointments cause almost identical decreases of the ear edemas (by 71 and 69%, respectively). Lower peptide concentrations are less effective (59.5 and 43% inhibition).

**Figure 3:**
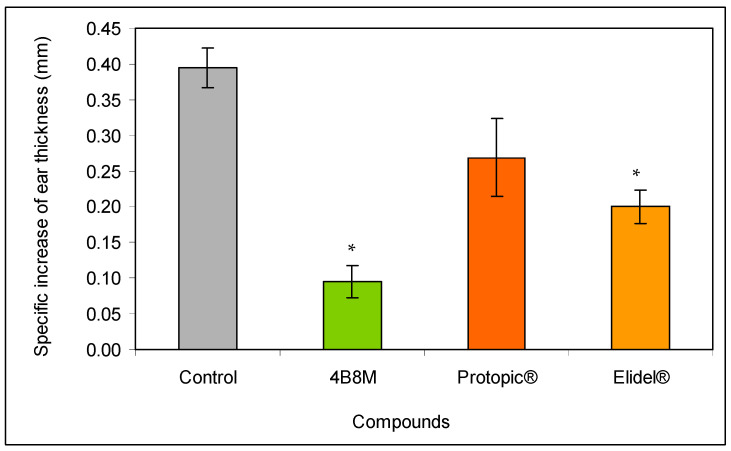
Effect of the 4B8M peptide and the reference drugs on antigen-specific increase of oxazolone-induced ear edema: the experiment was performed as described in Figure 2. The mice were treated topically with 4B8M (0.1% ointment) and the reference drugs at 24 h following elicitation of the antigen-specific reaction to oxazolone. The measurement of the ear edema was performed 24 h later, and the results are presented as the antigen-specific increase of the edema (by subtracting BG values). Mean values ± SE for respective groups are presented. * Control vs. compounds *p* < 0.05 (test of K-W).

Next, we compared the efficacy of the peptide in diminishing ear edema with commercially available preparations. The topical application of the peptide as a 0.1% ointment on auricles on the day of the maximal inflammatory reaction to oxazolone (24 h after elicitation of the inflammatory reaction) caused a large decrease (by about 80%) of the ear edema (Figure 3). A weaker effect was observed for Elidel^®^ containing pimecrolimus as an active ingredient (about 50% inhibition) and Protopic^®^ containing tacrolimus as an active ingredient (30% inhibition).

**Figure 4:**
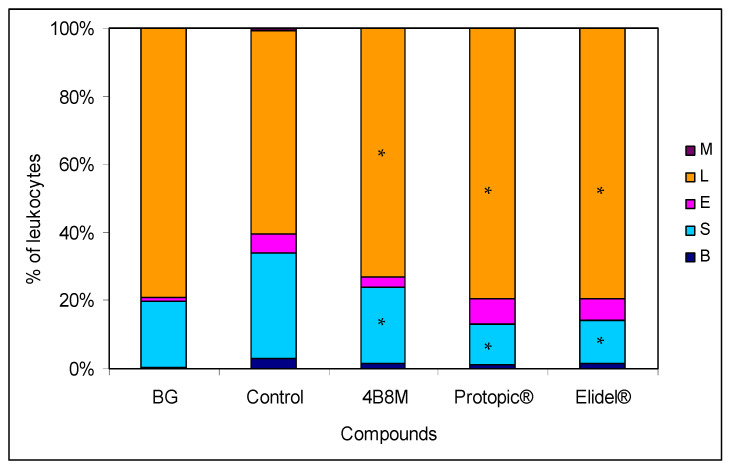
Effect of 4B8M and the reference drugs on peripheral blood cell composition of oxazolone-treated mice: the blood samples derived from mice bled 24 h after application of the compounds. The blood smears were stained with Giemsa and May-Grünwald reagents and evaluated at 1000× magnification. Up to 100 cells were counted per preparation. The results were presented as a percentage of main cell types in the peripheral blood: mature neutrophils (segments, S), neutrophil precursors (bands, B), eosinophils (E), lymphocytes (L), and monocytes (M). Mean values for each group are presented. * Control vs. compounds *p* < 0.05 (ANOVA).

4B8M and Elidel^®^ also lowered the total number (viable and dead) of lymph node cells to the level observed in the BG group (Figure S6A), and these compounds, in contrast to Protopic^®^, lowered the content of dead cells in these cell populations (Figure S6B). Interestingly, Protopic^®^ elevated the number of cells in the draining lymph nodes in relation to the untreated control mice. In the blood picture of the untreated mice (control group), we observed an increase in the percentage of cells of myelocytic lineage (mature neutrophils, bands, and eosinophils) from 20% (in the BG mice) to 40% in the 4B8M group (Figure 4).

The comparison of the anti-inflammatory effects of 4B8M and hydrocorticosterone in this model are presented and in the Supplementary Materials (Figures S7–S9). The normalizing effects of the peptide on the inflammatory skin histological changes induced by oxazolone are presented in Figures S10 and S11.

### 3.3. Skin Inflammation to Toluene Diisocyanate

**Figure 5:**
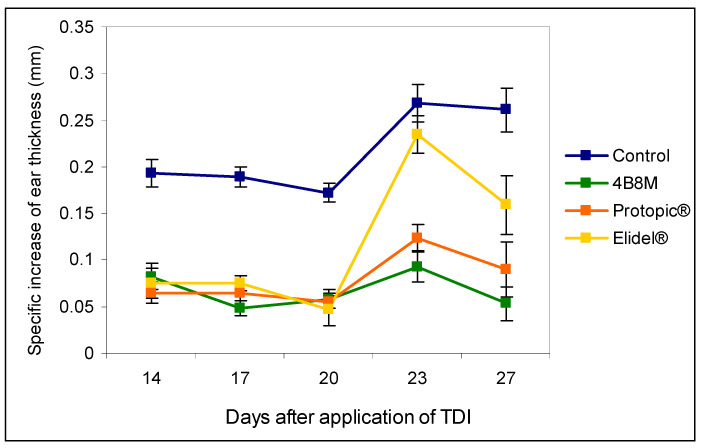
The effects of the 4B8M peptide and the reference drugs on the antigen-specific increase of the ear edema to toluene diisocyanate (TDI) (24-h measurement): mice were immunized with TDI over 3 consecutive days. After 14 days, the reaction was elicited with TDI on the ears. The procedure was repeated 5 times every 3 days. Twenty-four hours after each challenge with TDI, the ear thickness was measured. 4B8M was applied topically in 0.1% ointment on both sides of the ear 1 h following each challenge with TDI. The reference drugs were applied in a similar way. The antigen-specific increase of the ear edema (by subtracting BG values) as mean values ± SE for respective groups is presented. *p* < 0.05 for day 14: control vs. 4B8M, Protopic^®^, and Elidel^®^; day 17: control vs. 4B8M, Protopic^®^, and Elidel^®^; day 20: control vs. 4B8M, Protopic^®^, and Elidel^®^; day 23: control vs. 4B8M, Protopic^®^, and Elidel^®^; and day 27: control vs. 4B8M, Protopic^®^, and Elidel^®^ (repeated measures ANOVA).

The results presented in Figure 5 show the effects of the peptide and the reference compounds on the ear edema upon subsequent applications of the eliciting dose of TDI. The figure illustrates the results of the edema determinations after 24 h following each application of the eliciting dose of the antigen. The results show that both the peptide and Protopic^®^ effectively inhibited the antigen-specific increase in ear edema. The inhibitory effect of Elidel^®^ was observed only at first and last application of TDI.

**Figure 6:**
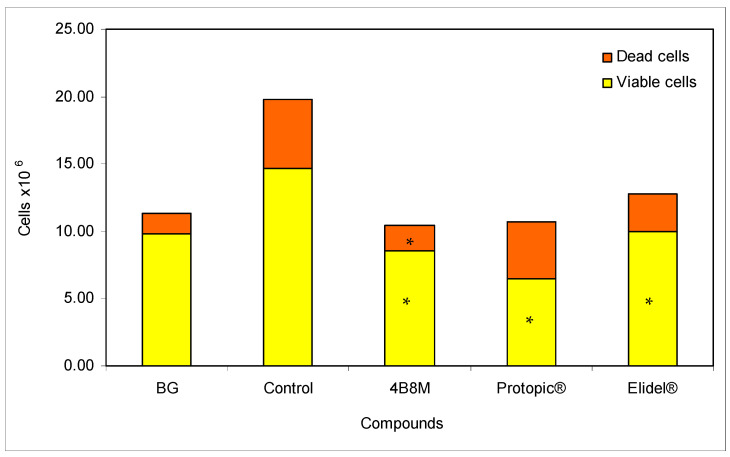
Total numbers of viable and dead cells in the draining lymph nodes: the experiment was performed as described in Figure 5. The number of cells was determined 24 h after the last elicitation of the reaction (28th day of the test). The results are presented as 10^6^ cells for each group (mean values ± SE). * Control vs. compounds *p* < 0.05 (viable cells ANOVA; dead cells test of K–W).

Evaluation of the effects of the compounds on the number and viability of cells in the draining lymph nodes was accomplished 24 h after application of the last, eliciting the dose of TDI antigen. The results shown in Figure 6 indicate that all compounds effectively reduce the cell numbers in the following order: 4B8M > Protopic^®^ > Elidel^®^. In addition, the content of dead cells in the lymph nodes was the lowest in the case of 4B8M treatment. The peptide also decreased the number of leukocytes in the circulating blood (Figure S12), normalized blood cell composition (Figure S13), and lowered the permeability of skin vessels (Figure S14). The suppressive effects of 4B8M on the inflammatory skin processes analyzed by histologists are shown in Figure S15 (hematoxylin and eosin staining and toluidine blue staining), Figure S16 (quantitative analysis of inflammatory cells), and Figure S17 (thickness of the epidermis).

The effects of 4B8M on nonspecific skin reactions to salicylic acid and carrageenan are presented in the Supplementary Materials (Figures S18–S29).

### 3.4. Nonspecific Colon Inflammation Induced by Dextran Sulfate

**Figure 7:**
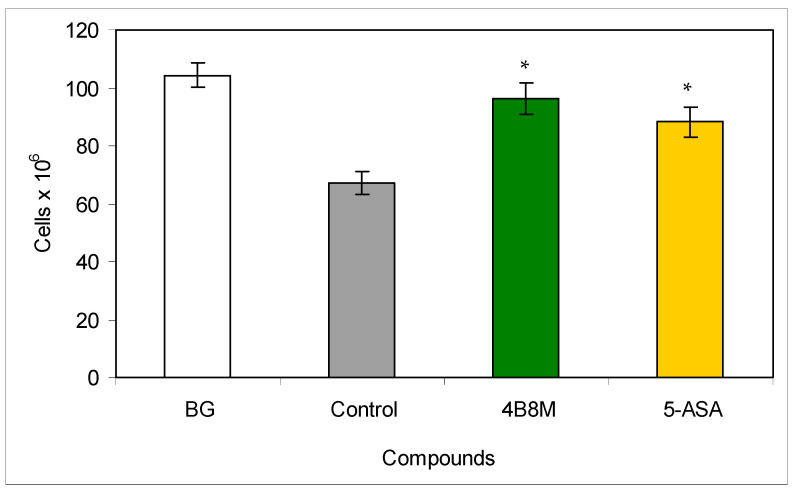
Effects of the compounds on the number of thymocytes in mice with dextran sulfate-induced colitis: the mice drank a 4% solution of dextran sulfate in acidified water for 6 days (days 0–5). The peptide 4B8M and reference drug Pentasa^®^ (5-ASA) were administered to mice intragastrically by a stomach tube daily on days 1–5. On day 12 of the experiment, the thymuses were isolated and the cell numbers were counted. The results are given in 10^6^ cells per thymus (mean values ± SE). * Control vs. compounds *p* < 0.05 (ANOVA).

Treatment of mice with dextran sulfate is associated with the release of endogenous steroids leading to loss of thymocytes [21]. In our experiment, the number of thymocytes in the dextran sulfate-treated group was significantly decreased as compared to untreated mice (6.7 vs. 10.4 × 10^7^). The application of 4B8M was protective because of only a nonsignificant drop in thymocyte number (9.6 × 10^7^). 5-ASA was less effective (a decrease to 8.8 × 10^7^) (Figure 7). The peptide also restored a reduced level of IL-1β in the colonic tissue (Figure S30).

**Figure 8:**
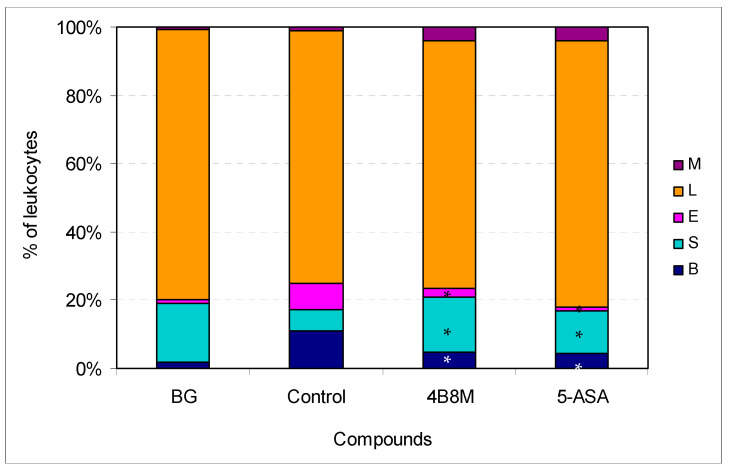
Effect of the preparations on blood cell type composition in mice with chemically induced colitis: the experiment was performed as described in Figure 7. On day 12 of the experiment, the mice were bled and the smears evaluated; up to 100 cells were counted per preparation. The results are presented as the percentages of main cell types: mature neutrophils (segments, S), neutrophil precursors (bands, B), eosinophils (E), lymphocytes (L), and monocytes (M). Mean values for each group are shown. * Control vs. compounds *p* < 0.05 (ANOVA).

The effects of the preparations on the altered blood cell picture is presented in Figure 8. In the dextran sulfate-treated mice, a significant increase of the band form (from 1.89 to 11.0%) and eosinophils (from 1.11 to 7.75%) levels was observed. The peptide and 5-ASA markedly normalized the proportions between the cell type content.

The effects of 4B8M and 5-ASA on the colon structure are presented in the Supplementary Materials (Figure S31) and demonstrate effective and protective effects of 4B8M, comparable to those of 5-ASA, on inflammation parameters and the histological picture of the colon. The peptide also showed some advantage over 5-ASA regarding integrity of the colon wall.

### 3.5. Allergic Pleurisy Induced by Ovalbumin

**Figure 9:**
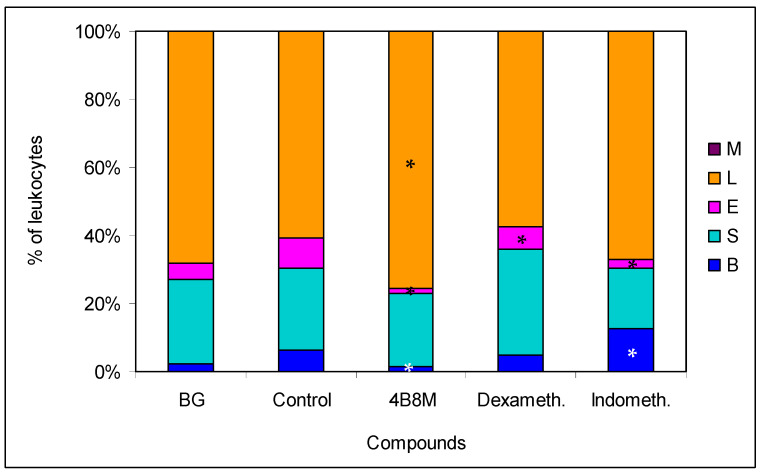
Effects of the compounds of blood cell type composition in mice with pleurisy: the mice were sensitized intraperitoneally (i.p.) with 50 μg of ovalbumin (OVA) in Maalox as an adjuvant. After 14 days, the mice were injected with 12.5 μg of OVA into pleural cavity. Two hours before administration of the eliciting dose of the antigen, the mice were given per os 250 μg of 4B8M. The reference compounds—dexamethasone (125 μg/mouse) and indomethacin (250 μg/mouse)—were also given per os. On the next day, mice were bled and the smears were analyzed; up to 100 cells were counted for each preparation. The results are presented as the percentages of main cell types: mature neutrophils (segments, S), neutrophil precursors (bands, B), eosinophils (E), lymphocytes (L), and monocytes (M). Mean values for each group are shown. * Control vs. compounds *p* < 0.05 (ANOVA).

**Figure 10:**
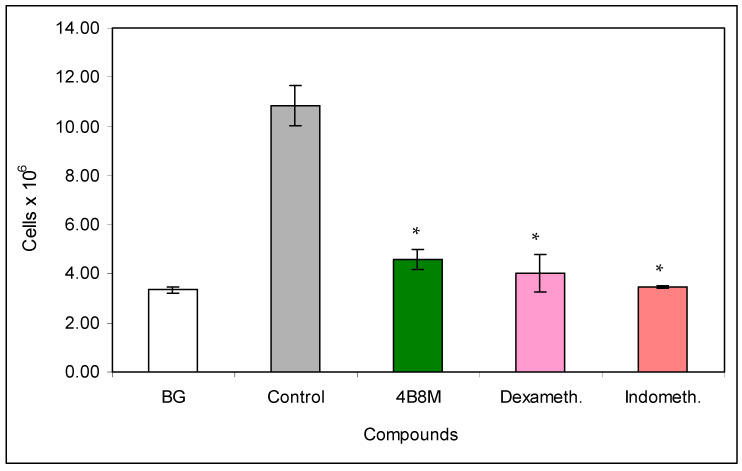
Effects of the compounds on cell numbers in the pleural exudates: the experiment was performed as described in Figure 9. The exudates from pleural cavities were centrifuged, cell pellets were diluted with Türk’s solution, and the cells were counted in a Bürker hemocytometer. The results are shown as 10^6^ cells (mean values ± SE). * Control vs. compounds *p* < 0.05 (ANOVA).

**Figure 11:**
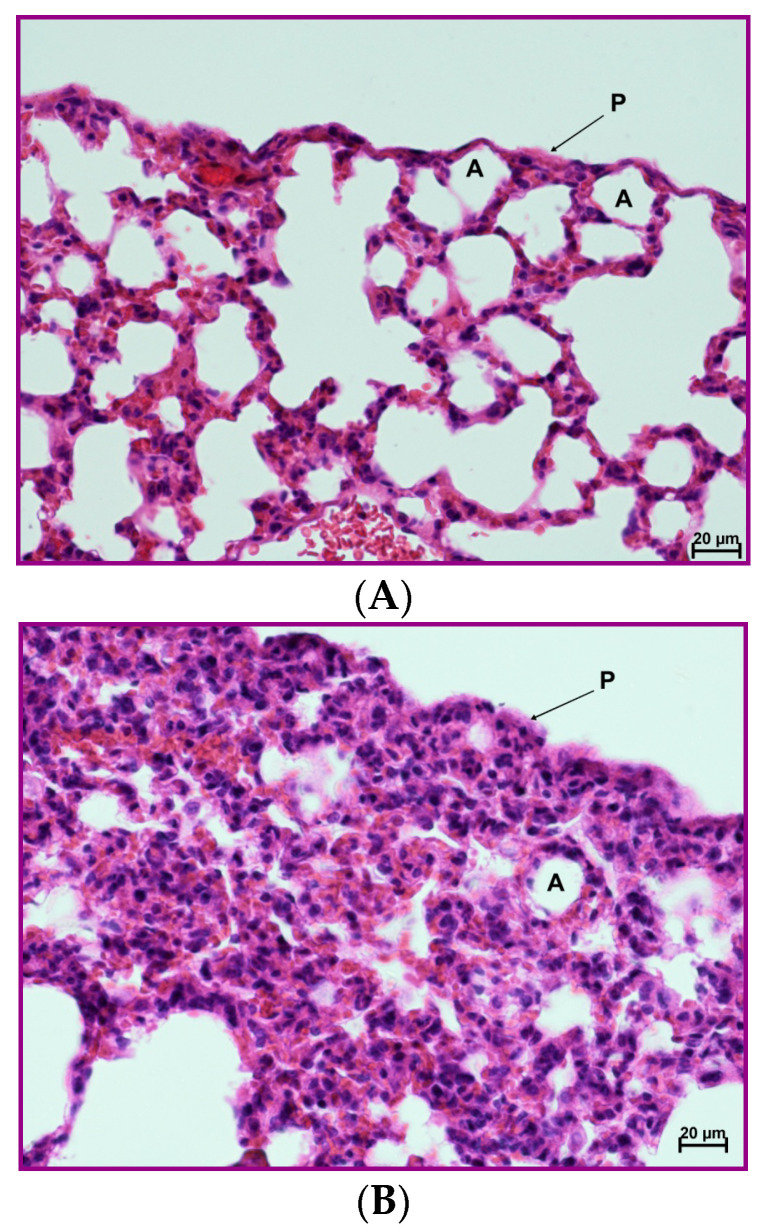

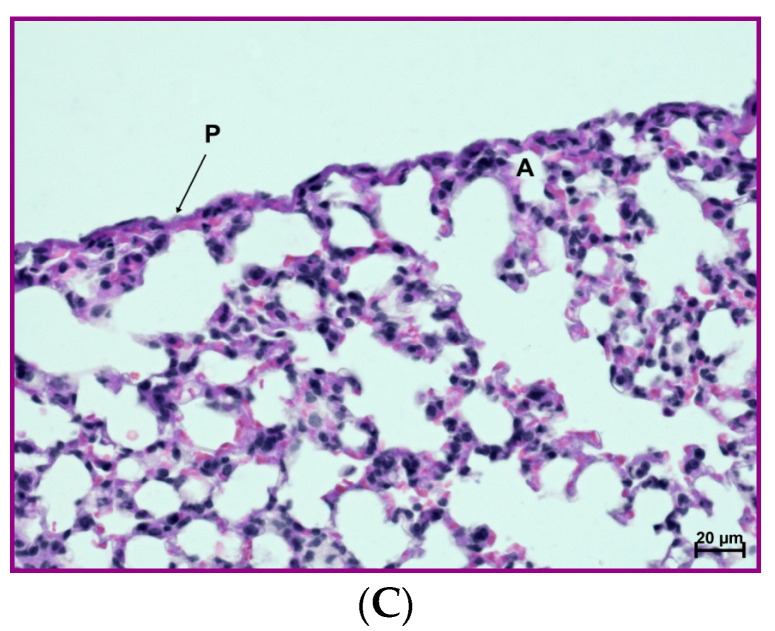
Protective effect of the 4B8M peptide on histological changes in the model of OVA-induced pleurisy: the experiment was performed as described in Figure 9, but mice were given 4B8M 250 μg i.p. (**A**) Lung of the naive (BG) mice: normal appearance. (**B)** Lung of the mice with fully blown inflammation: interstitial and pleural inflammation. (**C)** Lung of the mice pretreated with 4B8M i.p.: a silencing of the inflammatory processes was observed; detailed description of the histological analysis is in the text. Abbreviations: P—pleura, A—pulmonary alveolus; magnification 400×; scale bars, 20 μm.

In the model of OVA-induced pleurisy, we use dexamethasone and indomethacin as reference anti-inflammatory drugs. Analysis of the blood picture (Figure 9) clearly shows that the normalization of blood cell composition was best for the 4B8M peptide. Dexamethasone was without effect, and indomethacin increased the rate of myelopoiesis (high content of neutrophil precursors). Among the applied compounds, dexamethasone was the most effective in lowering the number of circulating leukocytes (Figure S32). All compounds had the ability to lower cell numbers in the pleural exudates (Figure 10). The cell numbers in pleural exudates were significantly elevated in inflamed control mice (1.1 × 10^7^ vs 2.1 × 10^6^ in naive mice and 3.3 × 10^6^ in the BG group). All applied compounds evenly lowered the cell numbers near the values in BG mice. Pleural exudates from fully blown inflammation contained a 5-fold higher number of mastocytes, as compared with naive mice, and the majority of them were de-granulated. All compounds reduced the number of mastocytes in the pleural cavities; however, the highest content of intact (non-degranulated) mastocytes was found in mice treated with the 4B8M peptide (Figure S33)**.** All compounds were equally effective in reducing cell numbers in the draining lymph nodes (Figure S34). In the histological analysis, we present the photographs of lung sections of mice with OVA-induced pleurisy and a protective effect of 4B8M administration (at dose 250 μg/mouse) i.p. (Figure 11) and per os (Figure S35).

In naive mice (Figure 11A), no changes in the interstitium were observed. Likewise, no abnormalities were found within the bronchial and alveolar tree. In lung cross sections, pleura was seen, composed of a single cell layer (mesothelium) and situated below a very thin connective tissue layer. The histological picture of mice with OVA-induced pleurisy presented interstitial lung inflammation (Figure 11B) with infiltrations by lymphocytes and macrophages in the connective, peribronchial, and perivascular tissue of lung lobules. In addition, extensive inflammatory infiltrations in the connective, interlobulated tissue were observed. As a result of cell infiltration, the interalveolar septa were thickened. In the lumena of bronchi and bronchiole, a serous secretion with the inflammatory cells was found. A pleural inflammation was also confirmed. Under mesothelium, numerous inflammatory cells (mainly lymphocytes and macrophages) and very extensive extravasations were noted. As a result of the ongoing inflammatory process, a thickening of the connective, subpleural tissue was registered. Proliferation of the connective tissue led to deformation of the alveolar structure. In mice given 20 µg of the peptide, a silencing of the inflammatory processes was observed, both around bronchi, bronchioli, and blood vessels as well as in the subpleural area (Figure 11C). The histological picture presented a normal stromal lung architecture. Small subpleural infiltrations occurred rarely and had a local character. The preventive action of the peptide consisted mainly of lowering leukocyte, exudates cells, and lymph node cell numbers and normalization of the peripheral blood cell picture and exudates cell picture.

### 3.6. The Mechanism of Action

**Figure 12:**
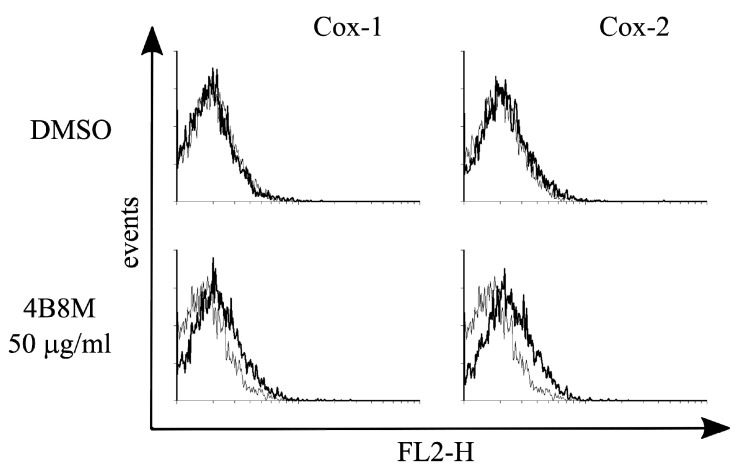
Effect of 4B8M on induction of COX-1 and COX-2 in the human keratinocyte KERTr cell line.

The results shown in Figure 12 indicate that intracellular expression of COX-1 and, to a higher degree, of COX-2 in the KERTr human epidermal cell line was enhanced by the peptide. Such effects were not registered in Jurkat cell as well as in PBMC (data not shown).

**Figure 13:**
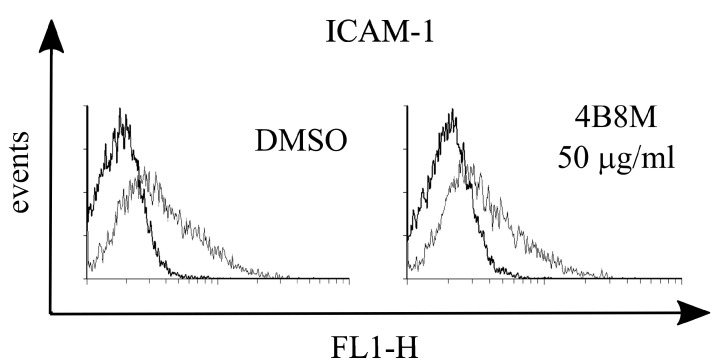
Effect of 4B8M on expression of ICAM-1 on human peripheral blood mononuclear cells (PBMC).

**Figure 14:**
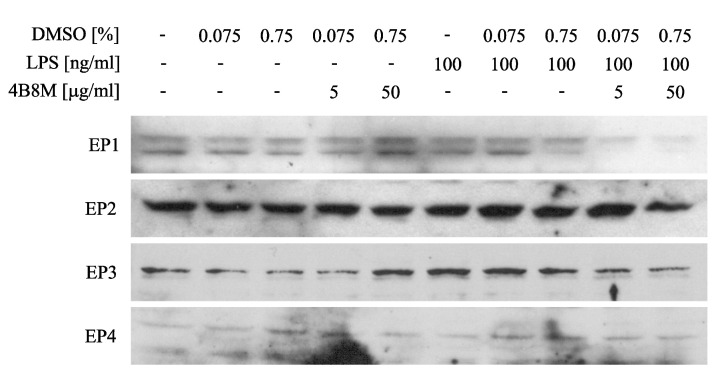
Effect of 4B8M on expression of E-prostaglandin (EP) receptors on Jurkat cells determined by Western blot.

**Figure 15:**
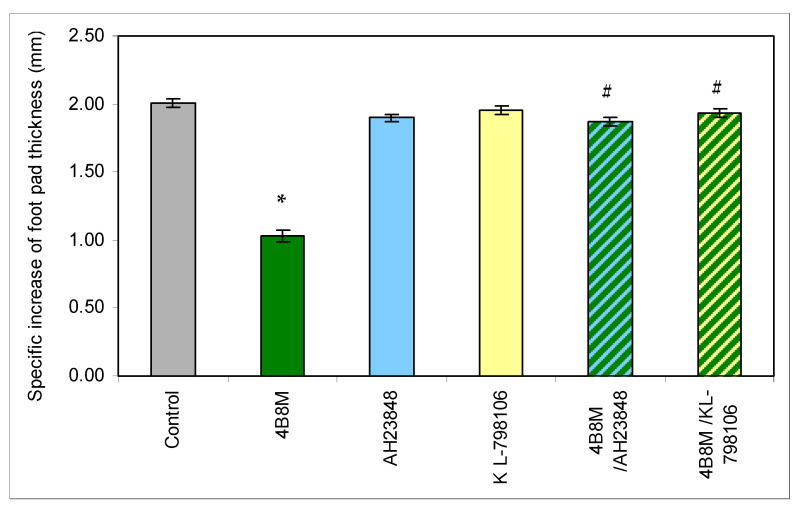
Antagonists of EP4 and EP3 receptors for PGE2 abolish the suppressive action of the peptide in carrageenan-induced foot pad edema in mice. The mice were injected with 4B8M (250 µg/mouse) i.p. 1 h before s.c. carrageenan administration. PGE receptor antagonists AH23848 (EP4 receptor antagonist) and L-798106 (EP3 receptor antagonist) were given at doses of 40 µg/mouse intramuscularly (i.m.) 45 min following 4B8M. The footpad thickness was measured after 3 h with a spring caliper, and the results are presented as the antigen-specific increase of the edema (by subtracting BG values). Mean values ± SE for respective groups are presented. * Control vs. 4B8M *p* < 0.05; # 4B8M vs. 4B8M/AH23848; 4B8M vs. 4B8M/KL-798106 *p* < 0.05 (test of K-W).

Since PGE2 may cause inhibition of ICAM-1 expression, the adhesion molecule that plays a key role in cell adhesion and cell trafficking [22], we tested the effect of 4B8M on LPS-induced ICAM-1 expression on human PBMC. Figure 13 shows that the peptide at a low concentration (5 µg/mL) lowered the expression of ICAM-1 on these cells. Figure 14 demonstrates, in turn, that the expression of EP receptors was differently regulated in Jurkat cells by the peptide, i.e., EP1 and EP3 receptors were strongly downregulated, whereas the expressions of EP2 and EP4 receptors were not affected. The results shown in Figure 15 revealed that the suppressive action of 4B8M in the carrageenan-induced foot pad edema test is blocked by pretreatment with antagonists of EP4 and EP3 receptors for PGE2. Other results indicate that 4B8M moderately inhibited in a dose-dependent manner IL-2 driven proliferation of CTLL-2 mouse cytotoxic T cells (Figure S36).

## 4. Discussion

In the preliminary investigations (presented in more detail in the Supplementary Materials), we found that the cyclic peptide 4B8M showed antiproliferative and immunosuppressive properties. Although the antiproliferative effects of the peptide were weaker as compared with CsA and CLA, a clear advantage of the peptide over the reference compounds was a negligible toxicity against mouse splenocytes, even at high doses (Figure 1). The inhibition of LPS-inducible TNF-α production and stimulation of IL-6 activity in human whole blood cultures suggested anti-inflammatory properties (Figure S3). The findings were similar to previous observations on the inhibitory actions of CLA on IL-1 production and post-adjuvant polyarthritis in rats [3] and suggested that the peptide shared the anti-inflammatory properties of native, parent CLA. Interestingly, comparison of the suppressive effects of the peptide and CsA in the models of humoral (Figure S4) and cellular (Figure S5) immune responses in vivo revealed that these types of immune responses were more strongly inhibited by the peptide. These differences could be due to a better bioavailability of the peptide in vivo in comparison with CsA, the reference compound, as well as related to different mechanisms of action.

The investigation demonstrated a high efficacy of 4B8M in amelioration of the contact sensitivity to oxazolone in mice that surpassed the effects of the reference preparations (Figure 3 and Figure 4, Figures S6 and S10). In this experimental model, Protopic^®^ (tacrolimus) exhibited an undesirable action. The application of tacrolimus in atopic dermatitis is generally well-tolerated but not devoid of side-effects such as skin irritation, particularly in the first stage of therapy [23]. Elidel^®^ (pimecrolimus), on the other hand, is less irritable [24] and permeates to a lesser degree the circulation of patients as compared to tacrolimus [25]. However, both topical tacrolimus and pimecrolimus transiently induced mast cell degranulation in mouse skin [26], which may account for their decreased efficacy in diminishing skin inflammation, observed in our study, in comparison to 4B8M. Also consistent with the transient pro-inflammatory properties of tacrolimus were our observations on the high mortality of lymphocytes in the draining lymph nodes (Figure S6), increased skin vessel permeability, and high numbers of infiltrating neutrophils in the ear connective tissue (Figure S10). In addition, topical calcineurin inhibitors disrupt the skin barrier, which may lead to susceptibility to infection [27]. Another limitation of pimecrolimus is that its action is mostly restricted to the effectual phase of contact sensitivity [28].

4B8M also very effectively suppressed the cutaneous, humoral immune response to TDI (Figure 5 and Figure 6, Figures S12–S17). Unlike the peptide, the reference compounds showed a differential action, consistent with the literature. Tacrolimus had a significant effect on allergic skin inflammation in mice [29]. Pimecrolimus, in turn, was weakly effective in rats in inhibiting anti-KLH (keyhole limpet hemocyanin) antibodies, in contrast to tacrolimus and CsA [30]. Moreover, no significant improvement was achieved following application of pimecrolimus in the case of facial skin changes [31].

4B8M also proved to be very effective in diminishing symptoms of nonspecific skin irritation by salicylic acid (Figures S18–S23). In this model, tacrolimus was less efficacious by showing some cytotoxicity, as also reported by others in a similar mouse model [26]. Sensitizing properties of tacrolimus were also found in acute skin inflammation in mice [32]. Our unpublished results did not reveal any sensitizing properties of 4B8M when the peptide was applied topically to mice, daily, for a long period.

In the air pouch model of carrageenan-induced inflammation, the peptide was more efficient, as compared to the reference drugs, in diminishing symptoms of inflammation (Figures S24–S29). The model is considered to be a clinical equivalent of rheumatoid arthritis [33]. 4B8M more effectively normalized the cell composition of circulating blood, activity of mastocytes in the air pouch exudates, and the granuloma layer thickness, as compared with the reference compounds (indomethacin, aspirin, and dexamethasone).

Therapeutic action of 4B8M on chemically induced colitis was comparable with that of 5-ASA (Figure 7 and Figure 8, Figures S30 and S31). We confirmed another finding [34] that the high IL-1β level in the colon reflects a good health condition (as evaluated by the presence of physiologic microflora) of naive mice. That level was lower in control mice, and the peptide and, to a lesser degree, 5-ASA reversed that decrease in IL-1β content (Figure S30). On the other hand, the histological analysis of the colon revealed some toxicity of 5-ASA, in contrast to 4B8M (Figure S31). That was confirmed by our monitoring of the mouse condition since 5-ASA-treated mice sometimes had blood in the stool and diarrhea.

The 4B8M peptide effectively inhibited symptoms of allergic pleural and lung inflammation induced by OVA in the analyzed parameters (Figure 9, Figure 10 and Figure 11 and Figures S32–S35). Of importance, 4B8M administered at a higher dose in olive oil by intragastric gavage was also preventive, suggesting its potential therapeutic use in asthma.

In all experimental models, mice demonstrated increased content of myelocytic cells in their circulation including immature forms of neutrophils and eosinophils. This effect was probably due to the release of endogenous steroids in response to injury related stress [21,35] and effects of some acute phase proteins, such as lactoferrin, which induce corticosterone in operated mice [36]. Therefore, the application of dexamethasone in some models such as carrageenan inflammation and pleurisy (Figure 9 and Figure S26) had no effect on the altered blood cell composition. In the case of hydrocortisone applied in the model of contact sensitivity to oxazolone, the drug even caused a high distortion of blood cell composition (Figure S9) despite good therapeutic efficacy (Figures S7 and S8).

The results derived from the above described models led us to conclude that 4B8M has a decisive anti-inflammatory property, so our investigation on its mechanism of action should be focused on analyzing the genes associated with inflammatory processes. In our preliminary experiments (not shown), a potential effect of the peptide on the expression of 38 genes was confirmed. Of interest, as many as 15 of these were directly linked to the metabolism of arachidonoic acid and at least 10 genes may be indirectly associated with the metabolism of arachidonoic acid, prostaglandins, and prostacyclins. Among 12 proteins involved in regulating the inflammatory process, based on real-time PCR analysis, the peptide lowered LPS-induced EP1 and EP3 receptor cell expression on PBMC and Jurkat T cells. The expression of EP2 and EP4 receptors was, however, not affected. In addition, 4B8M lowered the expression of ICAM-1, the receptor of cell adhesion, and induced expression of COX-2 on keratinocytes. The above described phenomena induced by the peptide were helpful in explaining its effects in the described experimental models since the actions of PGE2 differ depending on the type of EP receptors used [37]. In general, transduction of signals by PGE2 via EP1 and EP3 receptors results in inflammatory reactions whereas engagement of EP2 and EP4 receptors is anti-inflammatory. The ability of 4B8M to induce Cox-2 expression, which leads to PGE2 synthesis, may explain its anti-inflammatory properties in several models. Cox-2 was implicated in the inhibition of inflammatory states by lowering ICAM-1 expression [38] and in the suppression of TNF α-induced chemokine production by keratinocytes [39]. In fact, the inhibition of ICAM-1 expression by the peptide was also found in this investigation. The inhibition by PGE2 of ICAM-1 expression induced by IL-18 in human monocytes involved the EP2/EP4 signaling pathway [22]. The inhibition of ICAM-1 expression on epidermal cells by 4B8M indicates that the effective suppression of skin inflammation may be due to inhibition of inflammatory cell infiltration. Of interest, the expression of these receptors was not affected by the peptide in our study. The suppressive effect of PGE2 via EP2 and EP4 receptors was also confirmed in the case of dendritic cells in which the expression of major histocompatibility complex class II antigens (MHC class II) was downregulated [40]. The above described effects of PGE2 are in agreement in our results from antigen-specific and nonspecific immune responses, where both recognition and presentation of antigens by antigen presenting cells may be inhibited by the peptide. In addition, the inhibition of cell-to-cell contact by lowering ICAM-1 expression may explain the suppressive effect of the peptide when administered during full blown inflammation.

Our preliminary studies demonstrated that the peptide inhibited LPS-induced TNF α but stimulated IL-6 production (Figure S3). This finding is in agreement with data demonstrating that, in LPS-treated mouse neutrophils, PGE2 inhibited TNF α production via EP2 receptors whereas IL-6 production was enhanced [41]. The suppressive effect of 4B8M on TNF α production provides a good explanation for the inhibition of the inflammatory processes where this cytokine plays a key role. Our assumption that the peptide activity involves induction of PGE2 production could be further supported by the fact that PGE2 can inhibit growth of cells dependent on the availability of IL-2 or IL-4 such as CTLL-2 [42] because 4B8M inhibited growth of this line in our study (Figure S36). Again, this effect was dependent on the involvement of EP4 receptors, the expression of which was not affected (downregulated) by the peptide. The results obtained in experimental colitis are also in agreement with our notion that the protective action of 4B8M is mediated via the EP4 receptor. The expression of EP4 receptor mRNA was significantly elevated after treatment with dextran sulfate in a rodent model, and a selective agonist of EP4 (ONO-AE1-329) decreased the pathological changes of colitis [43]. The application of an EP4 agonist was also effective in amelioration of LPS-elicited changes in rat nasal epithelium and in cultured human airway epithelial cells [44] as well as in a model of chemotaxis applying human peripheral blood eosinophils [45]. Thus, a similar mechanism could play a role in amelioration by 4B8M of OVA-specific induction of pleurisy.

More direct evidence for involvement of EP receptors in diminishing inflammatory reactions by the peptide was delivered in the model of carrageenan-induced foot pad inflammation where the application of specific EP receptor antagonists abolished the protective action of the peptide. Whereas the effectiveness of blocking the EP4 antagonist was not disputable, the effective block of the EP3 receptor was rather surprising. However, in some models, treatment with COX-2 and EP3 inhibitors significantly reversed the obesity-associated adipose tissue inflammatory gene and protein expressions [46]. Provided that proinflammatory processes mediated by PGE2 engage EP3 receptors [47], it was not surprising that increased levels of EP3 receptors following treatment with LPS were lowered by 4B8M.

## 5. Conclusions

In summary, the 4B8M peptide appears to mediate its anti-inflammatory activity in two ways: (1) by inducing inhibitory PGE2 action via EP2/EP4 receptors and (2) by lowering expression of EP1/EP3 receptors, it prevents the pro-inflammatory action of PGE2. Thus, the mechanism of action of the peptide differs from the compounds used as reference anti-inflammatory drugs including steroids, calcineurin inhibitors, and selective or nonselective cyclooxygenase inhibitors. Although the anti-inflammatory properties of the peptide were convincingly proved in a number of models, its mechanism of action should be confirmed in greater detail. The peptide has a therapeutic potential in the amelioration of external and systemic inflammatory disorders.

## Supplementary Materials

The following are available online at https://www.mdpi.com/1999-4923/12/11/1030/s1, Preliminary studies: Figure S1A,B: Effect of the peptide on mitogen-induced proliferation of mouse splenocytes. Figure S2: PHA-induced proliferation of human PBMC. Figure S3A,B: LPS-inducible TNF-α and IL-6 production by human whole blood cell cultures. Figure S4A,B: Humoral immune response in vitro and in vivo. Figure S5: Induction phase of the delayed type hypersensitivity to OVA. Contact sensitivity to oxazolone: Figure S6A–C: Number and percentage of viable and dead cells in the draining lymph nodes. Figure S7: Antigen-specific increase of the ear thickness. Figure S8: Number of cells in the draining lymph nodes. Figure S9: The peripheral blood cell composition. Figure S9: Peripheral blood cell composition. Figure S10A–J: Histology of the inflamed auricle. Figure S11: Morphometric analysis of the auricle tissue. Skin inflammation to TDI: Figure S12: Leukocyte numbers in the circulating blood. Figure S13: Peripheral blood picture. Figure S14: Permeability of capillary blood vessels. Figure S15A–J: Histology of the inflammed auricle. Figure S16: Morphometric analysis of the auricle tissue. Figure S17: Thickness of the epidermis. Nonspecific skin inflammation induced by SA: Figure S18: Ear edema. Figure S19: Peripheral blood leukocyte number. Figure S20: Peripheral blood cell type composition. Figure S21: Lymph node cell numbers. Figure S22: Skin blood vessel permeability. Figure S23A–J: Histology of the inflamed auricle. Carrageenan-induced inflammation in air pouches: Figure S24: Cell numbers in the inflamed air pouches exudates. Figure S25: Degranulation of mastocytes in the air pouch exudates. Figure S26: Peripheral blood cell picture. Figure S27: Leukocyte number in the circulating blood. Figure S28A–J: Histology of the inflamed air pouch skin. Figure S29: Thickness of the granuloma tissue. Nonspecific colon inflammation induced by dextran sulfate: Figure S30: IL-1 content in the colon. Figure S31A–E: Histology of the inflamed colon. Allergic pleurisy induced by ovalbumin: Figure S32: Circulating leukocyte number. Figure S33: Intact and degranulated mastocytes in the pleural exudates. Figure S34: Cell numbers in the draining lymph nodes. Figure S35A–J: Histology of the inflamed lungs. Mechanism of action: Figure S36: IL-2-driven proliferation of CTLL-2 mouse cell line.
