# Supplementary material for: Anti-Inflammatory Activity of a Cyclic Tetrapeptide in Mouse and Human Experimental Models
Source: Pharmaceutics. 2020 Oct 28;12(11):1030. doi: 10.3390/pharmaceutics12111030 (PMC7693979; doi:10.3390/pharmaceutics12111030)
Supplement: Supplementary file 1 [file pharmaceutics-12-01030-s001.pdf]

Anti inflammatory activity of a cyclic tetrapeptide in mouse and human experimental models

Michał Zimecki [1,*], Jolanta Artym [1], Wojciech Kałas [1], Leon Strządała [1], Katarzyna Kaleta-Kuratewicz [2], Jan Kuryszko [2], Andrzej Kaszuba [3], Krzysztof Kaczmarek [4], Janusz Zabrocki [4]

[1] Institute of Immunology and Experimental Therapy, Polish Academy of Sciences, ul. Weigla 12, 53-112 Wrocław, Poland; michal.zimecki@hirszfeld.pl (M.Z.); jolanta.artym@hirszfeld.pl (J.A.); wojciech.kalas@hirszfeld.pl (W.K.); leon.strzadala@hirszfeld.pl (L.S.)

[2] Wrocław University of Environmental and Life, ul. C. K. Norwida 25, 50-375 Wrocław, Poland; katarzyna.kaleta-kuratewicz@upwr.edu.pl (K.K.-K.); jan.kuryszko@upwr.edu.pl (J.K.)

[3] Medical University of Łódź, Al. Kościuszki 4, 90-419 Łódź, Poland; andrzej.kaszuba@icloud.com (A. K.)

[4] Łódź University of Technology, ul. Żeromskiego 116, 90-924 Łódź, Poland; janusz.zabrocki@p.lodz.pl (J. Z.); krzysztof.kaczmarek@p.lodz.pl (K. K.)

* Correspondence: michal.zimecki@hirszfeld.pl (M.Z.); Tel: +48 71 370 99 53

Generally – Materials and Methods

Mice

CBA, BALB/c and C57Bl/6 mice of both sexes, 8-12 week old, weighing 19-23 g, delivered by Breeding Centre of Laboratory Animals at the Institute of Occupational Medicine, Łódź, Poland, were used for the study. Mice were housed in a cage at 21-22 ºC with a 12/12-h light/dark cycle and had free access to commercial laboratory chow and filtered tap water. The local ethics committee at the Institute of Immunology and Experimental Therapy, Wrocław, Poland, approved the study (permissions #41/2008, 61/2009, 62/2009, 22/2010, 52/2010, 21/2012, 10/2013, 4/2014)

Reagents

Cyclosporin A (CsA) (Sandimmun, Neoral, Sandoz, Basel, Switzerland) in ampoules, RPMI-1640 medium (Cibi/Life Technologies, UK), fetal calf serum (FCS), Serum-Free Keratinocyte Medium, Bovine Pituitary Extract derived from Gibco Thermo Fisher Scientific (Waltham, MA, USA), glutamate, HEPES, sodium pyruvate, antibiotic and antimycotic solution, cyclolinopeptide (CLA), lipopolysaccharide (LPS) from *Escherichia coli* 0:111, concanavalin A (Con A), phytohemagglutinin A (PHA), ovalbumin (OVA), indometacin, aspirin (acetylsalicylic acid), MTT (93-[4,5-dimethylthiazol-2-yl]-2,5-diphenyltetrazolium bromide), oxazolone, salicylic acid, toluene diisocyanate (TDI), recombinant Epidermal Growth Factor (EGF), carrageenan (C-1013, Lot 102 K0871), AH23848 (A8227, Lot 090M4618V) (EP4 receptor antagonist), L-798106 (L4545, Lot 020M4617V) (EP3 receptor antagonist), Giemsa and May-Grünwald reagents, hematoxylin, eozyne, toluidine blue derived from Sigma-Aldrich (St. Louis, MO, USA), acetone from Acros Organics, Poland, isotonic solution of sodium chloride, buffered with phosphates (PBS) from the Laboratory of General Chemistry, Institute of Immunology and Eperimental Therapy, Wrocław. Narcotan® (halotane) and Pentasa® (5-aminosalicylic acid [5-ASA]) were from Ferring-Leciva (Czech Republic). Hydrocortisonum® (hydrocortisone acetate) 1% creme from Jelfa (Poland), Dexaven® (dexamethasone) from Polfa-Warszawa (Poland), Protopic® (tacrolimus) 0.1% ointment from Astellas (Ireland), Elidel® (pimecrolimus) 1% creme from Novartis and DMSO (dimethylsulfoxide) from Fluka, Poland. Sheep red blood cells (SRBC) were from Wrocław University of Environmental Sciences, Wrocław, Poland. Culture plates and flasks were purchased from Corning Incorporated (Tewksbury, MA, USA) and Nunc Lab-Tek II Chamber Slides were purchased from Thermo Scientific (Rockford, IL, USA). Cells were grown at 37

ºC, in 5% CO_2 (NuAire, Plymouth, MN, USA). Trypsin-EDTA solution (Institute of Immunology and Experimental Therapy, Wrocław) was used to collect the cells.

The peptide 4B8M was synthesized and delivered by one of us (J.Z.), Łódź University of Technology. The synthesis of the peptide was described in US patent [15]. The peptide was initially dissolved in DMSO and subsequently in the culture medium at concentration of 1 mg/ml. The peptide solution was stored frozen at -20 ºC until use. For topical administration the peptide was dissolved by means of 1h stirring in a 50:50 weight mixture of vaseline and paraffin (obtained from a drug store). A microscopic evaluation showed no presence of crystals in the mixture containing up to 1% of the peptide.

Statistics

The calculations and statistical analysis of the data were performed using the licensed Microsoft® Excel XP and STATISTICA® 7 programs for Windows. The statistical significance of differences between mean values were tested by means of variance analysis (ANOVA) for the one-factor classification with post hoc analysis (multi comparisons) by RIR Tukey's test. The evaluation of normality of the variables distribution was performed by W Shapiro-Wilk test, and the evaluation of the homogenous variance assumption by Brown-Forsyth's test. In addition, the occurrence of dependencies between group means and variances were checked. Fulfillment of the above assumptions (normal or symmetric distribution of variables, homogeneity of variance, lack of dependencies between means and variances) is a condition for application of the variance analysis for testing the mean equality hypothesis. The lack of fulfillment of these assumption was a basis for application of non-symmetrical Kruskal-Wallis' test. The significance was set at $P<0.05$. NS – not significant.

Preliminary studies – Materials and Methods

Isolation and proliferation of mouse splenocytes

The spleens were pressed against a plastic screen into 0.83% NH_4Cl solution to lyze erythrocytes (5 min incubation at room temperature). The cells were then washed twice with Hanks' medium, passed through a glass wool column to remove debris, and re-suspended in the culture medium, referred to below as the culture medium, consisting of RPMI-1640, supplemented with 10% FCS, L-glutamine, sodium pyruvate, 2-mecraptoethanol and antibiotics. The cells were then distributed into 96-well flat-bottom tissue culture plates (Nunc) at a density of 2×10^5/100 µl/well. 2.5 µg/ml concanavalin A (Con A) or 2.5 µg/ml

pokeweed mitogen (PWM) were added to induce cell proliferation. The compounds were added to the cultures at doses of 1-100 μg/ml. After a 3-day incubation, the cell proliferation was determined using a colorimetric MTT assay [Hansen et al. 1989]. The results are presented as the mean optical density (OD) at 550 nm with the reference wavelenght of 630 nm ± standard error (SE) from quadruplicate determinations.

Isolation and proliferation of human PBMC

Venous blood from a single donor was withdrawn into heparinized syringes and diluted twice with PBS. PBMC were isolated by centrifugation on Ficoll-uropoline gradient (density 1.077 g/ml) and centrifuged at 800×g for 20 min at 4 ºC. The interphase cells, consisting of lymphocytes (20%) and monocytes (80%) were then washed three times with Hanks' medium and re-suspended in the culture medium at density of 2×10^6 cells/ml. The isolated PBMC were distributed into 96-well flat-bottom plates in 100 μl aliquots (2×10^5 cells/well). PHA was added at a concentration of 5 μg/ml. The compounds were tested at doses 1, 10 and 100 μg/ml. DMSO at appropriate dilutions served as control. After a four-day incubation in a cell culture incubator, the proliferative response of the cells was determined by the colorimetric MTT method [Hansen et al. 1989]. The data are presented as a mean OD value from quadruplicate wells ± SE.

Colorimetric MTT assay for cell growth and kill

The assay was performed according to Hansen et al. 1989. Briefly, 25 μl of MTT (5 mg/ml) stock solution was added per well at the end of cell incubation and the plates were incubated for 3h in a cell culture incubator. Then, 100 μl of the extraction buffer (20% SDS with 50% DMF, pH 4.7) was added. After additional overnight incubation, the optical density (OD) was measured at 550 nm with the reference wavelength of 630 nm (spectrophotometer Dynatech 5000).

Determination of cytokine activities

Human whole blood was diluted 5× with RPMI-1640 medium and distributed to 24-well culture plates in 1 ml aliquots. LPS from *E. coli*, was added to the culture at concentration of 1 μg/ml. The studies compounds were added at concentration of 1, 10 and 100 μg/ml. After overnight incubation the supernatants were harvested and frozen at -20 ºC until cytokine determination. TNF-α and interleukin-6 were measured using bioassays [Espevik and Nissen-Meyer 1986; van Snick et al. 1986].

IL-6 bioassay

IL-6-dependent murine B cell hybridoma (7TD1 line) was incubated (2000 cells/well) in 96-well plates with serial dilutions of supernatants in quadruplicate. Three days later the number of proliferating cells was estimated by the MTT colorimetric method. The detection limit of the assay was about 1.5 pg/ml. One unit of IL-6 activity was defined as an inverse of the supernatant dilution which caused half-maximal (50%) proliferation of the indicator cell line 7TD1.

TNF-α bioassay

The indicator cell line WEHI 164.13 were distributed into 96-flat-bottom plates (Nunc) at a density 2×10^4/well. Serially diluted supernatants (samples) were added (100 μl/well) to the cultures of WEHI 164.13 cells in duplicate. The cells were incubated overnight in the culture medium (RPMI-1640 plus supplements plus FCS) in the presence of actinomycin D (1 μg/ml). The amount of viable cells was measured by MTT colorimetric method. The activity of TNF-α was expressed in pg/ml (10 pg/ml corresponds to one unit of TNF-α). The sensitivity limit for the assay is 2.5 pg/ml. One unit of TNF-α activity is defined as an inverse of supernatant dilution where 50% cell death takes place.

Humoral immune response to SRBC

Secondary humoral immune response *in vitro*: Mice were sensitized i.p. with 0.2 ml of 5% SRBC suspension. After four days spleens from these mice were isolated, splenocyte single cell suspension prepared and suspended in the culture medium at a density of 5×10^6/ml. The cells were distributed to 24-well culture plates in 1 ml aliquots and 0.05 ml of 0.005% SRBC was added as antigen. The compounds were added to the cultures in the beginning of the four-day incubation period at concentration range 10 and 100 μg/ml. The number of antibody-forming cells (AFC) in the cultures were determined using a method of local hemolysis in agar gel according to Mishel and Dutton 1967. The number of viable cells in the suspension was determined in a Bürker hemocytometer in Trypan blue dye. Next, a suspension of SRBC (the antigen) was prepared for the test. To a tube placed in a water bath (45 °C) the following ingredients were added: 0.05 ml of 10× Hanks' concentrate. 0.05 ml of heat inactivated FCS, 0.4 ml of hot (90 °C) 0.6% agarose and (after cooling the solution to the bath temperature) 0.05 ml of 30% SRBC in Hanks' medium. Then, to a tube containing 0.1 ml of complement 0.1 ml of splenocyte suspension was added, warmed for 30 sec in the water bath and mixed with 0.55 ml of the SRBC suspension. The mixture was stirred and poured onto microscopic

glasses. After solidification of the mixture the glasses were placed in humidified boxes at 37 ºC for 3h. After the incubation the plaques (places of lysis) were counted by use of an inverted microscope (10× magnification). The results were shown as a number of AFC per 10^6 viable cells used for the test.

Primary humoral immune response to SRBC *in vivo*: Mice were immunized with 0.2 ml 5% SRBC suspension i.p. and 2h after immunization mice were given i.p. 4B8M peptide (10 or 100 µg/mouse) and other mice received CsA (10 or 100 µg/mouse). Control mice received appropriately diluted DMSO. After four days mice were sacrificed, spleen were isolated and pressed against plastic screens by use of syringe pistons to Hanks' medium to prepare a single cell suspension (4-5×10^4 cells/0.1 ml). The spleen cell suspensions were kept in an ice bath before performance of the test. Next the number of AFC in the spleens was determined using a method of local hemolysis in agar gel as described above.

Delayed type hypersensivity test

The test was performed according to Lagrange et al. 1974. Mice were sensitized subcutaneously with 5 µg ovalbumin (OVA) emulsified in Freund's complete adjuvant in the tail base. After 4 days the mice were challenged with 50 µg OVA in Freund's incomplete adjuvant in the hind footpads. Following next twenty four hours the footpad thickness was measured using a caliper. Control (background response mice) were not sensitized but received the challenging dose of OVA. 4B8M was administered to mice in two 100 µg i.p. doses, 2h before and 24h after the sensitizing dose of antigen. The results were presented as a mean value of antigen-specific increase of footpad thickness and expressed in DTH units (one DTH unit = 10^{-2} cm) ± SE. One DTH unit corresponds to 0.1 mm. Antigen-specific increase means that background (BG) values measured in mice given only the eliciting dose of antigen were subtracted.

Preliminary studies – Results

Effect of the 4B8M peptide on mitogen-induced proliferation of mouse and human lymphocytes

The effect of 4B8M peptide on mitogen-induced proliferation of mouse splenocytes is shown in **Fig. S1AB**. CsA and native CLA served as reference drugs. The inhibitory effects of the compounds were compared with appropriate dilutions of the solvent – DMSO. The results

indicate that the peptide inhibited Con A-induced cell proliferation in a dose-dependent manner (**Fig. S1A**). Its activity was lower as compared to that of native CLA although quite significant at high doses (75% at 75 µg/ml and about 80% at 100 µg/ml). In addition, the peptide strongly inhibited PWM-induced proliferation of splenocytes at 100 µg/ml (**Fig. S1B**).

Fig. S1AB. Effect of the peptide on mitogen-induced proliferation of mouse splenocytes. **A.** Effect of the peptide on ConA-induced splenocyte proliferation; **B.** Effect of the peptide on PWM-induced splenocyte proliferation.

Statistics: **A:** 5 µg/ml: DMSO vs CsA P=0.0001; DMSO vs CLA NS; DMSO vs 4B8M NS; 4B8M vs CsA P=0.0001; 4B8M vs CLA NS (ANOVA); 10 µg/ml: DMSO vs CsA P=0.0001; DMSO vs CLA NS; DMSO vs 4B8M P=0.0472; 4B8M vs CsA P=0.0001; 4B8M vs CLA P=0.0014 (ANOVA); 25 µg/ml: DMSO vs CsA P=0.0001; DMSO vs CLA P=0.0001; DMSO vs 4B8M P=0.0001; 4B8M vs CsA P=0.0001; 4B8M vs CLA P=0.0001 (ANOVA); 50 µg/ml: DMSO vs CsA P=0.0001; DMSO vs CLA P=0.0001; DMSO vs 4B8M P=0.0001; 4B8M vs CsA P=0.0001; 4B8M vs CLA P=0.0001 (ANOVA); 75 µg/ml: DMSO vs CsA P=0.0001; DMSO vs CLA P=0.0001; DMSO vs 4B8M P=0.0001; 4B8M vs CsA P=0.0001; 4B8M vs CLA P=0.0001 (ANOVA); **B:** 1 µg/ml: DMSO vs CLA NS; DMSO vs 4B8M NS; 4B8M vs CLA NS; 10 µg/ml: DMSO vs CLA P=0.0219; DMSO vs 4B8M NS; 4B8M vs CLA P=0.0002; 100 µg/ml: DMSO vs CLA P=0.0001; DMSO vs 4B8M P=0.0001; 4B8M vs CLA NS (ANOVA).

Fig. S1A.

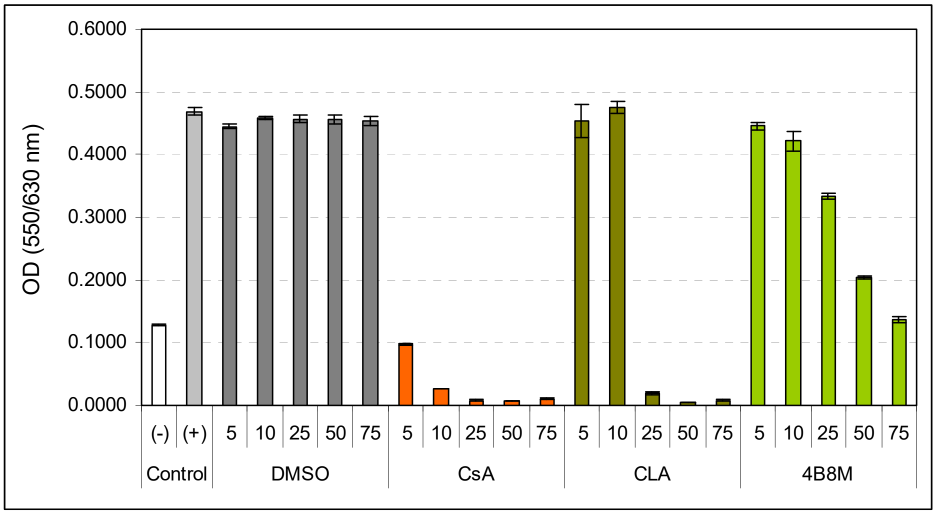

Fig. S1B.

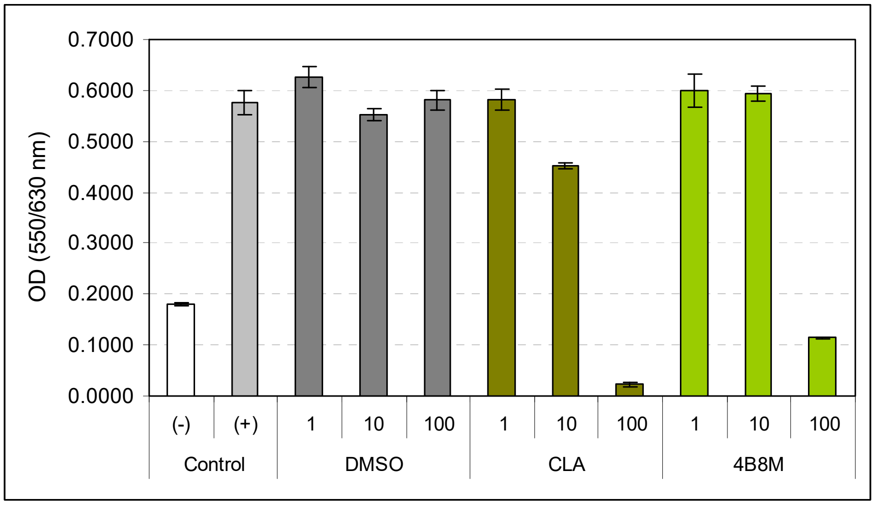

The effect of the peptide on PHA-induced proliferation of human peripheral blood mononuclear cells (PBMC) is shown in **Fig. S2**. In comparison with mouse lymphocytes the inhibitory effect of the peptide was more modest (about 40% suppression at 100 µg/ml).

Fig. S2. Effect of the peptide on PHA-induced proliferation of human PBMC; Control (+) – cells cultured in medium only.

Statistics: DMSO vs 4B8M: 1 µg/ml: NS (P=0.1599); 10 µg/ml: NS (P=0.0788); 100 µg/ml, P=0.0001 (ANOVA).

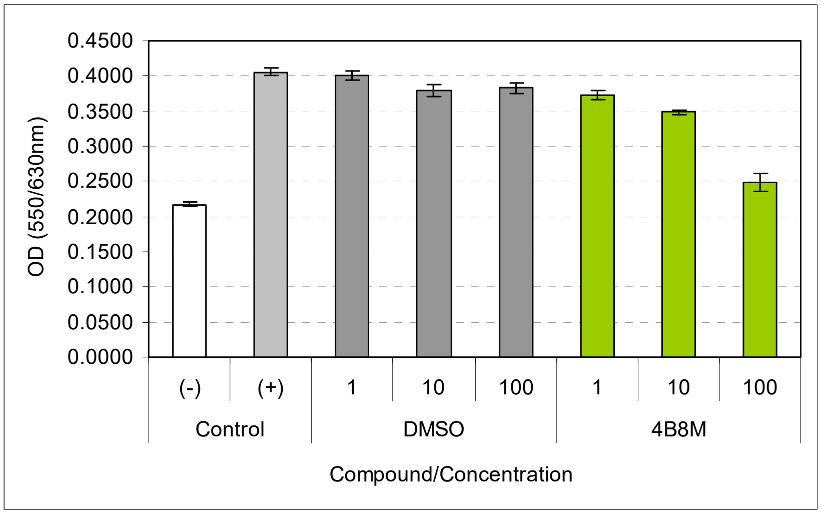

Effect of the peptide on LPS-induced production of TNF-α and IL-6 by human whole blood cell cultures

Effects of 4B8M peptide on LPS-inducible production of TNF-α and IL-6 are presented in **Fig. S3AB**. The peptide inhibited production of TNF-α production by more than 50% at all concentrations. The inhibitory action of CsA was even stronger, particularly at 10 and 100 µg/ml (**Fig. S3A**). 4B8M showed a dose-dependent increase in IL-6 production. Similar, but a stronger increase was observed with CsA at 1 and 10 µg/ml doses but not at 100 µg/ml (**Fig. S3B**).

Fig. S3AB. Effect of the peptide on LPS-inducible TNF-α (**A**) and IL-6 (**B**) production by human whole blood cell cultures; Control (+) – cells cultured in medium only; statistics not applicable.

Fig. S3A.

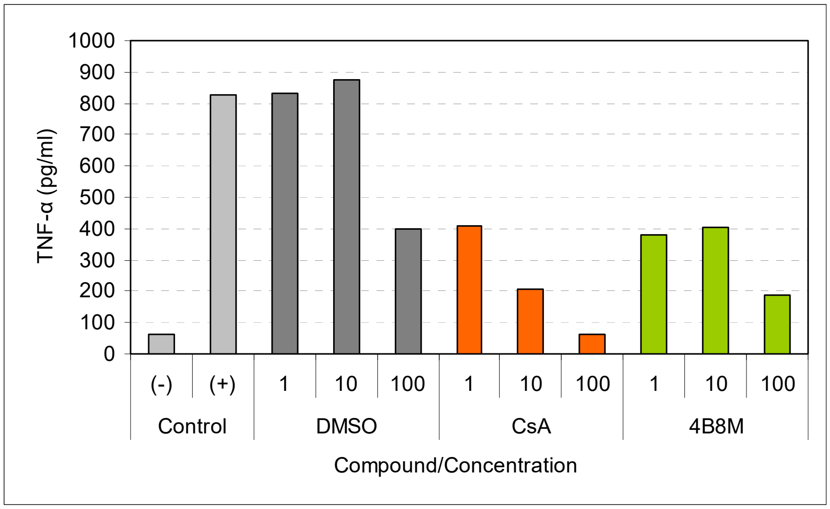

Fig. S3B.

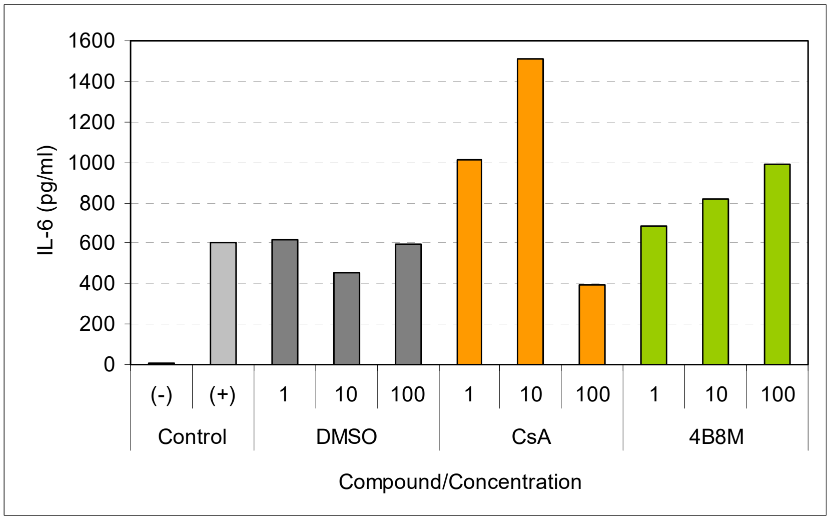

Effect of the 4B8M peptide on the secondary humoral immune response to SRBC *in vitro*

The suppressive effects of 4B8M peptide at 10 and 100 µg/ml doses, are shown in **Fig. S4A**. The results indicate that the suppressive action of the peptide at 100 µg/ml concentration was comparable to those of CsA and CLA. 10 µg/ml concentration of the peptide exerted a weaker suppression as compared to the reference compounds.

Fig. S4A. Effect of the peptide on the secondary humoral immune response mouse splenocytes *in vitro*. Control (-) – splenocytes cultured in medium only (without SRBC); (+) – splenocytes cultured in medium with SRBC.

Statistics: 10 µg/ml: DMSO vs CsA P=0.0001; DMSO vs CLA P=0.0001; DMSO vs 4B8M P=0.0010; CsA vs CLA NS; 4B8M vs CsA NS; 4B8M vs CLA NS (ANOVA); 100 µg/ml: DMSO vs CsA P=0.0001; DMSO vs CLA P=0.0001; DMSO vs 4B8M P=0.0001; CsA vs CLA NS; 4B8M vs CsA NS; 4B8M vs CLA NS (ANOVA).

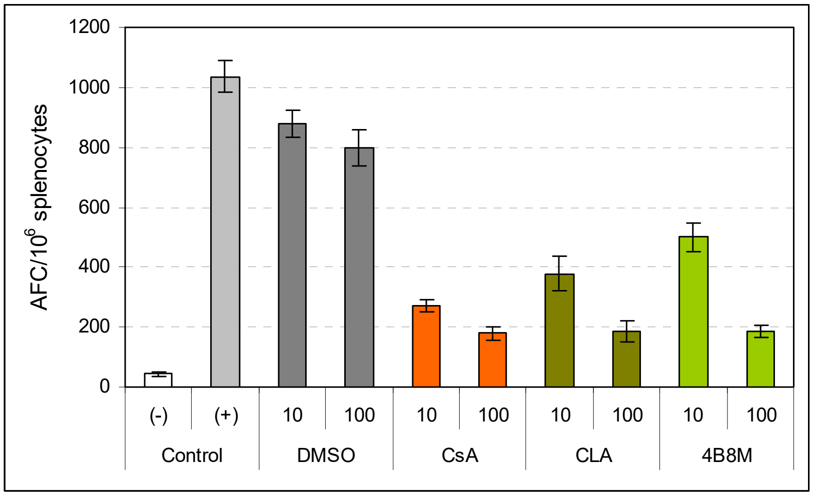

Effect of the peptide on the humoral immune response to SRBC *in vivo*

Mice were immunized with SRBC as described in the Materials and Methods and after 2h were given 10 or 100 µg of the peptide. CsA served as a reference drug. The number of antibody-forming cells to SRBC was measured after four days. The results (**Fig. S4B**) showed that the peptide was strongly inhibitory at both doses. CsA was less effective.

Fig. S4B. Effect of the peptide on the humoral immune response to SRBC *in vivo*.

Statistics: 10 µg/ml: DMSO vs CsA P=0.0034; DMSO vs 4B8M P=0.0001; CsA vs 4B8M P=0.0003; 100 µg/ml: DMSO vs CsA P=0.0001; DMSO vs 4B8M P=0.0001; CsA vs 4B8M NS (P=0.2613) (ANOVA).

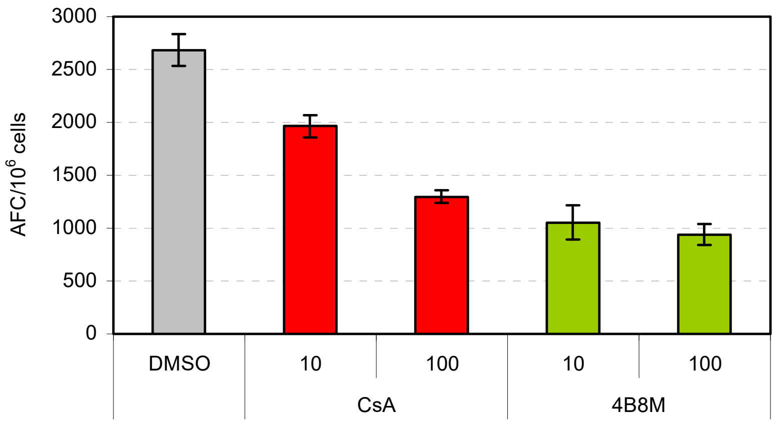

Effect of the peptide on the cellular immune response *in vivo* to ovalbumin

The results showed that 4B8M peptide 100 μg/dose, given in two doses, 2h before and 24h after the immunization of mice, strongly inhibited the delayed type hypersensitivity reaction to OVA. That suppressive action was stronger than those of CLA and CsA (**Fig. S5**).

Fig. S5. Effect of the peptide on the induction phase of the delayed type hypersensitivity to OVA.

Statistics: DMSO *vs* CsA P=0.0001; DMSO *vs* CLA P=0.0001; DMSO *vs* 4B8M P=0.0001; CsA *vs* CLA NS; 4B8M vs CsA P=0.0001; 4B8M vs CLA P=0.0423 (ANOVA).

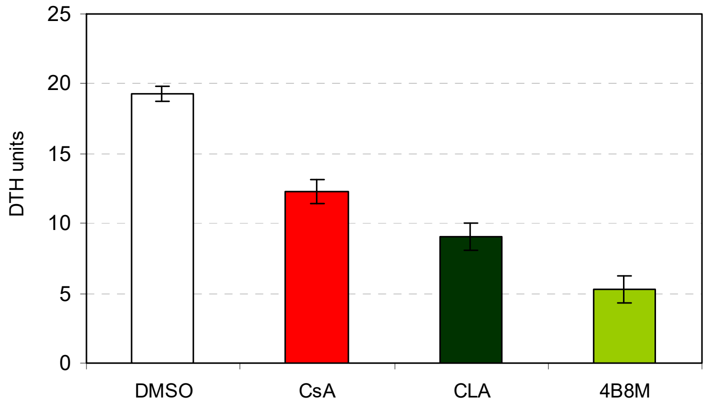

Contact sensitivity to oxazolone – Materials and Methods

Sensitization and elicitation of the contact sensitivity

The test was performed according to Noonan and Halliday, 1978 with some modifications. Mice were shaved on the abdomen (2×2 cm area). On a next day 100 µl of 0.5% oxazolone dissolved in acetone was applied on the skin of abdomen (the sensitizing dose of antigen). After 5 days 50 µl of 1% oxazolone was applied on both sides of the auricles (the eliciting dose of antigen).

Treatment of mice with the preparations

The peptide was applied in a form of 0.1% ointment onto both sides of the auricles (0.1 ml volume), twice during 2h. The reference preparations (Protopic® and Elidel®) were used in a similar way. The auricles of control mice were treated with the creme base. The preparations were applied after 24h following the eliciting dose of antigen, so at the time of the highest manifestation of the cellular inflammatory response. 4B8M peptide was therefore applied as a therapeutic for the contact sensitivity reaction. In another experiment 1% creme Hydrocortisonum® was topically used as a reference drug. 24h after application of the therapeutic agents (48h following elicitation of the reaction) the parameters of the inflammatory reaction were determined.

Measurement of the ear edema

The ear edema (ear thickness) was measured by means of a spring caliper with the accuracy of 0.05 mm (Mitutoyo, Japan) after 48h from application to mice of the eliciting dose of antigen (24h after application of therapeutic preparations). The results were presented as antigen-specific increase of the ear thickness, i.e. BG values were subtracted (background values were measured in mice given only the eliciting dose of antigen).

Cell isolation from lymph nodes

After measurement of the ear edema the mice were sacrificed by cervical dislocation and salivary and mandibular lymph nodes were isolated, placed in PBS and pressed against plastic screen to obtain a single-cell suspension. The cells were washed in PBS in 15 ml tubes by centrifugation at 800×g.

Determination of lymph node cell number and viability

The cells were re-suspended in PBS containing 0.2% trypan blue. The numbers of viable and dead cells were counted in a Bürker hemocytometer in a light microscope. The total number of cells, numbers of viable and dead cells and percentage of viable and dead cells were

shown. The mean values ±SE for each group without subtraction of background values were shown.

Analysis of peripheral blood cell picture

The mice were put asleep in a halothane atmosphere and blood was obtained from the retroorbital plexus. The blood smears were prepared on microscopic glasses. After drying out the smears were stained with Giemsa and May-Grünwald reagents. The smears were subsequently evaluated at 1000× magnification (in immersion oil) in a Nicon ATC 2000 microscope. Up to 100 cells were counted per glass/preparation. The results were presented as a percentage of main cell types in the peripheral blood (mature neutrophils, neutrophil precursors – bands, eosinophils, lymphocytes and monocytes). Mean values for each groups were shown.

Histological analysis

The mice auricles were fixed in 4.0% neutral buffered formalin for 48h, de-hydrated in an alcohol series, cleared in xylene and embedded in paraffin. Paraffin blocks were sliced in a microtome (Microm HM310) into 6 µm sections. The paraffin sections were stained with hematoxylin and eosin (H&E) and with toluidine blue in order to detect mastocytes. The histological and quantitative analysis was conducted in a light microscope Nikon Eclipse 80i with the aid of imagine software NIS-Elements at 400× magnification. The number of neutrophils, macrophages, lymphocytes, mast cells in the connective tissue of auricles in random high-power fields (HPF, area 0.071 mm^2) was estimated. For each studied group 25 determinations of the above mentioned cell types were performed. Mean values for each group without subtraction of the background values were shown.

Therapeutic effect of 4B8M peptide on the reaction of contact sensitivity to oxazolone

Effect of the peptide on the number of lymphocytes in the draining lymph nodes

4B8M and Elidel® lowered the total number of lymph node cells to the level observed in the BG group (**Fig. S6A**). Interestingly, Protopic® elevated the number of cells in the draining lymph nodes in relation to not treated control mice.

Fig. S6A. The number of cells in the draining lymph nodes (the total number of viable and dead cells).

Statistics: BG *vs* Control P=0.0289; BG *vs* 4B8M NS; BG *vs* Protopic® NS; BG *vs* Elidel® NS; Control *vs* 4B8M P=0.0024; Control *vs* Protopic® NS; Control *vs* Elidel® P=0.0009; 4B8M *vs* Protopic® NS; 4B8M *vs* Elidel® NS (test of K-W).

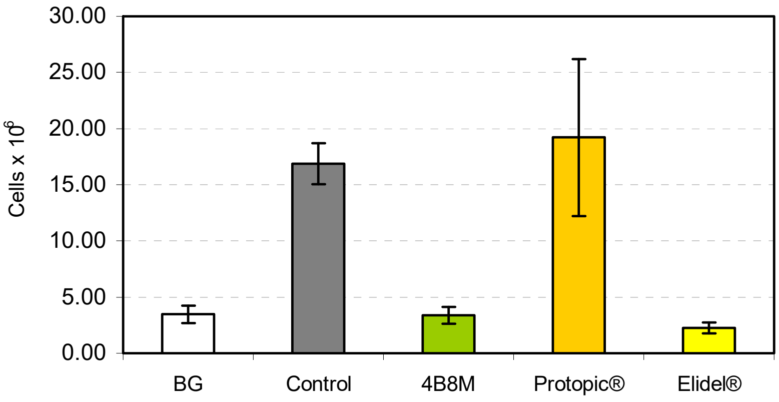

<u>Effect of the peptide of number and percentage of viable and dead lymphocytes in the draining lymph nodes</u>

The number of dead cells in the draining lymph nodes after application of Protopic® increased significantly, whereas 4B8M and Elidel® decreased the number of dead cells as compared to non-treated control group (**Fig. S6B**).

Fig. S6B. The numbers of viable and dead cells in the draining lymph nodes.

Statistics: <u>viable cells</u>: BG vs Control NS; BG vs 4B8M NS; BG vs Protopic® NS; BG vs Elidel® NS; Control vs 4B8M P=0.0106; Control vs Protopic® P=0.0001; Control vs Elidel® P=0.0011; 4B8M vs Protopic® NS; 4B8M vs Elidel® NS (test of K-W); <u>dead cells</u>: BG vs Control P=0.0271; BG vs 4B8M NS; BG vs Protopic® P=0.0003; BG vs Elidel® NS; Control vs 4B8M P=0.0058; Control vs Protopic® NS; Control vs Elidel® NS; 4B8M vs Protopic® P=0.0001; 4B8M vs Elidel® NS (test of K-W).

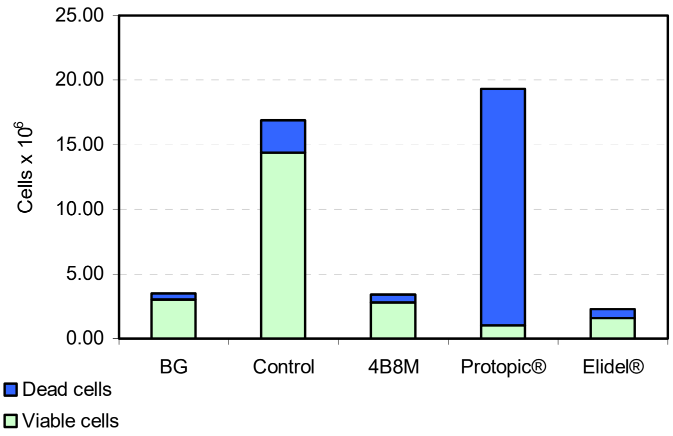

The analysis of the percentage of viable and dead dells in the draining lymph nodes showed that the peptide did not increase the content of dead cells in relation to the untreated control (**Fig. S6C**). However, a 2× increase was observed with application of Elidel®. Protopic®, in turn, increased the % of dead cells up to 95%.

Fig. S6C. The percentage of viable and dead cells in the draining lymph nodes.

Statistics: viable cells: BG vs Control NS; BG vs 4B8M NS; BG vs Protopic® NS; BG vs Elidel® NS; Control vs 4B8M P=0.0106; Control vs Protopic® P=0.0001; Control vs Elidel® P=0.0011; 4B8M vs Protopic® NS; 4B8M vs Elidel® NS (test of K-W); dead cells: BG vs Control P=0.0271; BG vs 4B8M NS; BG vs Protopic® P=0.0003; BG vs Elidel® NS; Control vs 4B8M P=0.0058; Control vs Protopic® NS; Control vs Elidel® NS; 4B8M vs Protopic® P=0.0001; 4B8M vs Elidel® NS (test of K-W).

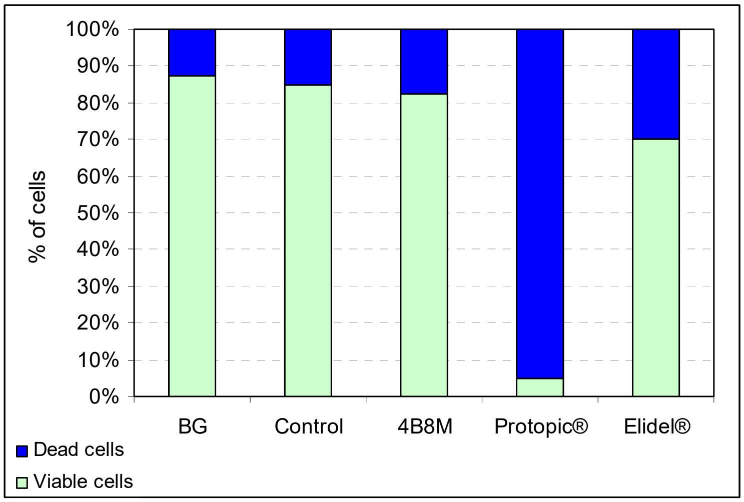

Therapeutic effect of 4B8M in comparison with 1% Hydrocortisonum® (HC)

The aim of the next experiment was to topically apply the peptide (0.1% ointment) as a therapeutic agent preventing the contact sensitivity reaction in comparison with Hydrocortisonum® 1% creme as a reference drug. **Fig. S7** shows that both compounds inhibited the ear swelling to a similar degree. 4B8M peptide and HC were also very effective in reducing draining lymph node cell number (**Fig. S8**). On the other hand the treatment with hydrocortisone led to a significant increase of the neutrophil (bands and segments) content in the peripheral blood, and the blood cell composition in 4B8M group was comparable to BG mice (**Fig. S9**).

Fig. S7. The antigen-specific increase of the ear thickness.

Statistics: Control vs 4B8M P=0.0001; Control vs HC P=0.0001; 4B8M vs HC NS (ANOVA).

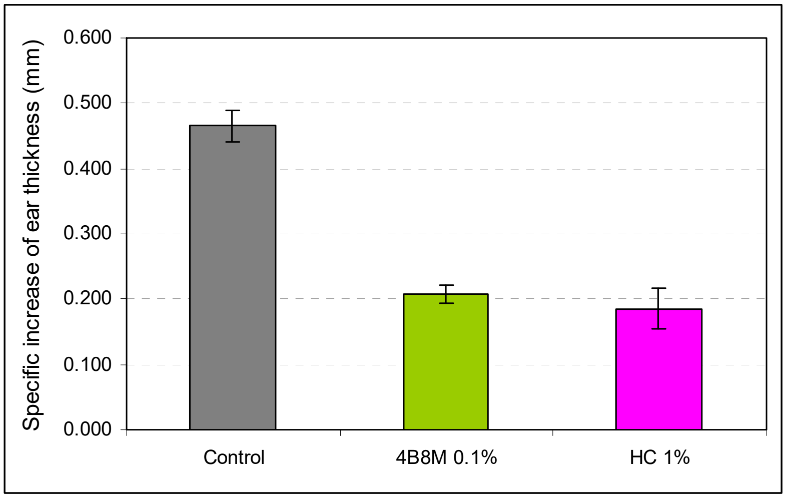

Fig. S8. The number of cells in the draining lymph nodes.

Statistics: BG vs Control P=0.0001; Control vs 4B8M P=0.0001; Control vs HC P=0.0001; 4B8M vs HC NS (ANOVA).

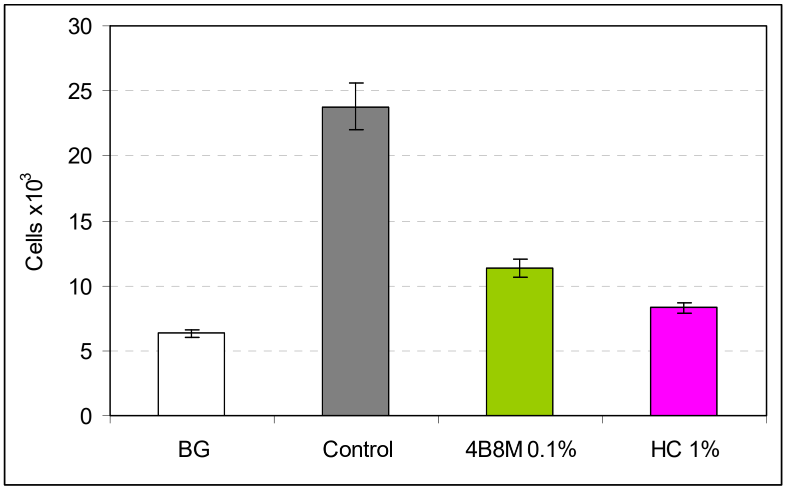

Fig. S9. The peripheral blood cell composition.

Statistics: Bands (B): BG *vs* Control NS; Control *vs* 4B8M NS; Control *vs* HC P=0.0470; 4B8M *vs* HC NS (ANOVA); Segments (S): BG *vs* Control P=0.0490; Control *vs* 4B8M P=0.0023; Control *vs* HC NS; 4B8M *vs* HC P=0.0001 (ANOVA); Eosinophils (E): BG *vs* Control NS; Control *vs* 4B8M P=0.0002; Control *vs* HC NS; 4B8M *vs* HC P=0.0026 (ANOVA); Lymphocytes (L): BG *vs* Control P=0.0110; Control *vs* 4B8M P=0.0070; Control *vs* HC NS; 4B8M *vs* HC P=0.0002 (ANOVA); Monocytes (Mono): BG *vs* Control P=0.0110; Control *vs* 4B8M P=0.0002; Control *vs* HC NS; 4B8M *vs* HC P=0.0026 (ANOVA).

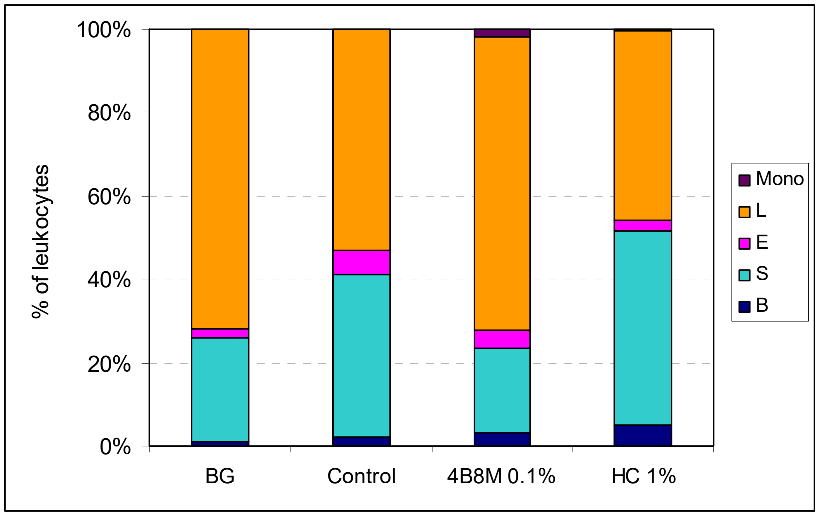

The normalizing effects of the peptide on the inflammatory skin histological changes induced by oxazolone, are presented in **Fig. S10** and **Fig. S11**.

Fig. S10AB. The histology of the auricle of BG mice. **A.** H&E staining, **B.** toluidine blue staining. The histological picture of BG mice auricle presents a normal structure. In the central part of the auricle the elastic cartilage is seen. In a close proximity of the unchanged epidermis the sebaceous glands are situated. In the perivascular zone single lymphocytes and macrophages can be found. Single neutrophils are restricted mainly to the interior of blood vessels. Mastocytes occur in a condensed form within the connective tissue and around blood vessels, 400× magnification.

Abbreviations: Ep – epidermis; EC – elastic cartilage; MT – muscle tissue; Mast – mastocyte; Ne – neutrophil; Mø – macrophage; L – lymphocyte; Inf – inflammatory infiltrate

Fig. S10A.

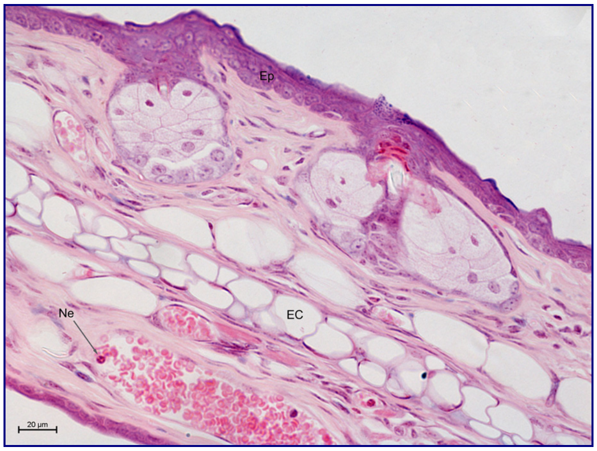

Fig. S10B.

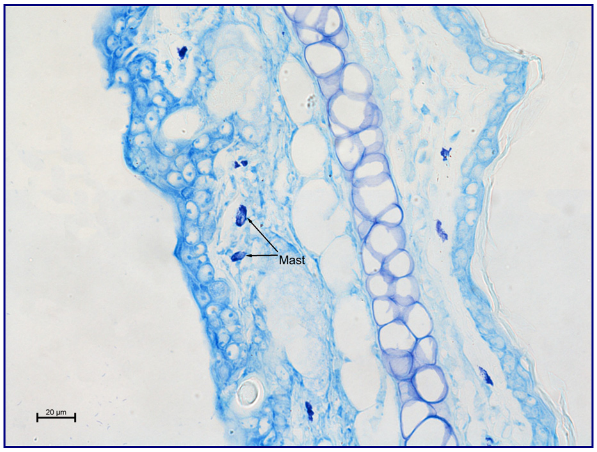

Fig. S10CD. The histological picture of auricles after 48h following elicitation of the contact sensitivity reaction in control, untreated mice. **C.** H&E staining, **D.** toluidine blue staining. The picture presents extensive inflammatory cell infiltrations, composed mainly of neutrophils and to a lesser degree of macrophages and lymphocytes. In some places, in the zone adjacent to perichondrium, a loosening of the connective, fibrous tissue was observed, probably associated with perivascular swelling, 400× magnification.

Fig. S10C.

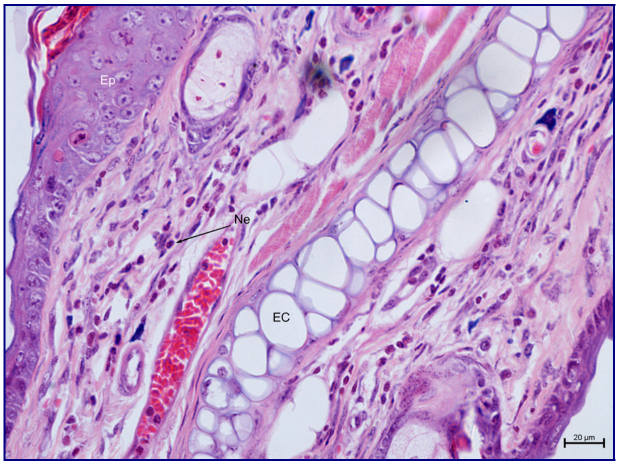

Fig. S10D.

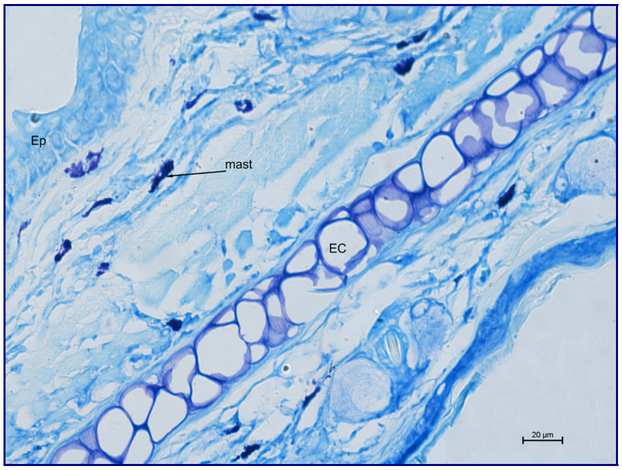

Fig. S10EF. The histological picture of auricles after 48h following elicitation of the contact sensitivity reaction in 4B8M-treated mice. **E.** H&E staining, **F.** toluidine blue staining. In these mice the histology of auricles resembled that of background mice. Single neutrophils, macrophages and lymphocytes were spotted. The dominant types of cells in the connective tissue are fibroblasts and fibrocytes that proves occurrence of an increased fibrogenesis as a repair process following inflammation. Mastocytes are less numerous, similarly as it is observed in the skin of BG mice, 400× magnification.

Fig. S10E.

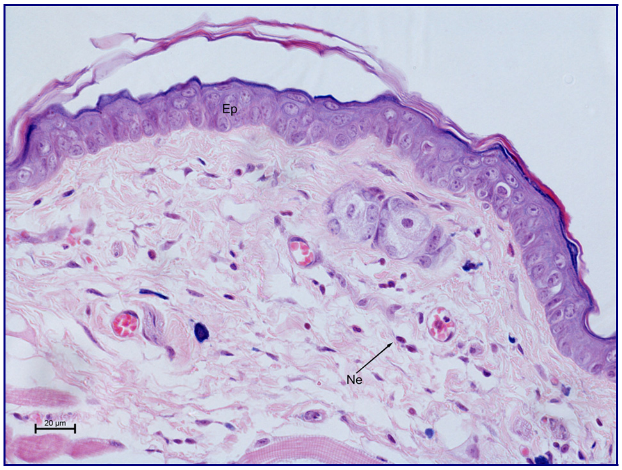

Fig. S10F.

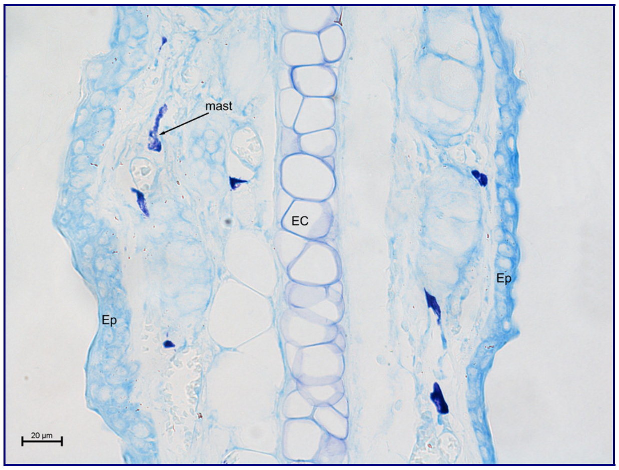

Fig. S10GH. The histological picture of auricles after 48h following elicitation of the contact sensitivity reaction in Protopic®-treated mice. **G.** H&E staining, **H.** toluidine blue staining. The auricles of mice treated with Protopic® are significantly inflamed. The inflammatory infiltrations are composed mainly of neutrophils, macrophages and lymphocytes. The inflammatory cells are predominantly concentrated around blood vessels. The zone of infiltration also includes the connective tissue adjacent to perichondrium. The infiltrating cells were observed in a lesser degree around skin glands and in the subepithelial connective tissue. In the histochemical studies numerous mastocytes were found, at different functional stages, grouped around blood vessels. The intensity of mastocytes concentration was proportional to the intensity of cell infiltration, 400× magnification.

Fig. S10G.

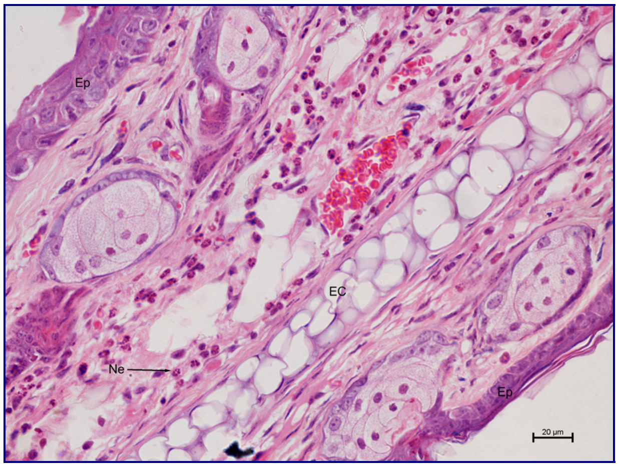

Fig. S10H.

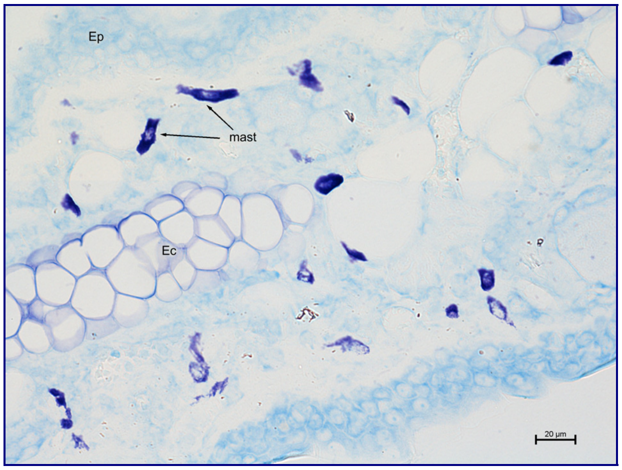

Fig. S10IJ. The histological picture of auricles after 48h following elicitation of the contact sensitivity reaction in Elidel®-treated mice. **I.** H&E staining, **J.** toluidine blue staining. In the auricle section of mice treated with Elidel® a distinct diminution of the inflammatory process was registered. This effect regarded both the perivascular zone as well as the subepithelial connective tissue. A reduced number of neutrophils in the infiltration was observed. The inflamed zone was distinctly diminished, as well as the number of inflammatory cells, 400× magnification.

Fig. S10I.

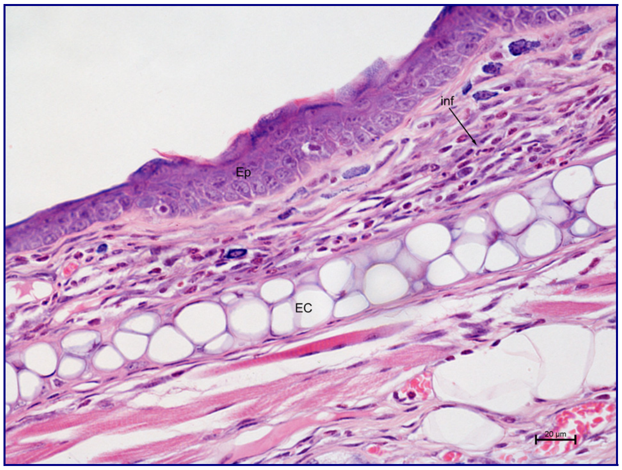

Fig. S10J.

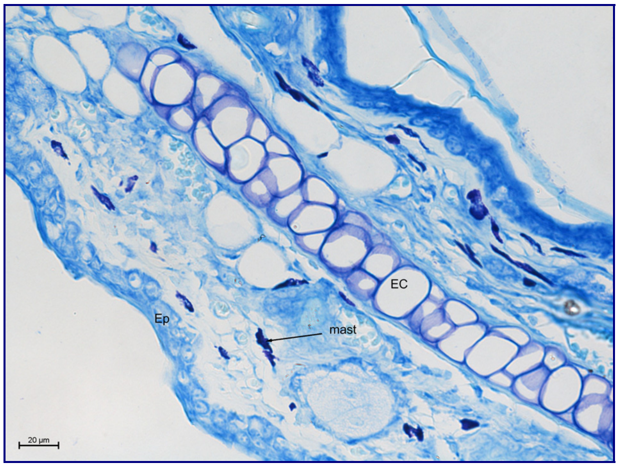

The results of quantitative analysis of the inflammatory cell types in high-power field are presented in **Fig. S11**. The histological examinations revealed that the cellular composition at 24h after treatment with 4B8M peptide resembled that of naive mice. In contrast, Protopic® did not change the content of neutrophils and the number of macrophages and lymphocytes exceeded those in the control mice. The treatment with Elidel® led to about 50% reduction in neutrophil numbers, however, more mastocytes could be found in that area.

Fig. S11. Morphometric analysis of the auricle tissue.

Statistics: Neutrophils (Ne): BG vs Control P=0.0001; BG vs 4B8M NS; BG vs Protopic® P=0.0001; BG vs Elidel® p=0.0001; Control vs 4B8M P=0.0001; Control vs Protopic® NS; Control vs Elidel® P=0.0003; 4B8M vs Protopic® P=0.0001; 4B8M vs Elidel® P=0.0185 (test of K-W); macrophages (MØ): BG vs Control P=0.0223; BG vs 4B8M P=0.0166; BG vs Protopic® P=0.0001; BG vs Elidel® P=0.0008; Control vs 4B8M NS; Control vs Protopic® P=0.0001; Control vs Elidel® NS; 4B8M vs Protopic® P=0.0001; 4B8M vs Elidel® NS (test of K-W); lymphocytes (L): BG vs Control NS; BG vs 4B8M NS; BG vs Protopic® P=0.0001; BG vs

Elidel® P=0.0070; Control vs 4B8M NS; Control vs Protopic® P=0.0001; Control vs Elidel® NS; 4B8M vs Protopic® P=0.0001; 4B8M vs Elidel® NS (ANOVA); mastocytes (Mast): BG vs Control NS; BG vs 4B8M NS; BG vs Protopic® P=0.0001; BG vs Elidel® P=0.0001; Control vs 4B8M NS; Control vs Protopic® NS; Control vs Elidel® NS; 4B8M vs Protopic® P=0.0060; 4B8M vs Elidel® P=0.0004 (test of K-W).

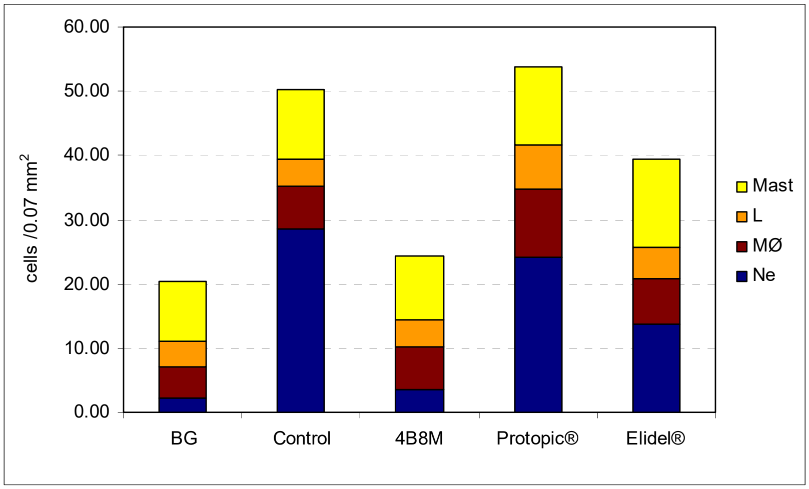

Skin inflammation to toluene diisocyanate – Materials and Methods

The test was performed according to Yamamoto et al. 2006, with minor modifications. The mice were shaved on the abdomen (2×2 cm) and after 24h 100 µl of 3% TDI in acetone for 3 subsequent days were applied (the sensitizing dose of antigen). After 14 days the antigen-specific skin reaction was elicited by five applications of 20 µl of 0.3% TDI (the eliciting dose of antigen) on both sides of the auricles in 3-day intervals: days 14, 17, 20, 23 and 27. 5h and 24h following the last elicitation of the reaction (27th and 28th days of the test, respectively) the parameters of the inflammatory reaction were determined.

Application of the peptide and the reference compounds

4B8M peptide was applied in a form of 0.1% ointment, topically, on both sides of the auricles (total volume of 100 µl), 1h after each elicitation of the reaction by TDI (days: 14, 17, 20, 23 and 27 of the test). The reference drugs (Protopic® and Elidel®) were applied in the same manner.

Determination of leukocyte number

After 5h (27th day of the test) and after 24h (28th day of the test) following the last elicitation of the reaction, the mice were subjected to halothane anesthesia and bled from the retroorbital plexus, followed by sacrifice by cervical dislocation. The number of leukocytes was determined after 20× dilution of blood in Türk's solution and enumeration of cells in a Bürker

camera. The results were presented as the number of cells per 1 µl of blood as mean values ±SE.

Determination of cell blood composition

After 24h (27th day of the test) mice were bled and blood smears were made on microscopic glasses, the preparations were dried and stained with Giemsa and May-Grünwald reagents. The blood picture was evaluated in a light microscope at 1000× magnification (in immersion oil). Up to 100 cells was counted per preparation. The results were shown as a percentage (mean values of respective cell types: mature neutrophils, band forms of neutrophils, eosinophils, lymphocytes and monocytes).

Determination of cell numbers in the draining lymph nodes

After sacrifice of mice (24h following the last elicitation of the reaction – 28th day of the test) the superficial parotid, mandibular and accessory mandibular lymph nodes were isolated, homogenized by pressing against a stainless screen into PBS, washed 2× with PBS and re-suspended in PBS containing 0.2% Trypan blue. The total and nonviable cell numbers were counted using a light microscope and Bürker's hemocyctometer.

Blood vessel permeability test

The test was performed according to Katayama et al, 1978 with some modifications. 5h following the last elicitation of the reaction (27th day of the test) the mice were given 1 mg of Evens blue in 0.2 ml of 0.9% NaCl, intravenously. After 30 min the mice were sacrificed, the auricles were cut off, weighed and immersed in 50 µl of 1 M KOH for 18h at 37 ºC. The dye was extracted from the ears using 450 µl of 0.2 M phosphoric acid and acetone (5:13 ratio). The samples were centrifuged at 3,000 rpm for 15 min. OD of the supernatants were measured at 550/630 nm. The amount of Evens blue (µg/ml) was determined based on a standard curve. The results were presented as the amount of Evans blue per 100 mg of wet tissue. Mice treated only with the eliciting dose of antigen served as a BG control.

Histological analysis and statistics as described above.

Therapeutic effect of 4B8M peptide on skin inflammation to toluene diisocyanate

Effect of the peptide on peripheral blood leukocyte number

Effects of the 4B8M peptide and the reference compounds on the number of circulating leukocytes was shown in **Fig. S12**. Blood for analysis derived from mice 5h after last (5th) application of the eliciting dose of antigen. A complete return to the BG number of leukocytes in mice treated with the 4B8M peptide was indicated, and a lesser efficacy for Elidel® and Protopic® (not significant changes).

Fig. S12. Leukocyte numbers in the circulating blood (5h measurement).

Statistics: BG vs Control NS; Control vs 4B8M P=0.0063; Control vs Protopic® NS; Control vs Elidel® NS; 4B8M vs Protopic® NS; 4B8M vs Elidel® NS (ANOVA of K-W).

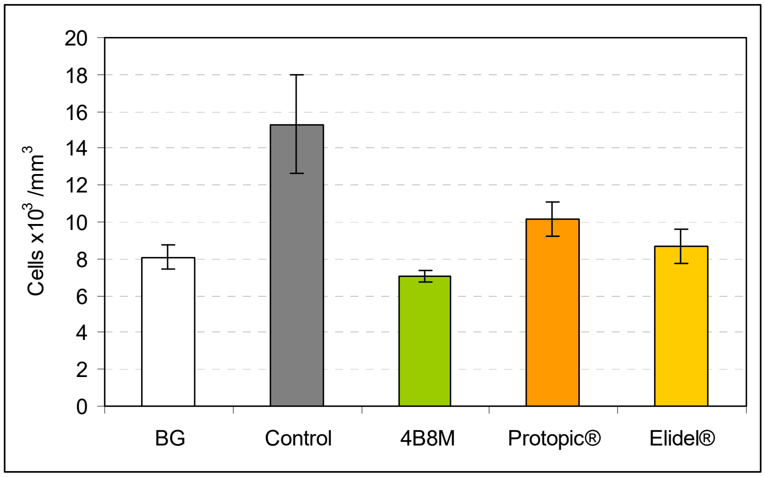

Effect of the peptide on the blood cell composition

The changes in blood picture of mice (24h measurement from the last application of the eliciting dose of antigen) were shown in **Fig. S13**. In the blood picture of inflammed mice not treated with the compounds (Control group) an increased level of eosinophils (to 7.2%) was noted as compared to 2.2% in BG group. The application of 4B8M reduced the % of eosinophils to 1.3%. Protopic® and Elidel® also decreased the eosinophil content although to lesser degree than 4B8M (to 2.2 and 2.0%, respectively).

Fig. S13. The peripheral blood picture (24h measurement).

Statistics: bands (B): BG vs Control NS; Control vs 4B8M NS; Control vs Protopic® NS; Control vs Elidel® NS; 4B8M vs Protopic® NS; 4B8M vs Elidel® NS; segments (S): BG vs Control NS; Control vs 4B8M NS; Control vs Protopic® NS; Control vs Elidel® NS; 4B8M vs Protopic® NS; 4B8M vs Elidel® NS; eosinophils (Eo): BG vs Control P=0.0001; Control vs 4B8M P=0.0001; Control vs Protopic® P=0.0001; Control vs Elidel® P=0.0001; 4B8M vs Protopic® NS; 4B8M vs Elidel® NS; lymphocytes (L): BG vs Control NS; Control vs 4B8M P=0.0255; Control vs Protopic® NS; Control vs Elidel® NS; 4B8M vs Protopic® NS; 4B8M vs Elidel® NS; monocytes (Mono) (ANOVA).

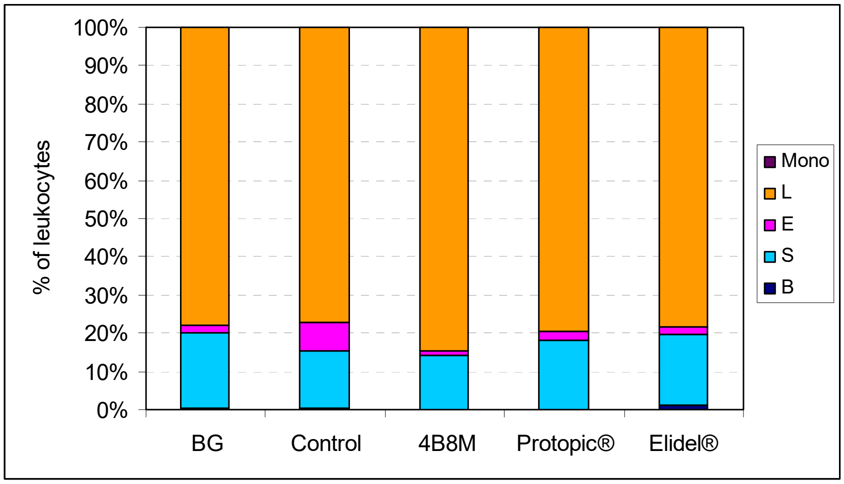

Effect of the peptide on permeability of capillary blood vessels

The effects of the compounds on the permeability of skin capillary vessels of the auricles (5h measurement from the last application of the eliciting dose of antigen) were shown in **Fig. S14**. The results indicate that all compounds were highly effective in suppression of the blood vessel permeability, measured as amount of Evans blue dye penetrating the tissue.

Fig. S14. The effects of the peptide on permeability of capillary blood vessels (5h measurement).

Statistics: BG vs Control P=0.0035; Control vs 4B8M P=0.0147; Control vs Protopic® P=0.0147; Control vs Elidel® P=0.0308; 4B8M vs Protopic® NS; 4B8M vs Elidel® NS (ANOVA of K-W).

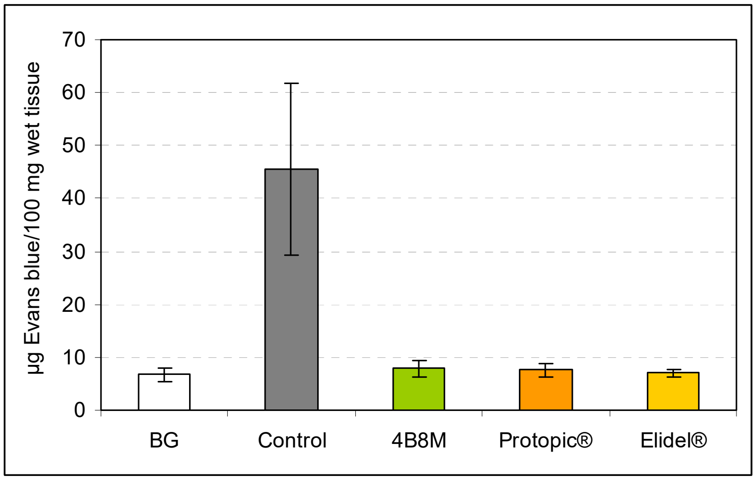

Effects of the 4B8M peptide on histological picture of auricles

The effects of the peptide and reference compounds on the histological changes of auricles (5h measurement from the last application of the eliciting dose of antigen) are presented in photographs (**Fig. S15**) and figures **S16** and **S17**.

Fig. S15AB. The histology of the normal structure of the BG mice. **A.** H&E staining, **B.** toluidine blue staining. The epidermis is thin with distinctly visible basal and spinous layers and weakly separated granular, lucid and corneal layers. In the perivascular zone single lymphocytes and macrophages can be found. Single neutrophils were restricted mainly to the interior of blood vessels. The mastocytes occurred in a condensed form within the subepithelial connective tissue and around blood vessels.

Fig. S15A.

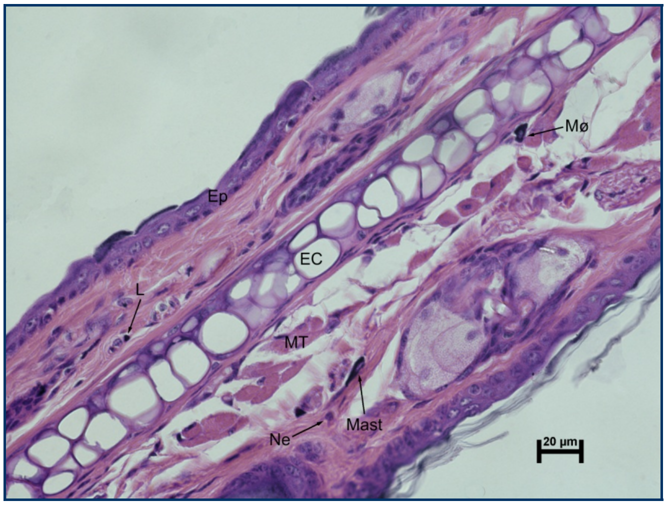

Fig. S15B.

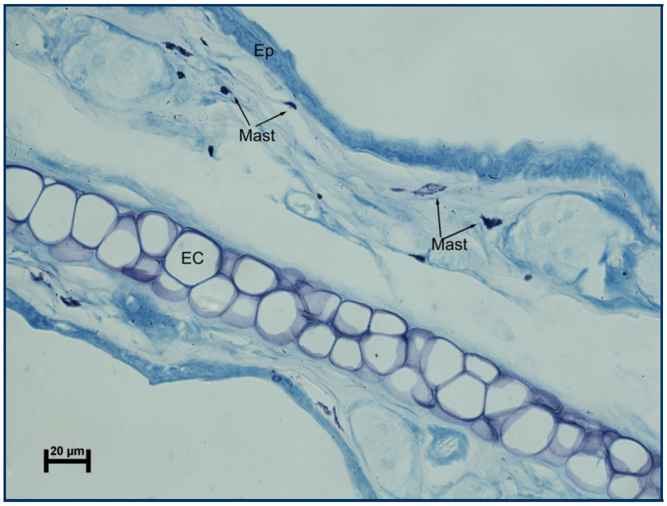

Fig. S15CD. The histological picture of auricles of control, untreated mice. **C.** H&E staining, **D.** toluidine blue staining. In control mice the tissue presented an inflammation. The histometric analysis revealed not significant thickening of the epidermis in relation to control, background mice. The cell infiltrates observed in the dermis consisted mainly of neutrophils and mastocytes and were localized in the perivascular and subepithelial zones of dermis. The mastocytes were in the degranulation state. In some areas a loosening of the connective, fibrous tissue was observed indicating edema of the connective tissue.

Fig. S15C.

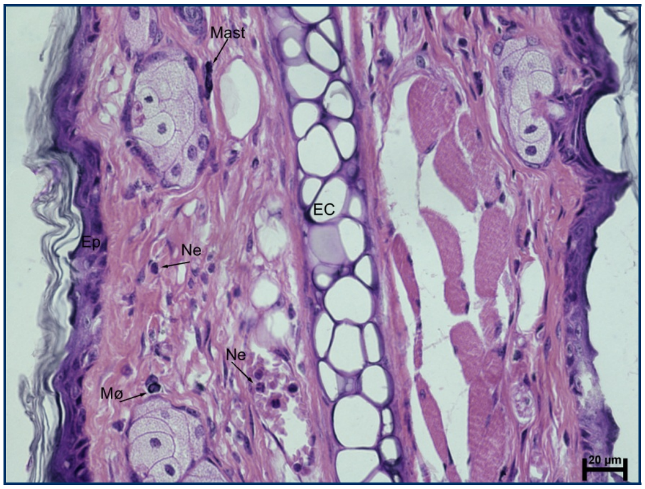

Fig. S15D.

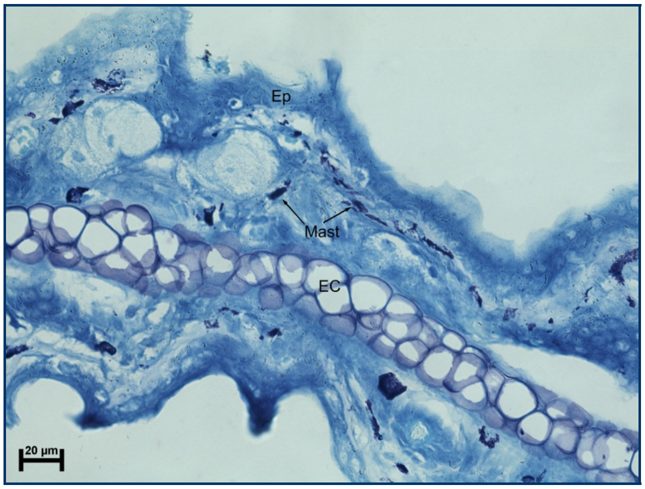

Fig. S15EF. The histological picture of auricles in 4B8M-treated mice. **E.** H&E staining, **F.** toluidine blue staining. In mice treated with the peptide a stabilization of the inflammatory process was observed. The thickness of the epidermis in this case was comparable to that

observed in the non sensitized mice. Some cell infiltrations were observed in the perivascular zone, albeit less intensive as compared with control, untreated mice. The morphometric analysis revealed a decrease of the neutrophil and mastocyte numbers within the inflammatory infiltrates. The granules in mastocytes were in a state of formation and condensation.

Fig. S15E.

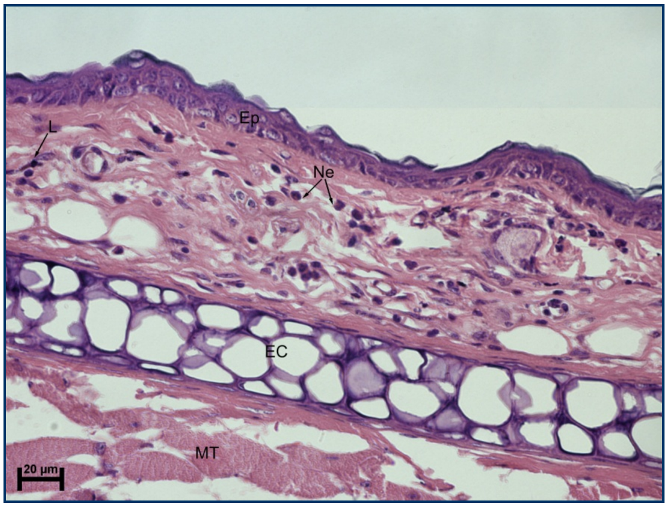

Fig. S15F.

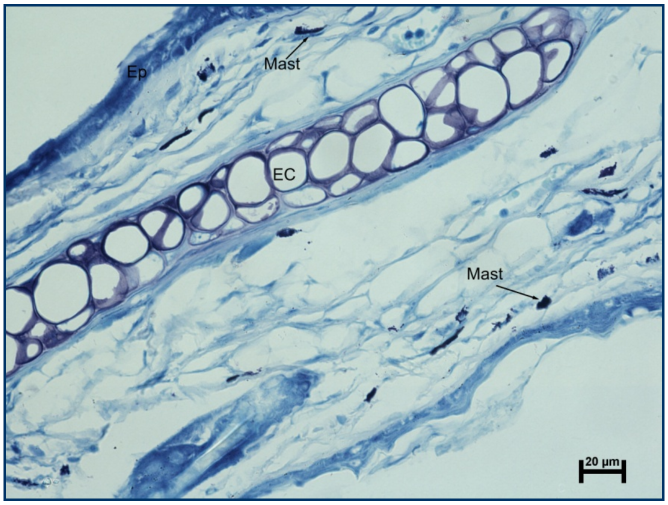

Fig. S15GH. The histological picture of auricles in Protopic®-treated mice. **G.** H&E staining, **H.** toluidine blue staining. In mice treated with Protopic® a stabilization of the inflammation was observed as assessed by diminished numbers of mastocytes and neutrophils in the connective tissue. The mastocytes contained also less numerous granules. The dermis

displayed a normal arrangement of collagen fibers, and a stabilized number of fibrocytes and fibroblasts. The epidermis in the analyzed group was thin, without signs of intensified cornification.

Fig. S15G.

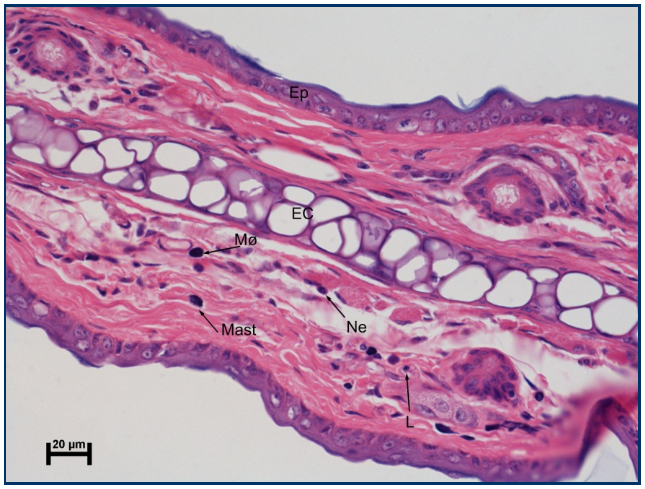

Fig. S15H.

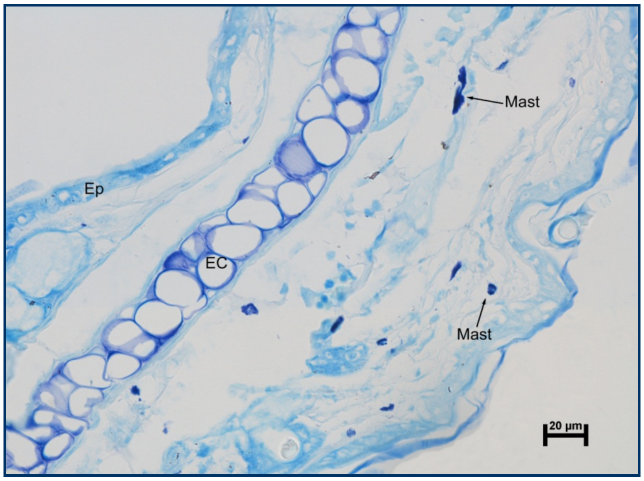

Fig. S15IJ. The histological picture of auricles in Elidel®-treated mice. **I.** H&E staining, **J.** toluidine blue staining. The auricles of mice treated with Elidel® presented a strong inflammation. An increased proliferation of the epidermal basal layer, significantly thicker spinous layer may indicate akanthosis but thicker corneal layer proves about hyperkeratosis of epidermis. In the lower parts of epidermis focal spongiosis (enlarged spaces between keratinocytes) was registered and infiltration of single lymphocytes was revealed. In the dermis the inflammatory changes were more strongly pronounced. Vast infiltrations,

consisting mainly of mastocytes and neutrophils, and to a lesser degree of macrophages and lymphocytes, were observed. Toluidine blue staining showed a significant increase of the mastocyte numbers as compared to other groups. Mastocytes formed clusters around blood vessels, in the zone of connective, subepithelial tissue and around glands. The cells were packed with granules and the majority of cells were in a degranulation phase. Also, a loosening of the architecture of the collagen fibers was noted which could explain the swelling of the connective tissue.

Fig. S15I.

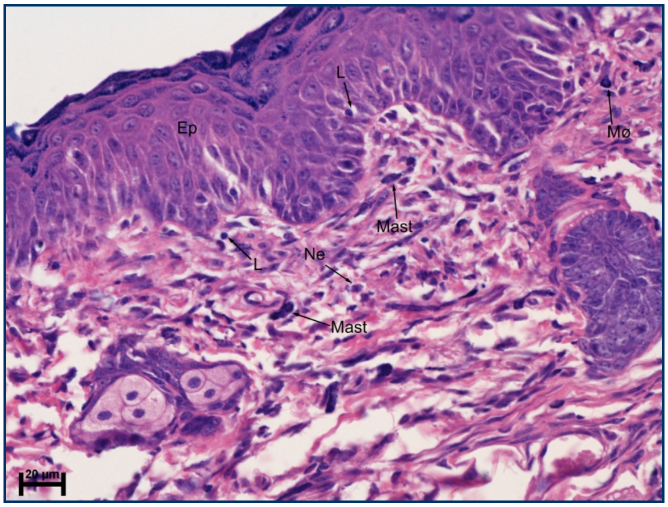

Fig. S15J.

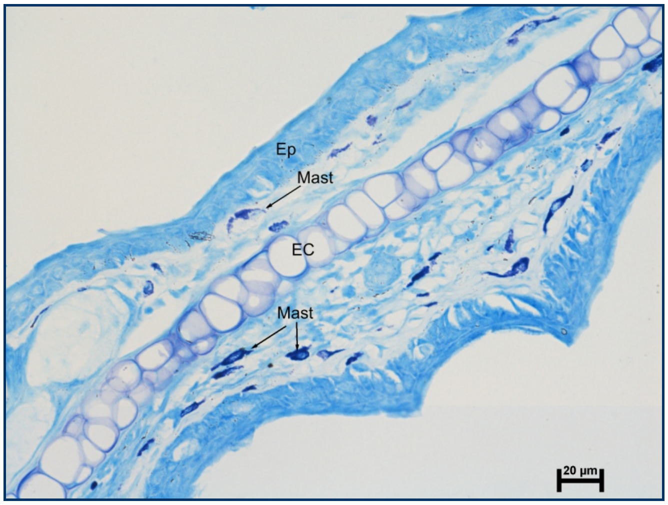

In **Fig. S16** the cell composition of the inflamed auricle tissue (5h measurement from the last application of the eliciting dose of antigen) is presented. In control mice, not treated with the

compounds, a significant increase of the neutrophil number (infiltration) and mastocytes, as compared to the normal picture, was observed. A diminution of the neutrophil infiltration, as well as mastocytes, is best seen upon application of Protopic®. 4B8M peptide showed somewhat weaker action. Elidel®, in turn, significantly increased infiltration of mastocytes, macrophages and lymphocytes. **Fig. S17** illustrates changes regarding the thickness of the epidermis (5h measurement). 4B8M peptide and Protopic® exhibited comparable action, whereas Elidel® caused about 2-fold increase of the epidermis.

Fig. S16. Histological analysis of the auricle tissue (number of cells of a given type, morphometric analysis; 5h measurement).

Statistics: neutrophils (Ne): BG vs Control P=0.0000; Control vs 4B8M P=0.0151; Control vs Protopic® P=0.0000; Control vs Elidel® NS; 4B8M vs Protopic® NS; 4B8M vs Elidel® P=0.0031; macrophages (MØ): BG vs Control P=0.0144; Control vs 4B8M NS; Control vs Protopic® NS; Control vs Elidel® P=0.0255; 4B8M vs Protopic® NS; 4B8M vs Elidel® P=0.0031; lymphocytes (L): BG vs Control NS; Control vs 4B8M NS; Control vs Protopic® NS; Control vs Elidel® P=0.0023; 4B8M vs Protopic® NS; 4B8M vs Elidel® P=0.0001; mastocytes (Mast): BG vs Control P=0.0000; Control vs 4B8M NS; Control vs Protopic® P=0.0000; Control vs Elidel® NS; 4B8M vs Protopic® NS; 4B8M vs Elidel® P=0.0000 (ANOVA of K-W).

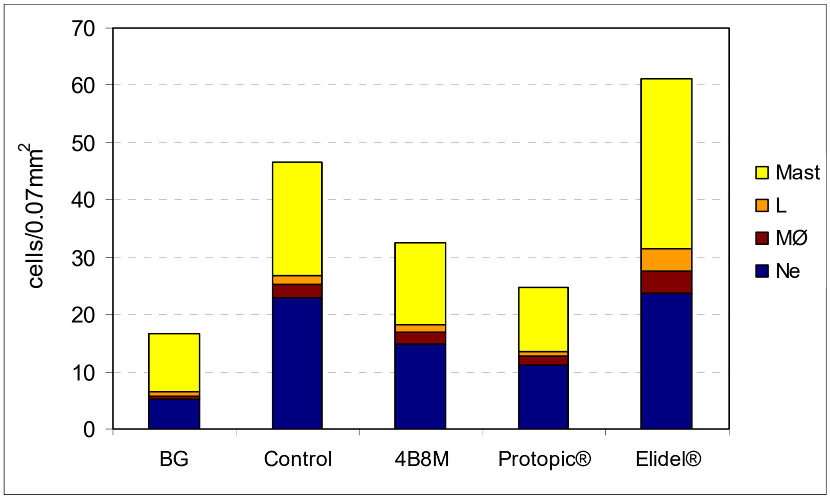

Fig. S17. Thickness of the epidermis (5h measurement).

Statistics: BG vs Control P=0.0004; Control vs 4B8M P=0.0355; Control vs Protopic® P=0.0164; Control vs Elidel® NS; 4B8M vs Protopic® NS; 4B8M vs Elidel® P=0.0000 (ANOVA of K-W).

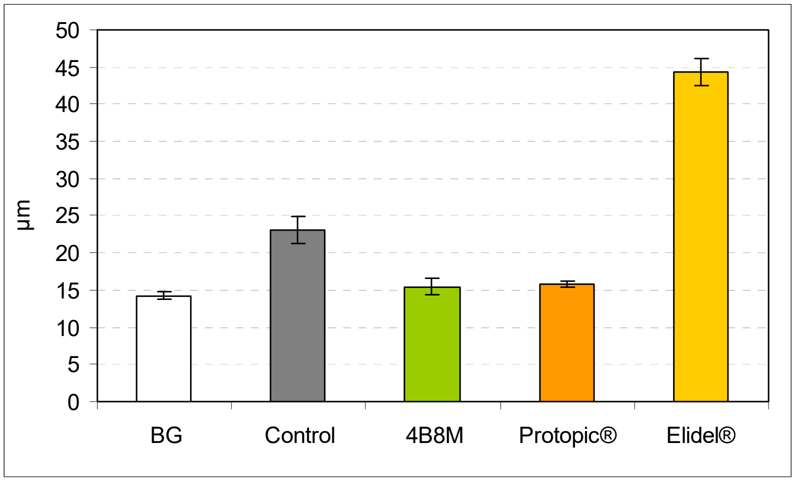

Non specific skin inflammation induced by salicylic acid – Materials and Methods

We followed a method described elsewhere [Patrick et al. 1987]. 20% salicylic acid (SA) solution in 96% ethanol was prepared and was applied for 3 days (5× per day every 30 min) on both sides of the auricles (20 µl portions). Mice from the BG group were given only 96% ethanol. After last application of SA on a given day (following 30 min of last SA dose), the auricles were smeared with the vehiculum (the BG and control groups), 0.1% 4B8M in ointment, Protopic® or Elidel®. 24h after the last SA application (day 3rd of the test) the parameters of the inflammatory reaction were determined.

<u>Determination of skin vessel permeability</u>

The test was performed according to Katayama et al, 1978 with minor modifications. Mice were given 1 mg of Evans blue in 0.2 ml of 0.9% NaCl, intravenously, on day 3 of the test. After 30 min the mice were sacrificed by cervical dislocation, the auricles cut off and immersed in 50 µl of 1 M KOH for 18h at 37 ºC. The dye was extracted from the tissue using a mixture of 450 µl of 0.2 M phosphoric acid and acetone (5:13 ratio). The samples were centrifuged at 3,000 rpm for 15 min. OD of the supernatants was measured at 550/630 nm. The amount of Evans blue (µg/ml) was determined from a standard curve. The results were presented as amount of the dye per 100 mg of wet tissue.

Therapeutic effect of 4B8M peptide on skin inflammation induced by salicylic acid

<u>Effect of the preparations on ear edema</u>

Fig. S18 shows effect of the 4B8M (0.1% in ointment), Protopic® and Elidel® on ear edema induced by the multiple application of SA. 24h after the last dose of SA the intensity of the reaction was evaluated by measurement of ear thickness and the background values were subtracted. The results indicate identical inhibitory effects of 4B8M and Protopic® on the ear edema. The effect of Elidel® was weak and statistically not significant.

Fig. S18. Effect of the preparations on ear edema induced by SA.

Statistics: Control vs 4B8M P=0.0001; Control vs Protopic® P=0.0001; Control vs Elidel® NS (P=0.2313); 4B8M vs Protopic® NS (P=0.9863); 4B8M vs Elidel® P=0.0001 (ANOVA).

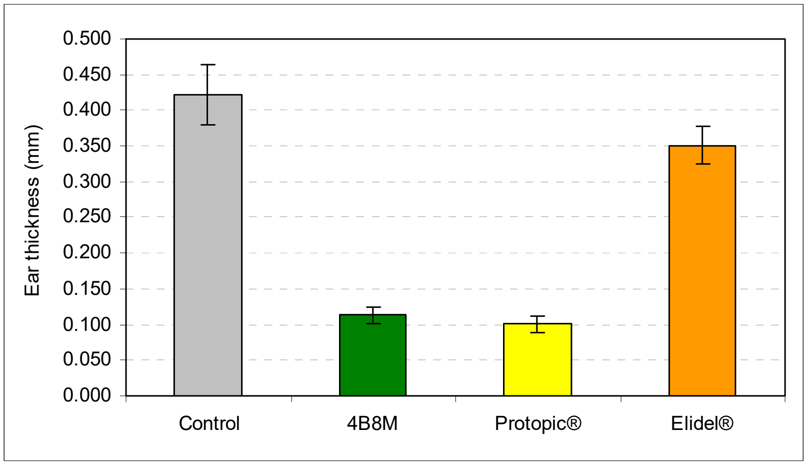

Effect of the preparations on blood leukocyte numbers

Effect of the preparations on blood leukocyte numbers was shown in **Fig. S19**. The leukocyte numbers were strongly elevated in the control group. 4B8M most effectively lowered the leukocyte number (by 57%), even below the values observed in the BG group. The reference compounds Protopic® and Elidel® were less effective (about 35% inhibition).

Fig. S19. The effect of the compounds on peripheral blood leukocyte number in mice SA-treated.

Statistics: BG vs Control P=0.0062; Control vs 4B8M P=0.0005; Control vs Protopic® P=0.0487; Control vs Elidel® P=0.0450; 4B8M vs Protopic® NS (P=0.3215); 4B8M vs Elidel® NS (P=0.3394) (ANOVA).

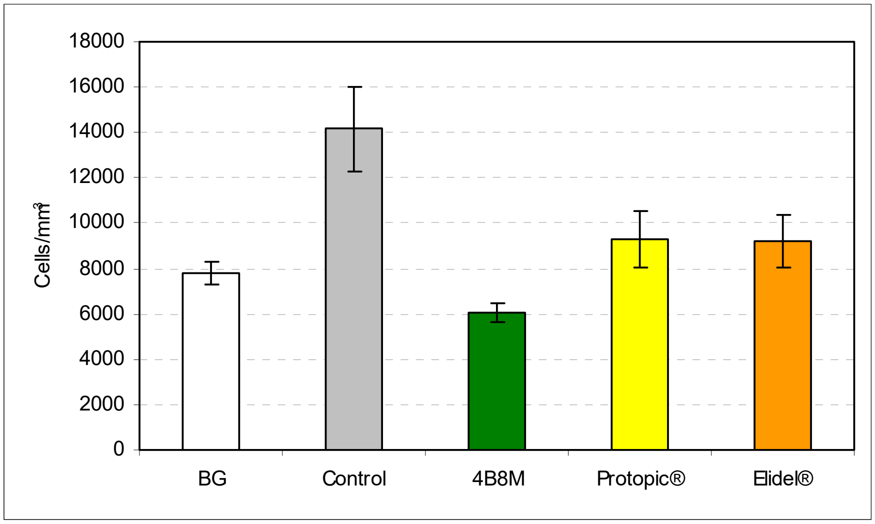

Effect of the preparations on blood cell picture

Effect of the preparations on blood picture of mice treated with SA is presented in **Fig. S20**. In control inflammed mice an increase of band forms (immature neutrophils) and eosinophils was found. 4B8M peptide almost totally reduced the percentage of band forms and eosinophils (statistical significance). Similar effects were observed in Protopic®-treated mice. Treatment of mice with Elidel® led to considerable distortion of the blood picture (an increase of bands and eosinophils).

Fig. S20. Effect of the preparations on blood cell type composition.

Statistics: bands (B): BG vs Control P=0.0001; Control vs 4B8M P=0.0001; Control vs Protopic® P=0.0001; Control vs Elidel® P=0.0427; 4B8M vs Protopic® NS (P=0.9813); 4B8M vs Elidel® P=0.0001 (ANOVA); segments (S): BG vs Control NS (P=0.9999); Control vs 4B8M NS (P=0.5196); Control vs Protopic® NS (P=0.1921); Control vs Elidel® P=0.0026; 4B8M vs Protopic® NS (P=0.9607); 4B8M vs Elidel® P=0.0001 (ANOVA); eosinophils (E): BG vs Control P=0.0001; Control vs 4B8M P=0.0001; Control vs Protopic® P=0.0001; Control vs Elidel® NS (P=0.5235); 4B8M vs Protopic® NS (P=0.9215); 4B8M vs Elidel® P=0.0001 (ANOVA); lymphocytes (L): BG vs Control P=0.0098; Control vs 4B8M NS (P=0.2504); Control vs Protopic® NS (P=0.8576); Control vs Elidel® NS (P=0.0610); 4B8M vs Protopic® NS (P=0.7982); 4B8M vs Elidel® NS (P=0.9444) (ANOVA); myeloid cells (B+S+E): BG vs Control P=0.0114; Control vs 4B8M NS (P=0.2508); Control vs Protopic® NS (P=0.8578); Control vs Elidel® NS (P=0.0612); 4B8M vs Protopic® NS (P=0.7984); 4B8M vs Elidel® NS (P=0.9445) (ANOVA).

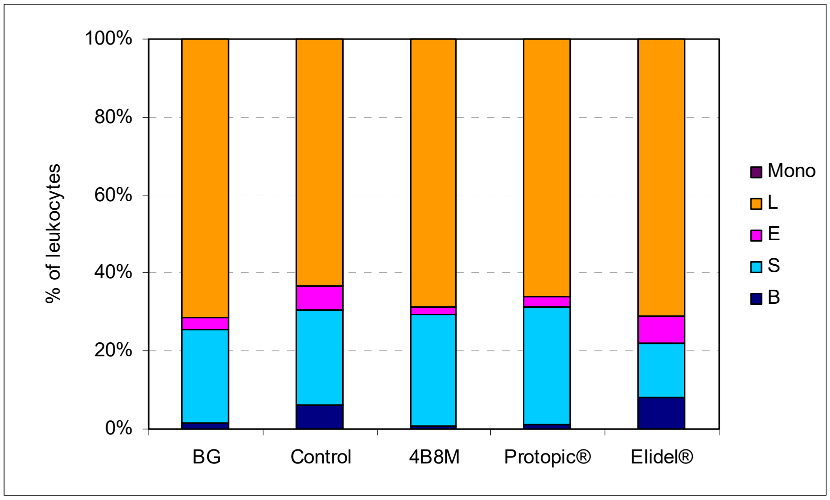

Effect of the preparations on lymph node cell numbers

Effects of the preparations of cell numbers in draining lymph nodes were presented in **Fig. S21**. In mice with induced inflammation (control group) more than 2-fold increase of the cell numbers was observed. A significant decrease of cell numbers were noted in mice treated with the 4B8M tetrapeptide and a somewhat weaker in mice treated with Protopic®, Elidel® was without effect.

Fig. S21. Effect of the preparations on lymph node cell numbers.

Statistics: BG vs Control P=0.0000; Control vs 4B8M P=0.0045; Control vs Protopic® NS (P=0.0540); Control vs Elidel® NS (P=1.0000); 4B8M vs Protopic® NS (P=1.0000); 4B8M vs Elidel® P=0.0008 (ANOVA of K-W).

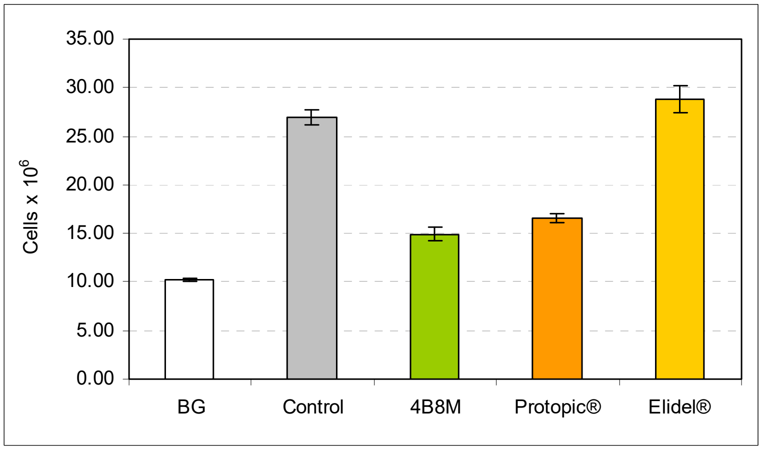

Effect of the preparations on skin blood vessel permeability

Effect of the preparations on permeability of skin vessels of the auricles was presented in **Fig. S22**. The measurement of that parameter revealed a 4-fold increase in the control group as

compared to the BG naive group. The best inhibitory effects showed 4B8M peptide and a slightly weaker Protopic®, Elidel® was less effective.

Fig. S22. Effect of the preparations on skin blood vessel permeability.

Statistics: BG vs Control P=0.0001; Control vs 4B8M P=0.0009; Control vs Protopic® P=0.0021; Control vs Elidel® NS (P=0.1529); 4B8M vs Protopic® NS (P=0.9978); 4B8M vs Elidel® NS (P=0.2906) (ANOVA).

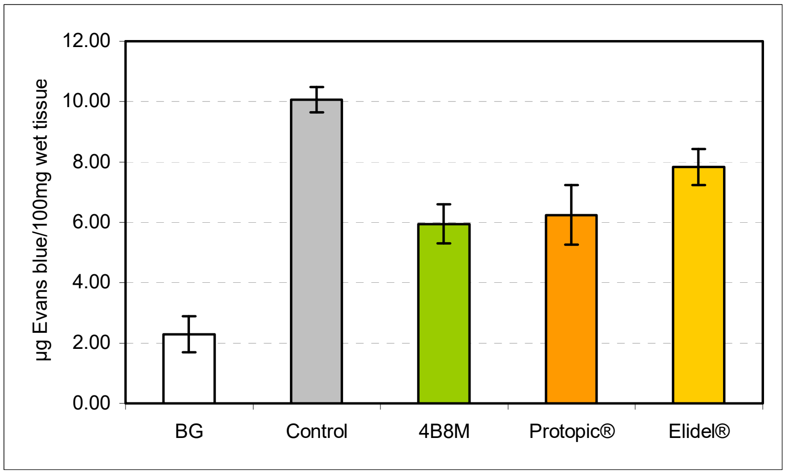

The normalizing effects of the peptide on the inflammatory skin histological changes induced by SA, are presented in **Fig. S23**.

Fig. S23AB. The histology of the auricle of BG mice. **A.** H&E staining, **B.** toluidine blue staining. In the naive mice the histological picture presented a normal tissue. Toluidine blue staining showed rare mastocytes of a low grade of granule saturation (they occurred in a condensed form).

Abbreviations: Ep – epidermis, EC – elastic cartilage, MT – muscle tissue, CT – connective tissue, mast – mastocyte, Ne – neutrophil, Mø – macrophage, L – lymphocyte, P – pustule

Fig. S23A.

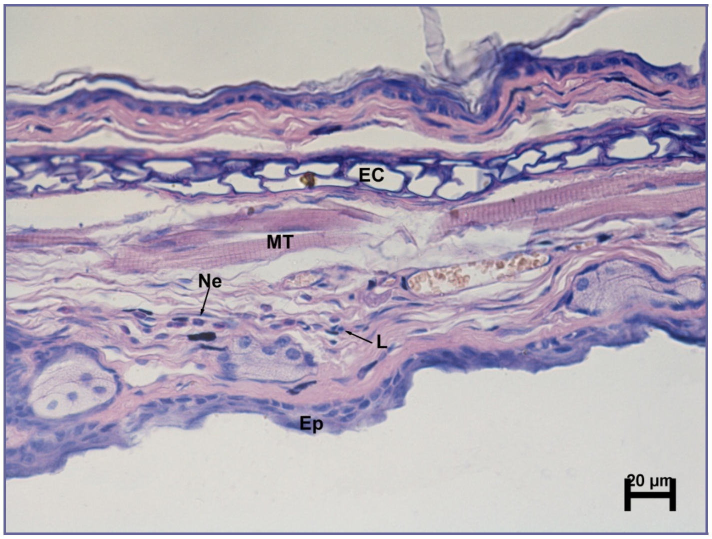

Fig. S23B.

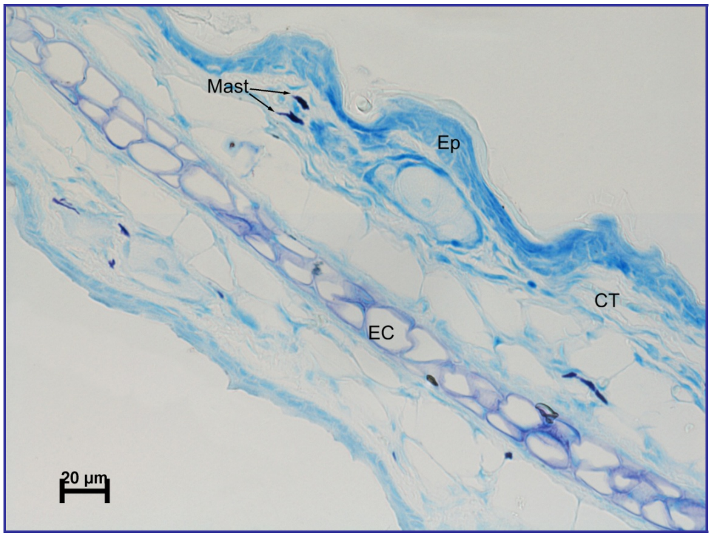

Fig. S23CD. The histological picture of auricles of control (fully developed inflammation), untreated mice. **C.** H&E staining, **D.** toluidine blue staining. The histological picture of auricles from the control group presented severe inflammation. In the dermis extensive inflammatory infiltrations mainly composed of neutrophils. The infiltrations were found under epidermis, around blood vessels, in vicinity of glands and near the elastic cartilage. In addiction the blood vessels were dilated and congested, focally extravasations were found. In the dermis areas of necrosis were also detected. The morphometric analysis of preparations stained with toluidine blue did not revealed a significant increase of mastocyte numbers. In the epidermis acanthosis, spongiotic changes and formation of neutrophilic pustules were noted.

Fig. S23C.

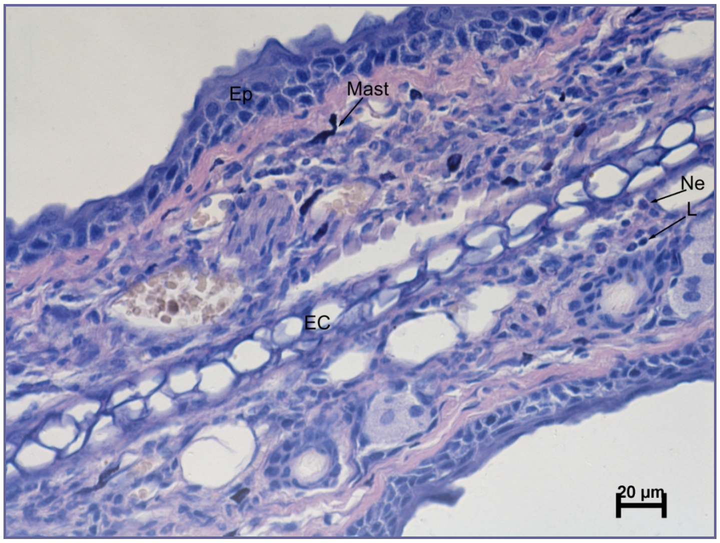

Fig. S23D.

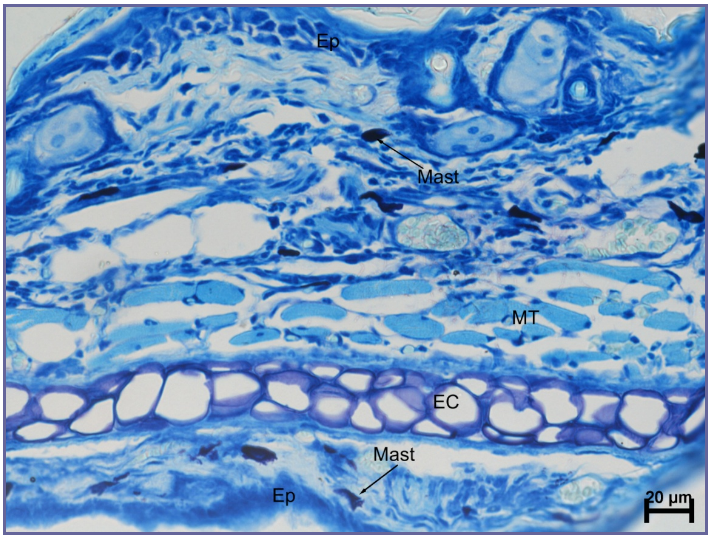

Fig. S23EF. The histological picture of auricles after 48h following elicitation of the contact sensitivity reaction in 4B8M-treated mice. **E.** H&E staining, **F.** toluidine blue staining. In mice treated with the peptide granulocytic infiltrations in the perivascular zone of dermis and in the subepithelial zone were observed, albeit of a lesser intensity as in mice treated with Elidel® and the control group. Morphometric analysis showed a decrease of neutrophil number within the infiltrations. Mastocyte numbers rose not significantly in comparison to other groups. Mast cells were in a formation state and condensation of granules. Inflammation changes (pustules) and necrosis were observed less frequently in comparison to control and Elidel® groups.

Fig. S23E.

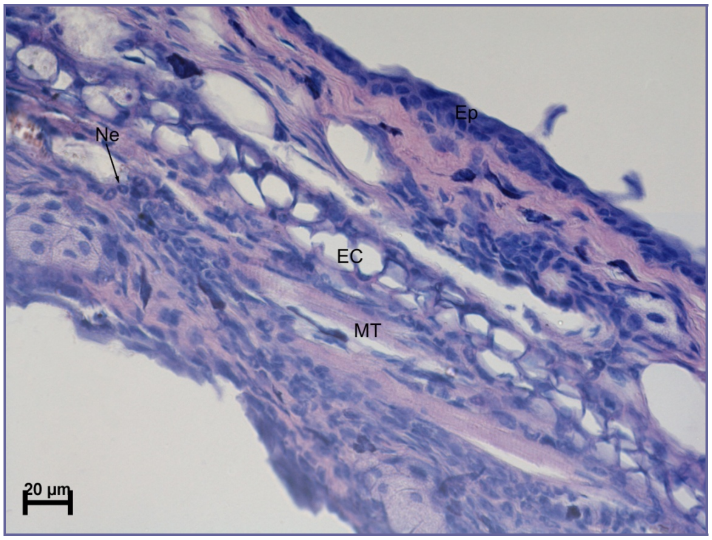

Fig. S23F.

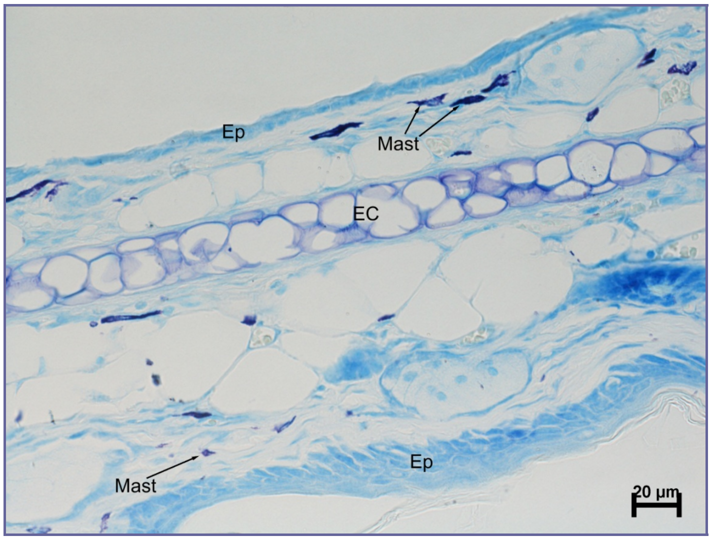

Fig. S23GH. The histological picture of auricles in Protopic®-treated mice. **G.** H&E staining, **H.** toluidine blue staining. In mice treated with Protopic® silencing of the inflammatory process, both in the dermis and epidermis, were observed. The number of neutrophils in the dermis decreased.

Fig. S23G.

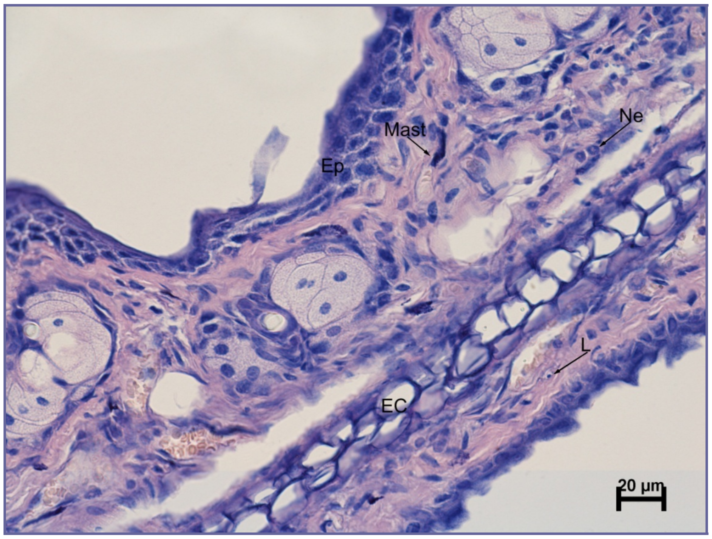

Fig. S23H.

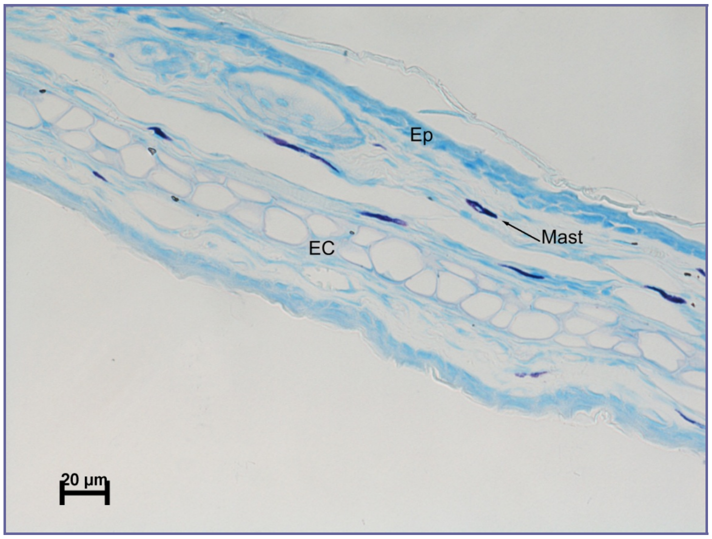

Fig. S23IJ. The histological picture of auricles in Elidel®-treated mice. **I.** H&E staining, **J.** toluidine blue staining. In mice treated with Elidel® the histological picture of auricles presents an inflammatory state in the dermis, however less expressed than in the control group because of a fewer number of neutrophils in the connective tissue of the dermis. No increase of mastocyte numbers was noted, which were in majority in a condensed state. The inflammatory process was strongly expressed in the epidermis. Exocytosis of neutrophils and lymphocytes into the epidermis was observed. A presence of neutrophilic pustules in various development stages were registered. In some areas a disruption of the epidermal integrity was found. Similarly as in the control group, the areas of epidermal necrosis occurred.

Fig. S23I.

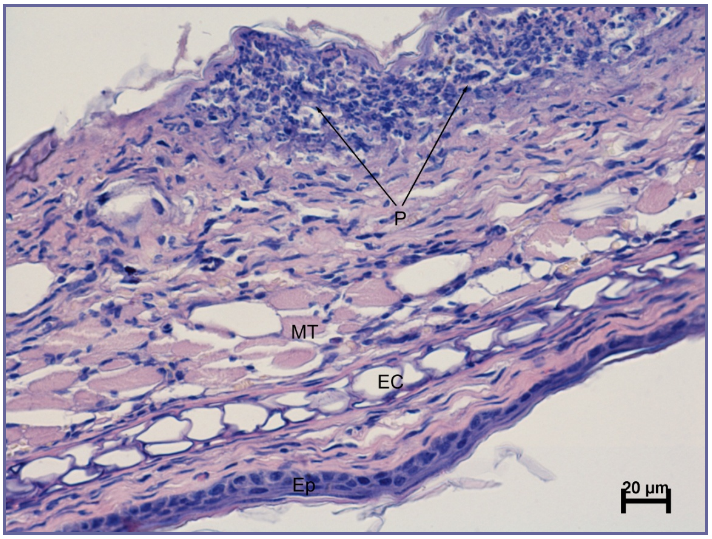

Fig. S23J.

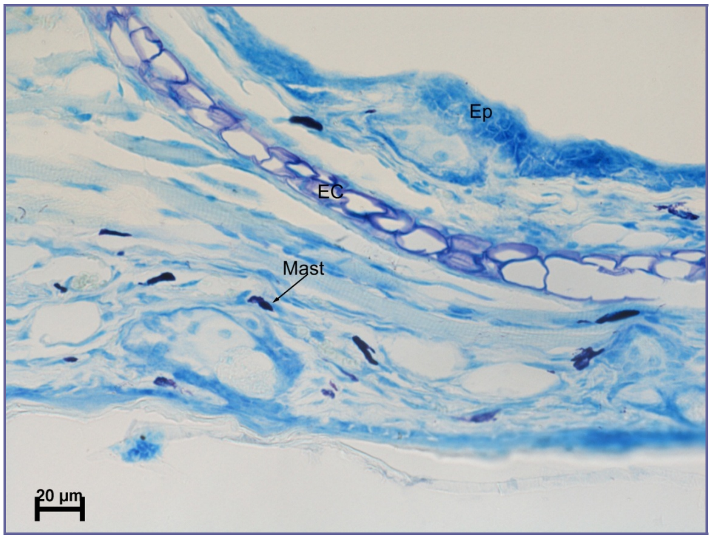

Generally, 4B8M peptide had a similar potency as Protopic® in the SA-induced inflammation model, with a more beneficial effects of Protopic® in histological analysis. However, 4B8M had a better influence on blood picture and was devoid of toxicity in contrast to Protopic® (tacrolimus). Elidel® (pimecrolimus) exhibited undesirable effects.

Carrageenan-induced inflammation in air pouch – Materials and Methods

A method described by Bottomley et al. 1988 was used. The model of carrageenan-induced inflammation in the air pouch is useful for evaluation of potential anti-inflammatory properties of compounds. An advantage of the test is a moderate inflammation process and

occurrence of granuloma. The air pouch was formed in CBA mice by a s.c. injection into the dorsal region (in a halothane anesthesia) of 5 ml of air (needle 23G×11/4.5 ml syringe). On a next day the air pouch was given an additional 1 ml of air and an inflammatory process was elicited by an injection of 1% of carrageenan in PBS. The tetrapeptide (100 µg/0.2 ml) was given i.p. into mice. Indomethacin was diluted in 0.9% NaCl and administered at a dose of 100 µg/mouse and dexamethasone was diluted in 0.9% NaCl and administered at a dose of 100 µg/mouse i.p. Aspirin was dissolved in 0.9% NaCl and given at a dose of 6 mg/mouse, per os using a stomach tube. The compounds were administered at 24h following formation of the air pouch (day 1 of the test). After 90 min carrageenan was injected into the air pouch. The mice form the BG group were given 0.9% NaCl into the air pouches. Control mice were given carrageenan into the air pouches and appropriately diluted DMSO i.p.

On a next day after carrageenan injection (2nd day of the test) mice were subjected to halothane anesthesia and blood was taken from the retroorbital plexus. From the blood smears were made, dried and stained with May-Grünwald and Giemsa reagents. The blood was diluted with Türk's solution for determination of circulating leukocytes. Exudates cells were harvested by injection of 1 ml of 0.9% NaCl into the air pouches and a subsequent aspiration by means of a syringe. In the exudates the cell numbers were counted and after exudates centrifugation the cell smears were performed form the cell pellets for cell types differentiation. The smears were dried and stained with May-Grünwald and Giemsa reagents.

Therapeutic effect of 4B8M peptide on carrageenan-induced inflammation in air pouch

Effect of the preparations on the number of cells in the air pouches

Fig. S24 presents effects of administration of the peptide and the reference drugs (aspirin, indomethacin and dexamethasone), on the number of cells in the air pouches. In the control, carrageenan-treated mice, a very significant increase of the cell number in the exudates was observed (from 0.48×10^6 cells in the BG group to 1.7×10^7 in the control). The applied preparations strongly and significantly decreased cell numbers (48BM to 4.02×10^6, dexamethasone to 2.25×10^6, indomethacin to 1.84×10^6 and aspirin to 2.23×10^6).

Fig. S24. Effect of the compounds on cell numbers in the inflamed air pouches exudates.

Statistics: BG vs Control P=0.0001; Control vs 4B8M P=0.0001; Control vs dexamethasone P=0.0002; Control vs indomethacin P=0.0001; Control vs aspirin P=0.0001; 4B8M vs dexamethasone NS (P=0.8133); 4B8M vs indomethacin NS (P=0.9727); 4B8M vs aspirin NS (P=0.9727) (ANOVA).

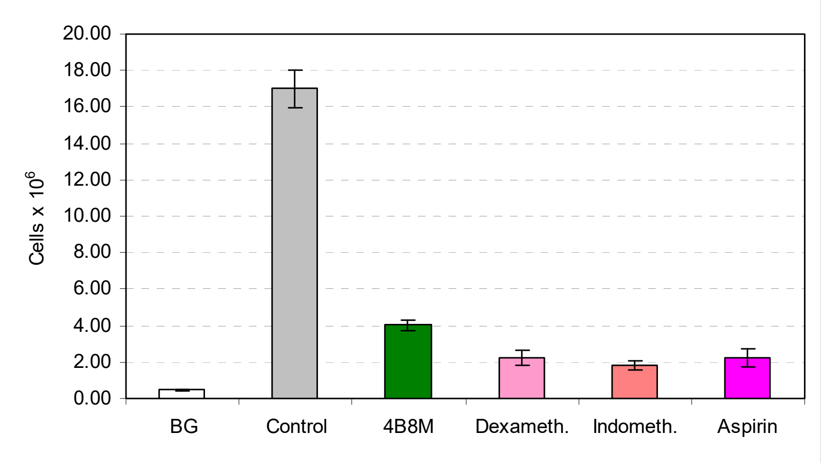

Effect of the preparations on degranulation of mastocytes in the air pouches

Carrageenan reaction is initiated by degranulation of skin mastocytes [Alves et al. 2017]. **Fig. S25** illustrates effects of the studied compounds on degranulation of mastocytes in the air pouches. In mice not treated with carrageenan (BG group) all mastocytes were intact. In turn, in the control group with non-treated inflammation, the contribution of degranulated mastocytes was 67%. Among the applied compounds only dexomethasone did not significantly affect the percentage of degranulated mastocytes. The highest increase of non-degranulated mastocytes occurred after application of the peptide (75.5%). Indomethacin and aspirin increased the content of intact mastocytes to 64.8% and 54.7%, respectively.

Fig. S25. Effect of the compounds on degranulation of mastocytes in the air pouch exudates.

Statistics: degranulated Mast: BG vs Control P=0.0001; Control vs 4B8M P=0.0001; Control vs dexamethasone NS (P=0.8008); Control vs indomethacin P=0.0001; Control vs aspirin P=0.0022; 4B8M vs dexamethasone P=0.0001; 4B8M vs indomethacin NS (P=0.2833); 4B8M vs aspirin P=0.0035 (ANOVA); non-degranulated Mast: BG vs Control P=0.0001; Control vs 4B8M P=0.0001; Control vs dexamethasone NS (P=0.8008); Control vs indomethacin P=0.0001; Control vs aspirin P=0.0022; 4B8M vs dexamethasone P=0.0001; 4B8M vs indomethacin NS (P=0.2833); 4B8M vs aspirin P=0.0035 (ANOVA).

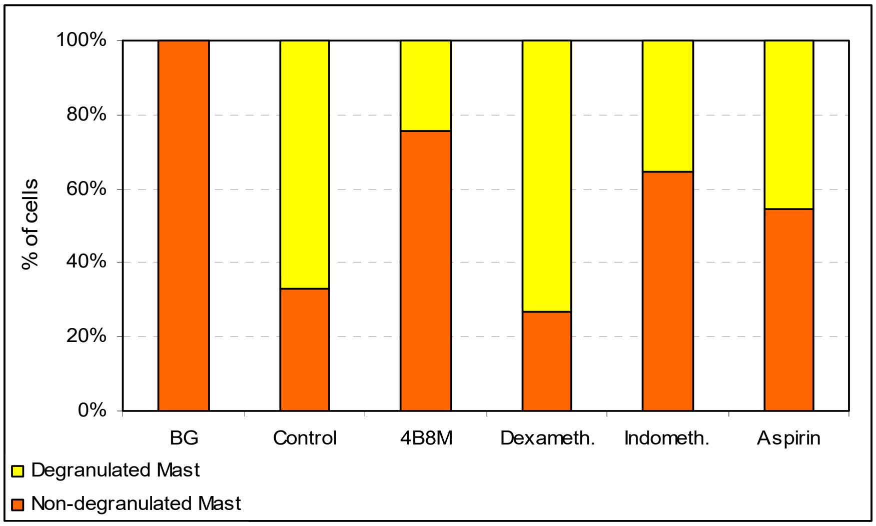

<u>Effect of the preparations on the circulating blood cell composition</u>

Fig. S26 presents the effects of the compounds administration on the circulating blood cell composition. The blood of control mice contained an unusually high percentage of immature and mature neutrophils and more eosinophils as compared to BG mice. The peptide caused a normalization of the blood picture almost to the BG one. Dexamethasone minimally changed the blood picture (some increase of neutrophils), similarly as indomethacin (increase of band forms). The down-regulatory effect of aspirin was also not significant.

Fig. S26. Effect of the compounds on the peripheral blood cell picture.

Statistics: <u>Band forms (B)</u>: BG vs Control NS (P=0.5146); Control vs 4B8M NS (P=0.0920); Control vs dexamethasone NS (P=0.9999); Control vs indomethacin P=0.0001; Control vs aspirin NS (P=0.9802); 4B8M vs dexamethasone NS (P=0.0642); 4B8M vs indomethacin P=0.0001; 4B8M vs aspirin NS (P=0.3207) (ANOVA); <u>Segments (S)</u>: BG vs Control P=0.0001; Control vs 4B8M P=0.0003; Control vs dexamethasoneNS (P=0.6533); Control vs indomethacin NS (P=0.1711); Control vs aspirin NS (P=0.4602); 4B8M vs dexamethasone P=0.0001; 4B8M vs indomethacin NS (P=0.0719); 4B8M vs aspirin P=0.0178 (ANOVA); <u>Eosinophils (E):</u> BG vs Control NS (P=1.0000); Control vs 4B8M P=0.0183; Control vs dexamethasone NS (P=0.3891); Control vs indomethacin NS (P=1.0000); Control vs aspirin NS (P=1.0000); 4B8M vs dexamethasone NS (P=1.0000); 4B8M vs indomethacin NS (P=0.7229); 4B8M vs aspirin P=0.0235 (ANOVA K-W); <u>Lymphocytes (L):</u> BG vs Control P=0.0001; Control vs 4B8M P=0.0001; Control vs dexamethasone NS (P=0.8923); Control vs indomethacin NS (P=0.8639); Control vs aspirin NS (P=0.4625); 4B8M vs dexamethasone P=0.0001; 4B8M vs indomethacin NS P=0.0001; 4B8M vs aspirin P=0.0004 (ANOVA).

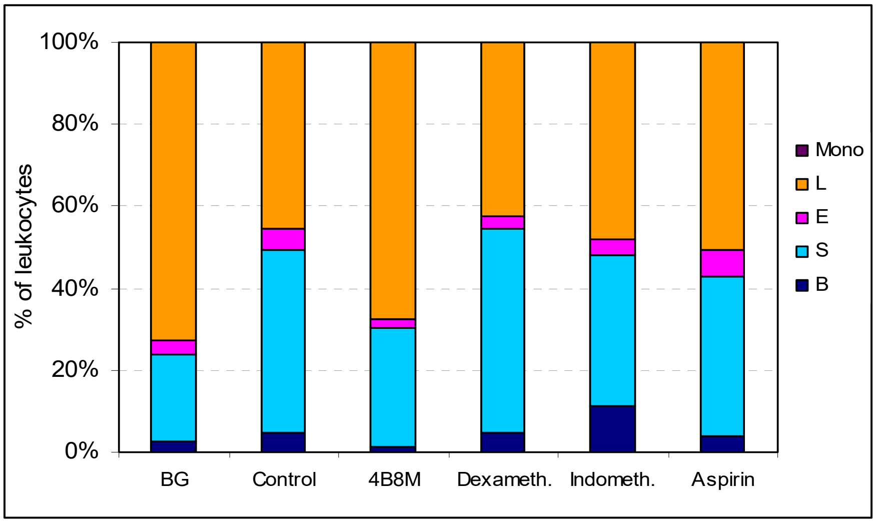

<u>Effect of the preparations on the number of circulating leukocytes</u>

The effects of the 4B8M and reference compounds on the number of circulating leukocytes is presented in **Fig. S27.** The number of leukocytes in the control group was significantly elevated. The compounds decreased the leukocyte numbers to various degrees. The weakest (not significant effect) was found in the case of aspirin. The tetrapeptide 4B8M and dexamethasone decreased the cell numbers to the values observed in the BG mice and indomethacin even to lower values.

Fig. S27. Effect of the compounds on leukocyte number in the circulating blood.

Statistics: BG vs Control P=0.0062; Control vs 4B8M P=0.0088; Control vs dexamethasone P=0.0025; Control vs indomethacin P=0.0001; Control vs aspirin NS (P=0.3960); 4B8M vs dexamethasone NS (P=0.9948); 4B8M vs indomethacin NS (P=0.3644); 4B8M vs aspirin NS (P=0.4260) (ANOVA).

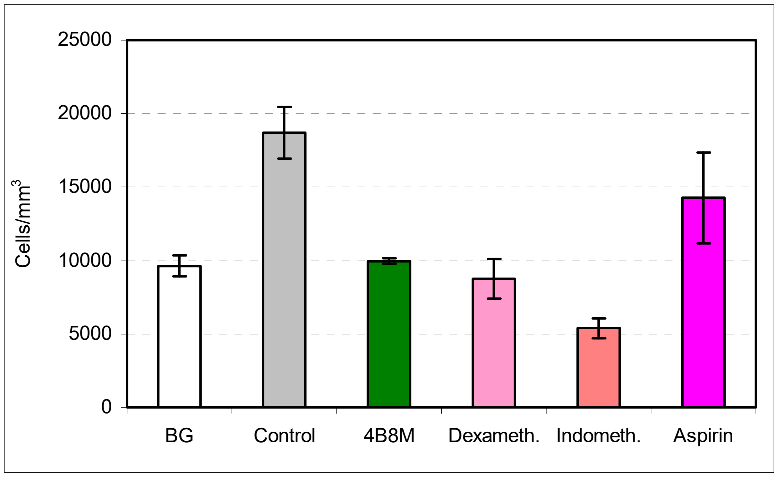

The normalizing effects of the peptide on the histological changes in air pouch induced by carrageenan, are presented in **Fig. S28**.

Fig. S28AB. The histology of the air pouch skin of BG (naive) mice. **A.** H&E staining, **B.** toluidine blue staining. The histological analysis of the air pouch skin from BG mice displayed a thin layer of the loose connective tissue, adjoining to muscles. That layer, being a result of air pouch formation, contained mainly fibrocytes and fibroblasts and single inflammatory cells (mastocytes, neutrophils and histiocytes). The histological picture of dermis is normal; 100× magnification.

Abbreviations: Ep – epidermis, D – dermis, Sc – subcutis, MT – muscle tissue, g – granuloma

Fig. S28A.

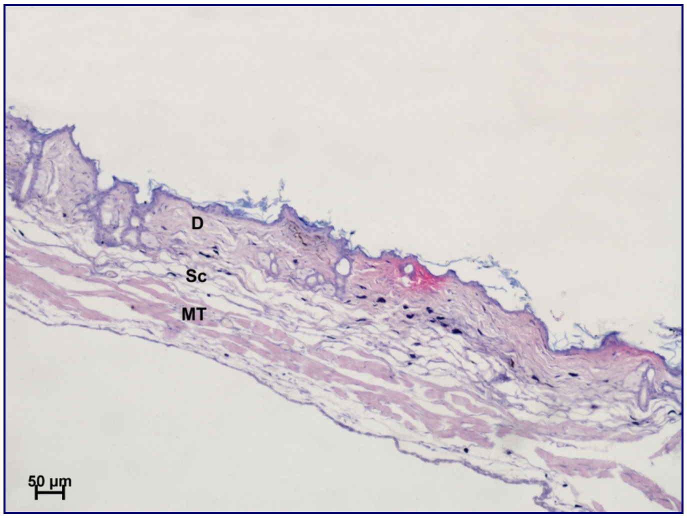

Fig. S28B.

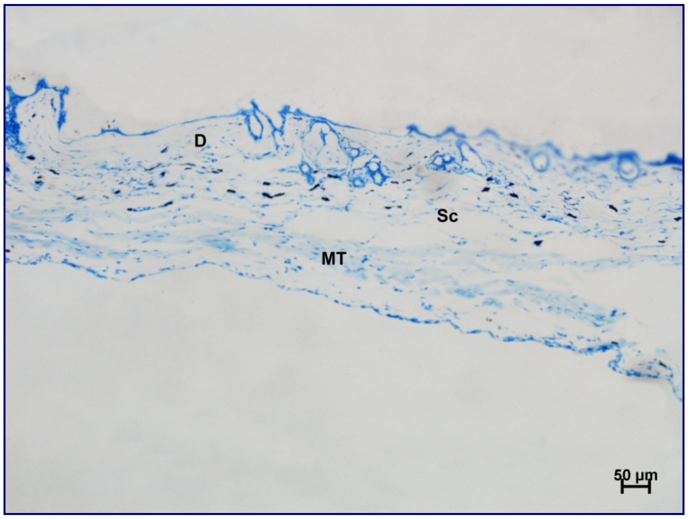

Fig. S28CD. The histology of the air pouch skin of control (inflammed) mice. **C.** H&E staining, **D.** toluidine blue staining. In the skin of mice from the control group a granuloma formation was observed, occurring in a form of thick layer adjoining superficial skin muscles. In this layer mainly: histiocytes, fibroblasts, fibrocytes and rare mastocytes, located among collagen bundles, were observed (**Fig. S28C** and **Fig. S28D**). Locally, in the granuloma, areas of collagen necrobiosis were seen, disintegration of collagen bundles were registered and changes in collagen staining (an increased basophilicity) (**Fig. S28D**). Also, the surfaces of necrobiosis were surrounded by numerous histiocytes, arranged in a form of cascades. In the interstitium of the connective tissue mucin deposits were also observed being a proof of mucoid edema. The deposits were formed due to accumulation of acid glycosaminoglycans in the connective tissue and in H&E staining they appeared as granular or strip-like material (**Fig. S28C**). Toluidine blue staining, revealing acidic glycosaminoglycans, confirmed the presence of mucins in the dermis (**Fig. S28D**). In the control group a very strong accumulation of mucins in the dermis was observed (**Fig. S28D**). The mucin deposits were seen in the dermis connective tissue (around blood vessels and skin appendages), in the connective tissue of subdermis, in the connective tissue of granuloma and in the connective, intermuscular tissue; 100× magnification.

Fig. S28C.

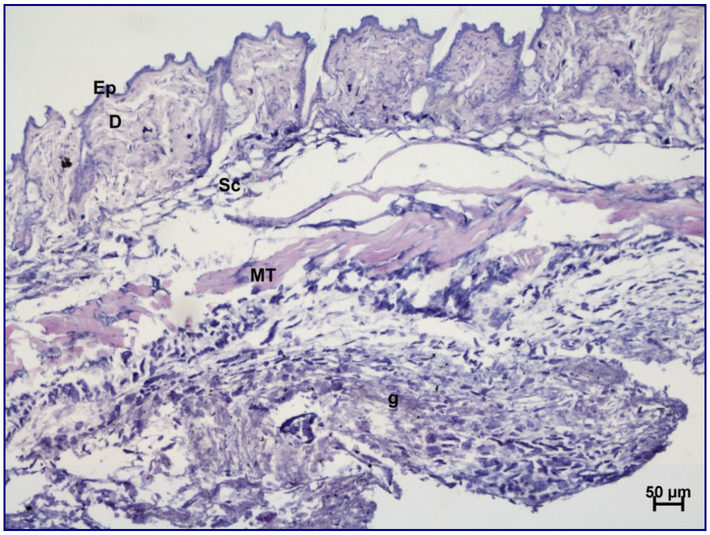

Fig. S28D.

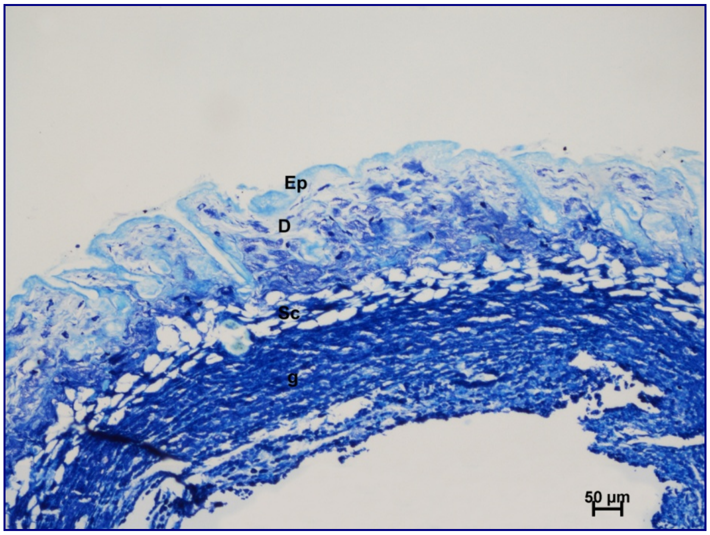

Fig. S28EF. The histology of the air pouch skin of 4B8M-treated mice. **E.** H&E staining, **F.** toluidine blue staining. In this group of mice the thickness of the granuloma was the smallest. Within the granuloma a development of a fibrous zone was observed (**Fig. S28E** and **Fig. S28F**). A diminution of mucin content in the dermis was noted, particularly around hair follicles. Toluidine blue staining revealed weaker metachromasia in this area (**Fig. S28F**). The content of mucin in the connective, intermuscular tissue and in the fatty stroma, was comparable with that in the aspirin treated-mice; 100×magnification.

Fig. S28E.

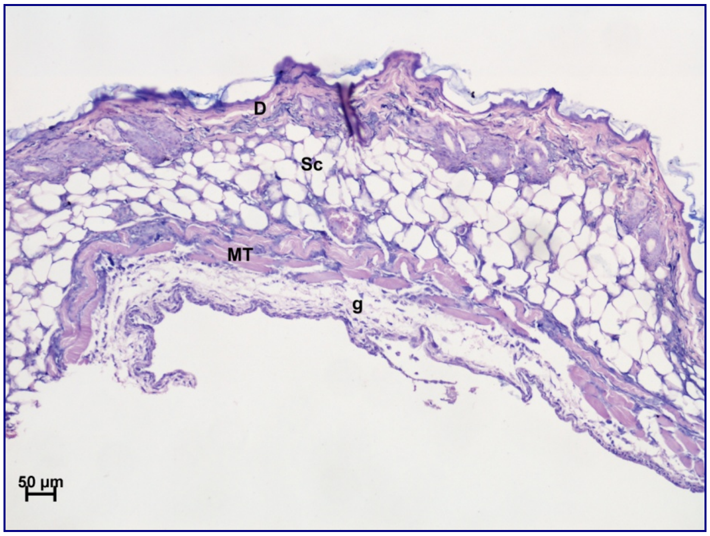

Fig. S28F.

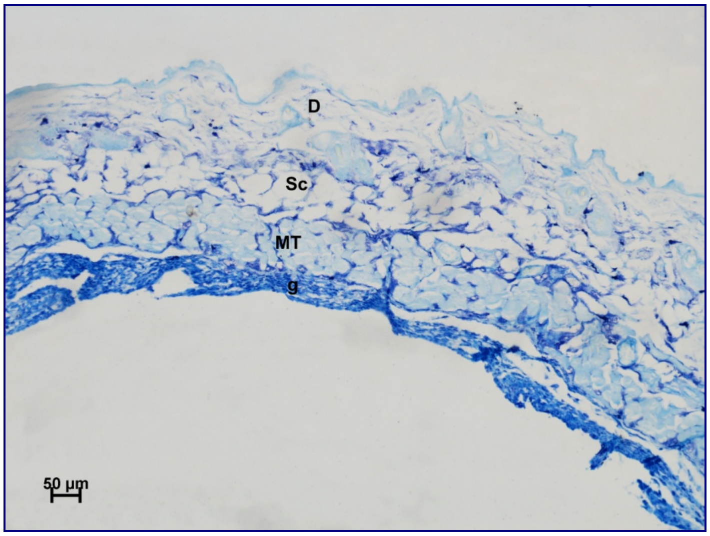

Fig. S28GH. The histology of the air pouch skin of dexamethasone-treated mice. **G.** H&E staining, 100× magnification **H.** toluidine blue staining, 400× magnification. Granuloma was also observed in the group of mice treated with dexamethasone (**Fig. S28G**). The thickness of the granuloma was not significantly smaller than that in the control group. However, no necrobiosis of collagen was observed in this group. Within the granuloma inflammatory and fibrogenic zones were found, indicating initiation of fibrogenesis. The inflamamtory cells in the granuloma were not so frequent as in the control group. In the dermis and subdermis fewer mucin deposits were found in comparison with the control group. Staining with toluidine blue revealed a weaker metachromasia of the mucin substance (**Fig. S28H**).

Fig. S28G.

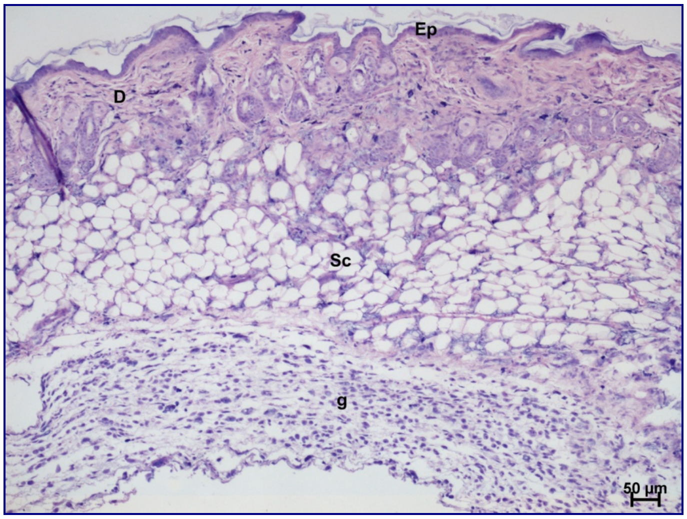

Fig. S28H.

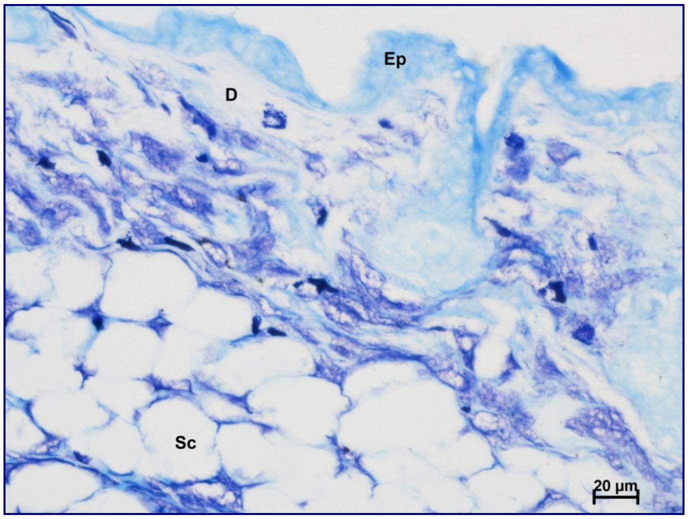

Fig. S28IJ. The histology of the air pouch skin of aspirin-treated mice. **I.** H&E staining, 100×magnification **J.** toluidine blue staining, 400× magnification. In mice treated with aspirin the thickness of granuloma was smaller in comparison with control and dexamethasone-treated groups. The occurrence of mucins was moderate in this group. Deposits of strip-like material and granular mucin were observed in the dermis, in the collagen tissue of subdermis and in the granuloma.

Fig. S28I.

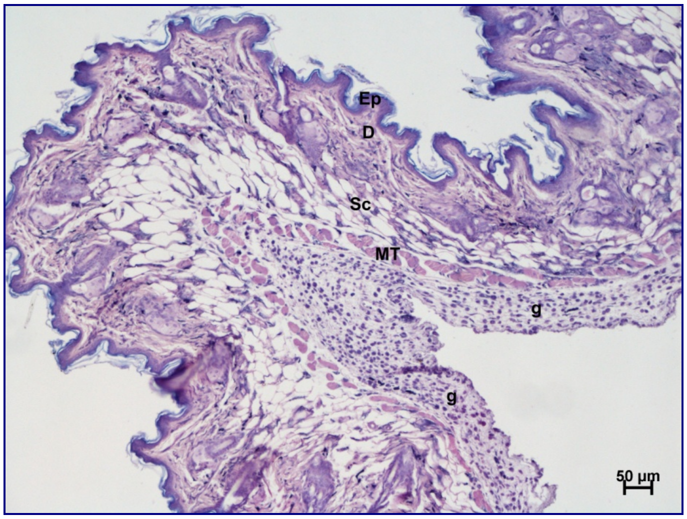

Fig. S28J.

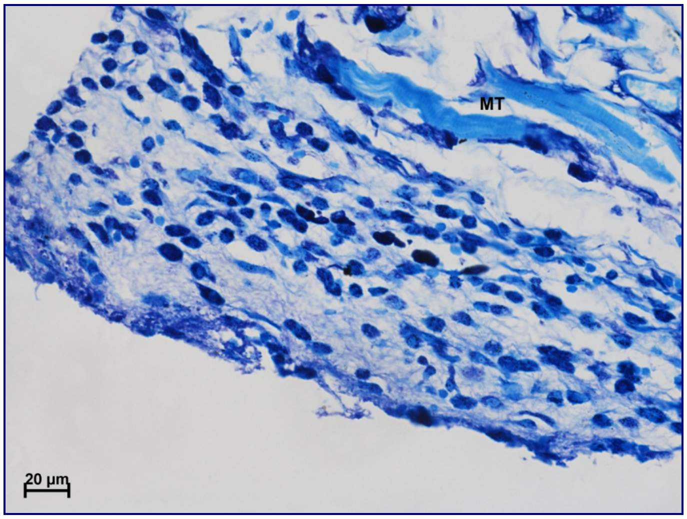

Thickness of the granuloma – a comparative study

Beside of the histological analysis of the skin also histometric measurements of the granuloma thickness were performed (**Fig. S29**). The granuloma thickness was best reduced in the group of mice treated with the peptide. Aspirin treatment was least effective and dexomethasone did not affect the thickness of the granuloma tissue.

Fig. S29. Thickness of the granuloma tissue.

Statistics: BG vs Control P=0.0000; Control vs 4B8M P=0.0000; Control vs dexamethasone NS (P=1.0000); Control vs aspirin P=0.0122; 4B8M vs dexamethasone P=0.0000; 4B8M vs aspirin NS (P=0.4151) (ANOVA).

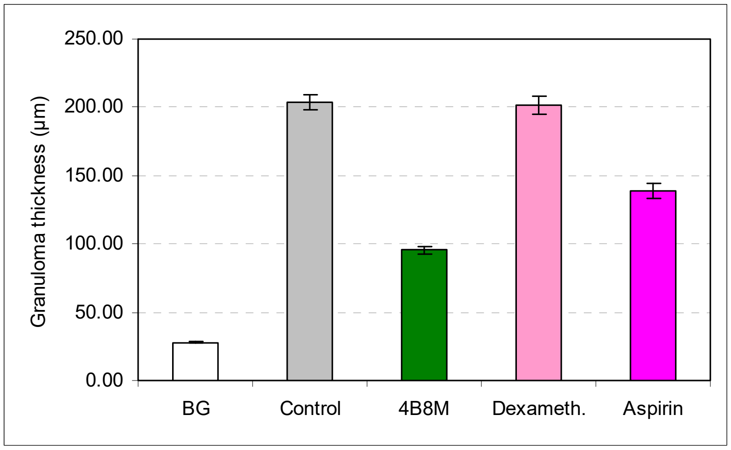

The results from the air pouch model in mice with carrageenan-induced inflammation showed that the tetrapeptide was more efficient, as compared to the reference drugs (aspirin and dexomethasone), in diminishing symptoms of inflammation. The tetrapeptide normalized more effectively cell compositions in the air pouch exudates and circulating blood. On the other hand, it was less effective in comparison with the reference drugs in lowering numbers

of exudates cells and circulating leukocytes. In the histological picture the tetrapeptide exhibited a strong anti-inflammatory and repair actions, comparable with that of aspirin. In addition, the tetrapeptide displayed the strongest action with regard to diminution of the granuloma thickness, as compared with the reference compounds.

Non-specific colon inflammation induced by dextran sulfate – Materials and Methods

The test was performed according to Melgar et al. 2005, with some modifications. C57Bl/6 mice were given 4% solution of dextran sulphate in tap drinking water acidified to pH 3.5 (with hydrochloric acid to limit growth of *Pseudomonas* species), for 6 days. Then, the mice were given only the acidified water. The tetrapeptide 4B8M was administered to mice intragastrically, by means of a stomach tube, at a dose of 250 µg in 0.2 ml, daily on days 1-5. Pentasa® (5-ASA) was given in the same way at a dose of 1.5 mg (0.15 ml). The control group received appropriately diluted DMSO solution (as in 4B8M preparation). On day 12 of the experiment the mice were subjected to halothane anesthesia and bled via retroorbital plexus, followed by cervical dislocation. The thymuses and colon were isolated.

Determination of the level of IL-1β in the colonic tissue

Colon was placed in disposable Petri dishes. Approximately 0.1 g of colonic mucosa was added to 1 ml of sterile, cold PBS with 10% FCS and protease inhibitor (cOmplete Protease inhibitor Coctail, Roche Diagnostics, Poland) and the mixture was pulverised homogeneously by passing through a nylon mesh. The samples were centrifuged and the supernatants analyzed by ELISA kit (eBioscience) for IL-1β concentration.

Therapeutic effect of 4B8M peptide on non-specific colon inflammation induced by dextran sulfate

The colonic tissue homogenate level of IL-1β was significantly lower in control group (**Fig. S30**). 4B8M peptide, and to a lesser degree 5-ASA, reversed that fall in the cytokine level.

Fig. S30. Effect of the compounds on IL-1β content in the colon.

Statistics: BG vs Control NS (P=0.2141); Control vs 4B8M P=0.0310; Control vs 5-ASA NS (P=0.6545); 4B8M vs 5-ASA NS (P=1.0000) (ANOVA K-W).

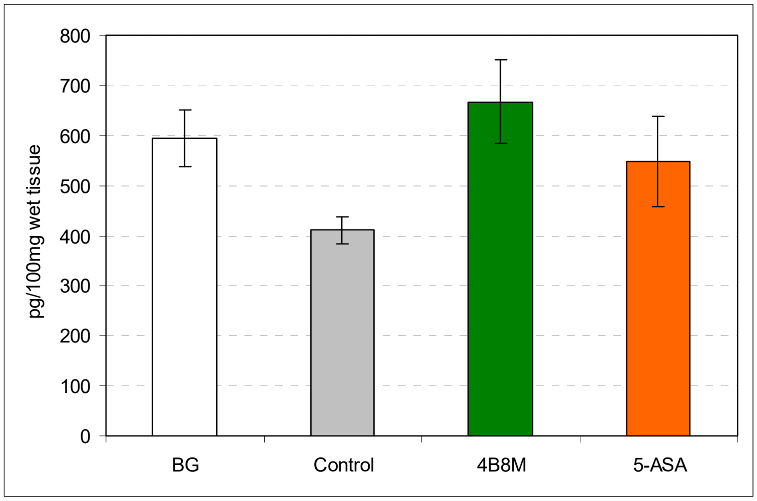

The protective effects of the peptide on the inflammatory histological changes in inflammed colon are presented in **Fig. S31**.

Fig. S31A. Colon of the mouse from BG group; H&E staining. The histological view of colon did not show histopathological lesions. A normal architecture of colon layers such as mucosa, submucosa and muscularis externa was observed. The epithelium contained many goblet cells. The surface of mucosa was flat. The crypt architecture was normal, they filled the whole width of the mucosa. In the mucosa fibroblasts, collagen fibres and single neutrophils, lymphocytes and eosinophils were observed. Small, single lymphatic nodule in the mucosa were also observed. The submucosa contained connective tissue with no signs of inflammation or fibrosis. Beneath the submucosa a normal muscularis externa was visible, consisting of 2 layers of smooth muscle cells; 200× magnification.

Abbreviations: M – mucosa, MM – muscularis mucosae, SM – submucosa, ME – muscularis externa

Fig. S31A.

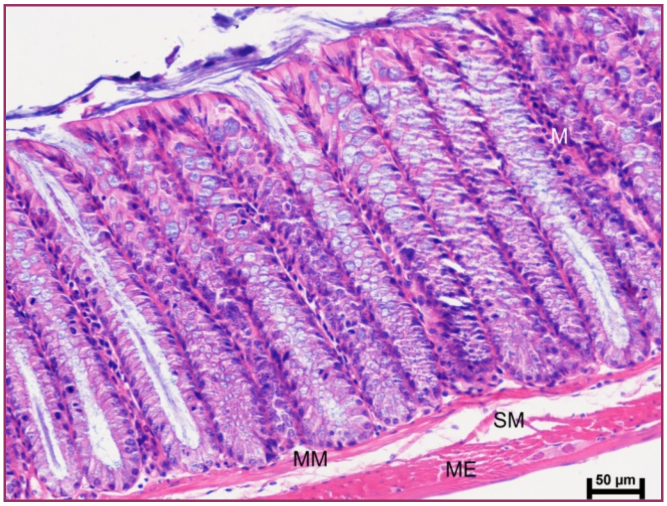

Fig. S31BC. Inflammed colon of the control mouse treated with dextran sulphate; H&E staining. In the colon wall inflammatory infiltrates were observed, consisting of lymphocytes, plasma cells, macrophages and less numerous neutrophils and eosinophils. The inflammatory infiltrates occurred mainly in the proper lamina of the mucosa. Inflammatory cells were noted both between the intestinal crypts and at the base of the crypts near the muscularis mucosae. Locally, the inflammatory infiltration disrupted the architecture of the intestinal crypts in the mucosa. Single crypt branching, crypt shortening and disappearance of the intestinal crypts were observed. In most of the analyzed images, mucus production by goblet cells (mucinogenesis) was preserved. The inflammatory process extended to the submucosa. Inflammatory cells have even been recorded in the muscularis externa. The submucosa was thickened in relation to the BG group. Besides inflammatory cells, additional bundles of collagen fibers were observed in the submucosa, which may indicate a beginning of the fibrosis process; 200× magnification (B)/100× magnification (C).

Fig. S31B.

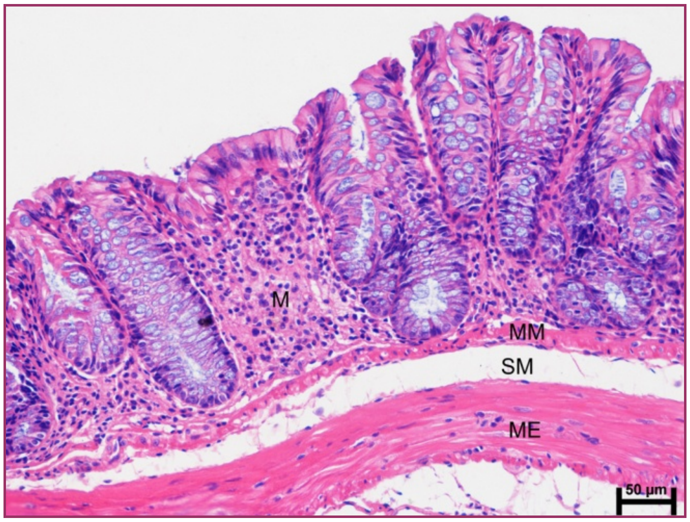

Fig. S31C.

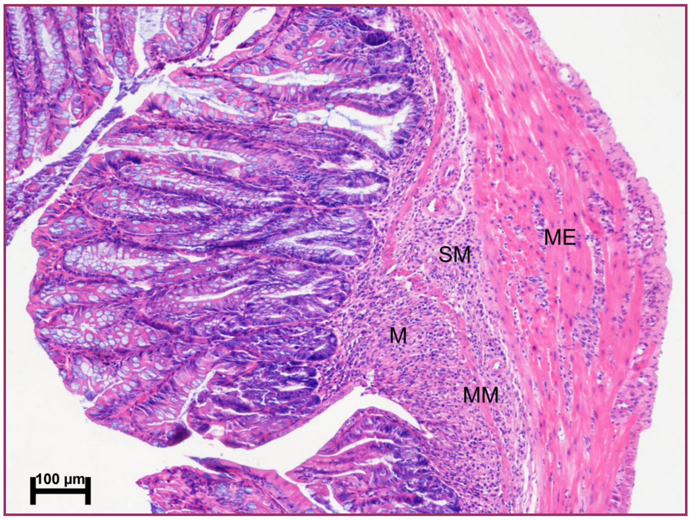

Fig. S31D. Inflammed colon of the mouse treated with 5-ASA; H&E staining. In animals treated with 5-ASA, the histological examination revealed focal inflammation of the colon mucosa. Within the lamina propria of the mucosa, inflammatory infiltrates containing lymphocytes, plasma cells, macrophages with a slight admixture of granulocytes were observed. Near the infiltrations, the intestinal crypts contained fewer mucus cells. Crypt disappearance due to inflammation was occasionally observed. The inflammatory infiltrates in the submucosa were rare and weaker as compared to the control group with full blown inflammation; 200× magnification.

Fig. S31D.

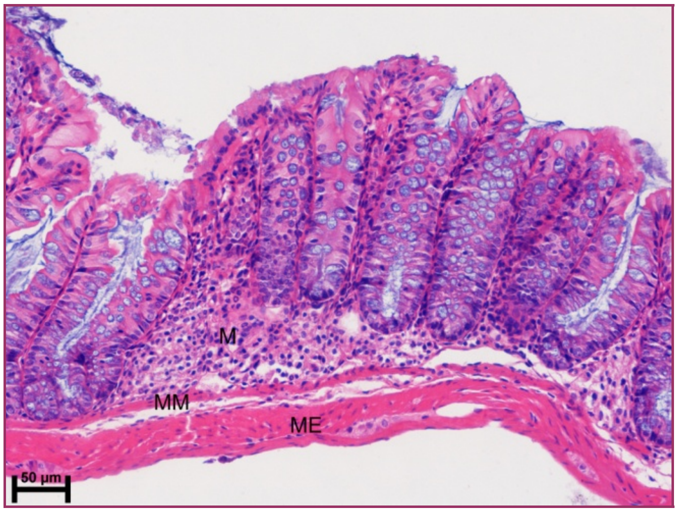

Fig. S31E. Inflammed colon of the mouse treated with 4B8M peptide; H&E staining. In the group of animals treated with 4B8M, the inflammation in the mucosa was reduced. This is evidenced by the reduced number of inflammatory cells in the lamina propria of the mucosa. Inflammatory infiltrates, consisting mainly of lymphocytes and a few neutrophils were present locally. Shortening of the intestinal crypts was occasionally observed. Submucosal inflammation was not reported; 200× magnification.

Fig. S31E.

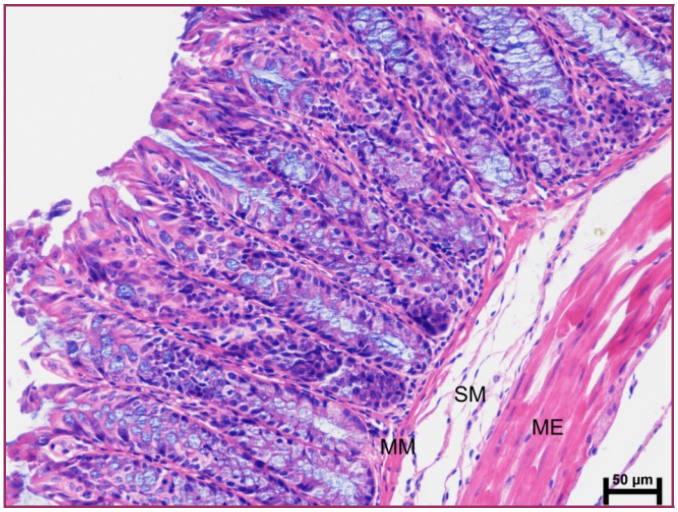

The results indicate efficacious and comparable with 5-ASA (Pentasa®) protective effects of 4B8M on inflammation parameters and histological picture of the colon. The peptide demonstrated some advantage over 5-ASA with regard to integrity of the colon tissue. The experiments also revealed a strong protecting effect of the solvent – DMSO. The majority of

the studied parameters in control mice (except cytokine levels) were altered and one mice in the control group died (11 day of the experiment).

Allergic pleurisy induced by ovalbumin – Materials and Methods

The previously described method was used (Sampaio et al. 2000). The mice were sensitized i.p. with 50 µg of ovalbumin (OVA) in Maalox (aluminii hydroxydum 3.5 g, magnesi hydroxydum 4.0 g in 100 ml; Rhone-Poulenc Rorer) as adjuvant. After 14 days the mice were injected with 12.5 µg OVA in 50 µl of 0.9% NaCl into pleural cavity by means of a syringe with 2 mm needle length. Mice from BG group were given only second (eliciting) dose of OVA. 2h before administration of the eliciting dose of antigen the mice were given *per os* (by gastic gavage) 250 µg of 4B8M in olive oil. The reference compounds – dexamethasone (125 µg/mouse) and indometacin (250 µg/mouse) were also given per os. On the next day mice were subjected to halothane anesthesia and bled via retroorbital plexus, followed by cervical dislocation. The draining lymph nodes and exudates from inflammed pleural cavity were also isolated.

Therapeutic effect of 4B8M peptide on OVA-induced allergic pleurisy

Effect of the preparations on the number of circulating leukocytes

An increase of the number of circulating leukocytes in the group of BG mice and a further increase in the control group (2× increase as compared with naive mice) was indicated (**Fig. S32**). The administration of 4B8M lowered the leukocyte number to a level registered in naive mice and to the level of BG mice by dexamethasone. Indometacin drastically lowered the leukocyte number (below the values noted in naive mice).

Fig. S32. Effect of the preparations on circulating leukocyte number.

Statistics: Naive vs BG NS (P=0.6684); BG vs Control P=0.0037; BG vs 4B8M NS (P=0.8992); BG vs Dexameth. NS (P=0.9994); BG vs Indomet. P=0.0048; Control vs 4B8M P=0.0002; Control vs Dexameth. P=0.0094; Control vs Indomet. P=0.0001; 4B8M vs Dexameth. NS (P=0.7372); 4B8M vs Indomet. NS (P=0.0731) (ANOVA).

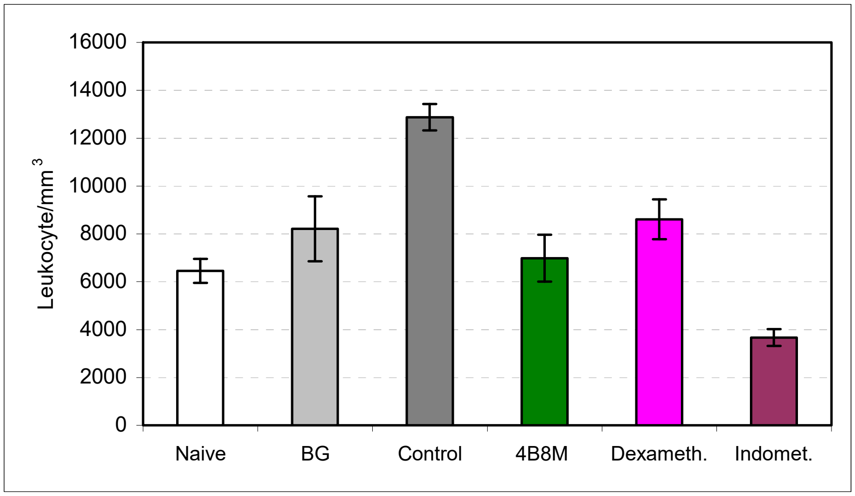

Effect of the preparations on degranulation of mastocytes in the pleural exudates

In the BG group the percentage of non-degranulated mastocytes in pleural exudates was high (55.2% of all isolated cells) and low in the control (inflammed) group (20.6%) (**Fig. S33**). The investigated compounds in a various degree increased the content of non-degranulated mastocytes: 4B8M to 70.3%, dexamethasone to 31.7% and indometacin to 63.2%.

Fig. S33. Intact (non-degranulated) and degranulated mastocytes in the pleural exudates.

Statistics: Mast intact: Naive vs BG P=0.0001; BG vs Control P=0.0001; BG vs 4B8M NS (P=0.1226); BG vs Dexameth. P=0.0033; BG vs Indomet. NS (P=0.7376); Control vs 4B8M P=0.0001; Control vs Dexameth. NS (P=0.4132); Control vs Indomet. P=0.0001; 4B8M vs Dexameth. P=0.0001; 4B8M vs Indomet. NS (P=0.8234) (ANOVA); Mast degranulated: Naive vs BG P=0.0001; BG vs Control P=0.0001; BG vs 4B8M NS (P=0.1226); BG vs Dexameth. P=0.0033; BG vs Indomet. NS (P=0.7376); Control vs 4B8M P=0.0001; Control vs Dexameth. NS (P=0.4132); Control vs Indomet. P=0.0001; 4B8M vs Dexameth. P=0.0001; 4B8M vs Indomet. NS (P=0.8234) (ANOVA).

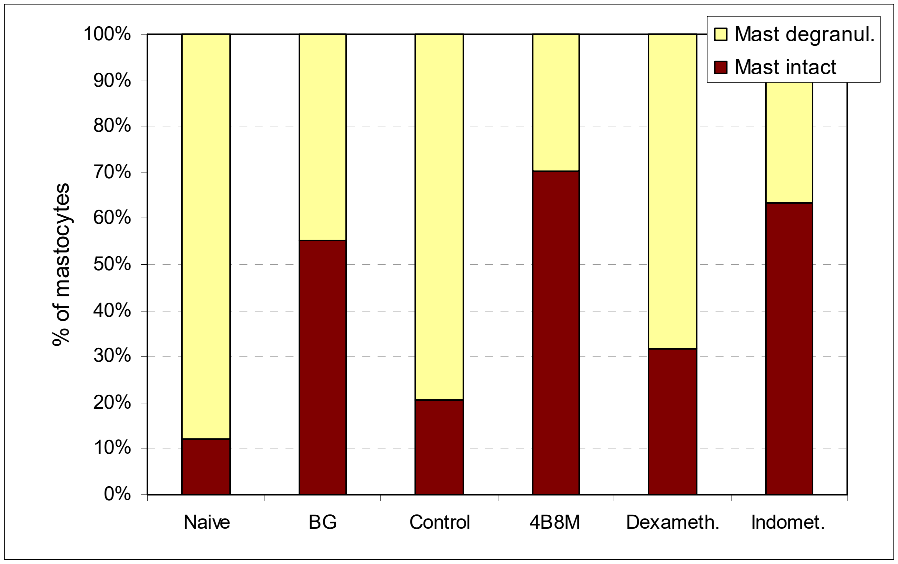

Effect of the preparations on the number of cells in draining lymph nodes

The cell number in draining lymph nodes (**Fig. S34**) was significantly elevated in inflammed control mice (1.4×10^7 vs 5.5×10^6 in naive mice and 8.8×10^6 in the BG group). The applied compounds evenly lowered the cell numbers below BG levels and near the naive mice values.

Fig. S34. Cell numbers in the draining lymph nodes.

Statistics: Naive vs BG P=0.0001; BG vs Control P=0.0001; BG vs 4B8M P=0.0001; BG vs Dexameth. P=0.0151; BG vs Indomet. P=0.0002; Control vs 4B8M P=0.0001; Control vs Dexameth. P=0.0001; Control vs Indomet. P=0.0001; 4B8M vs Dexameth. NS (P=0.6493); 4B8M vs Indomet. NS (P=0.9999) (ANOVA).

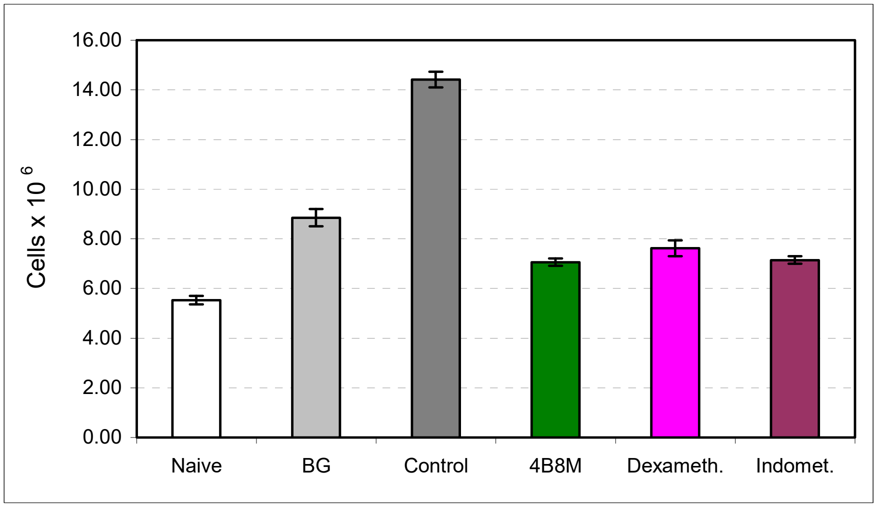

The protective effects of the 4B8M peptide on the histological changes in inflammed lungs from mice with pleurisy are presented in **Fig. S35**.

Fig. S35AB. Lungs from naive (BG) mice group; H&E staining. In naive mice no changes in the interstitium were observed. Likewise, no abnormalities were found within the bronchial and alveolar tree. In lung cross sections pleura was seen, composed of a single cell layer (mesothelium) and, situated below a very thin connective tissue layer; magnification 100× (A) and 400× (B).

Abbreviations: P – pleura, B – bronchus, A – pulmonary alveolus, Ar – artery

Fig. S35A.

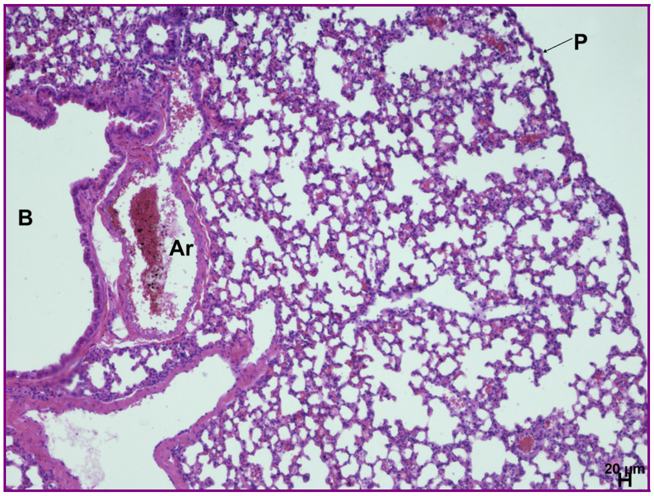

Fig. S35B.

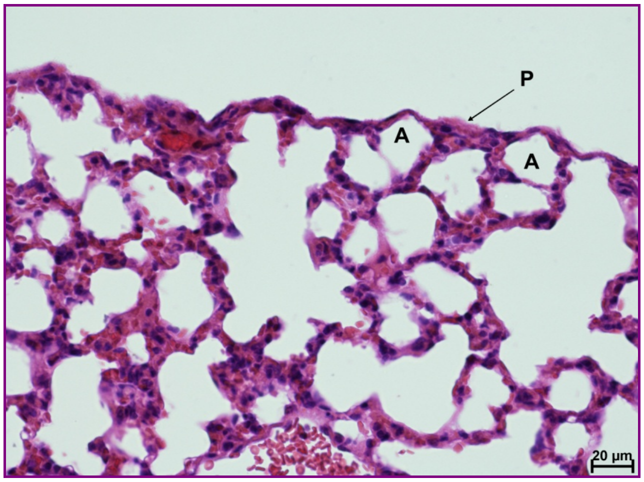

Fig. S35CD. Lungs from inflammed control mice group; H&E staining. In the lungs of animals from control group severe pulmonary edema was observed, as indicated by the presence of eosinophilic, edematous fluid in the alveolar septa and inside the alveoli. The dominant histological finding was interstitial pulmonary edema. The alveolar septa were strongly thickened by the edematous fluid accumulating within them, and the pulmonary capillaries were dilated. In addition, there was also perivascular edema. Alveolar pulmonary edema has been reported mainly in the subpleural zone and near large pulmonary vessels. Within the edematous lesions, numerous siderophages (hemosiderin-laden macrophages) and haemosiderin deposits were observed, both in the alveolar septa and inside the alveoli; hemosyderophages indicated by arrows; magnification 100× (B) and 400× (C).

Fig. S35C.

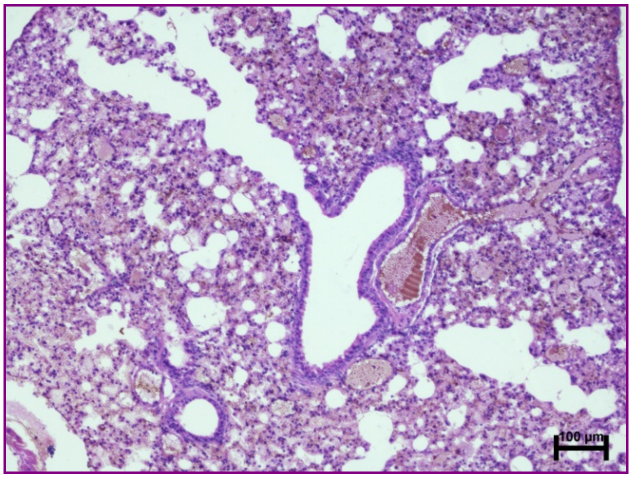

Fig. S35D.

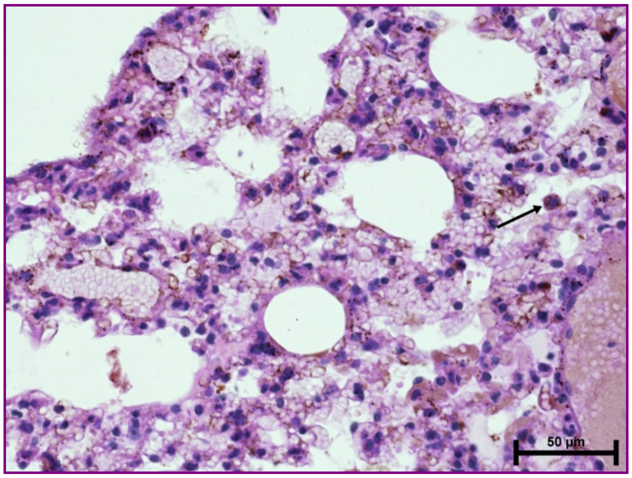

Fig. S35EF. Lungs from inflammed control mice treated with dexamethasone; H&E staining. In the lungs of dexamethasone-treated animals, the edematous lesions were weaker as compared to the control group with full blown inflammation. Foci of weak interstitial edema (**Fig. S35E**) and moderate, alveolar edema in the subpleural area (**Fig. S35F**) were observed. In addition, there were areas of lung congestion with local haemorrhages into the interstitium and alveoli (**Fig. S35E**). Numerous siderophages and haemosiderin deposits were observed in the edematous lesions as well as in the area of hyperemia and haemorrhages; hemosyderophages indicated by arrows; magnification 100× (C) and 400× (D).

Fig. S35E.

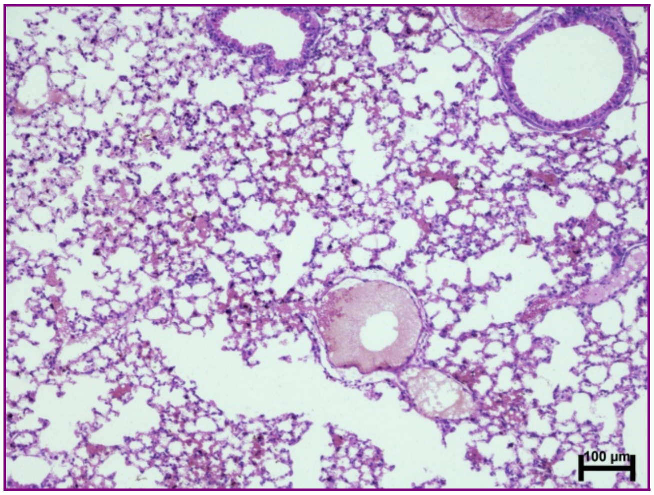

Fig. S35F.

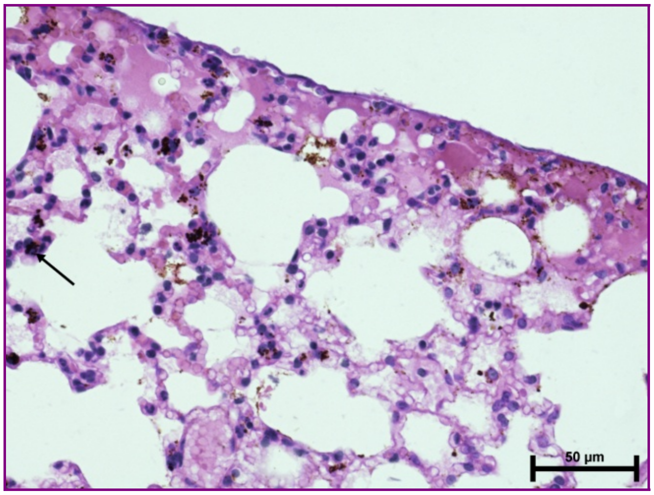

Fig. S35GH. Lungs from inflammed control mice treated with indometacin; H&E staining. In the group of animals treated with indometacin, no edematous lesions were found. Deposits of hemosiderin in the alveoli and siderophages were rarely observed. The histological pictures were closest to the normal tissue; hemosyderophages indicated by arrows; magnification 100× (E) and 400× (F).

Fig. S35G.

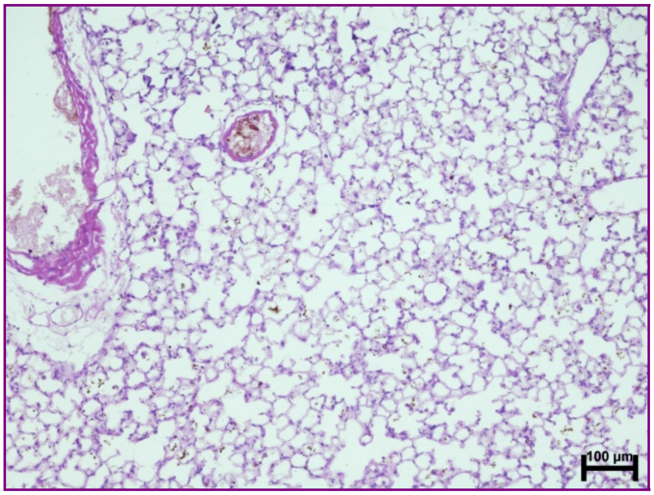

Fig. S35H.

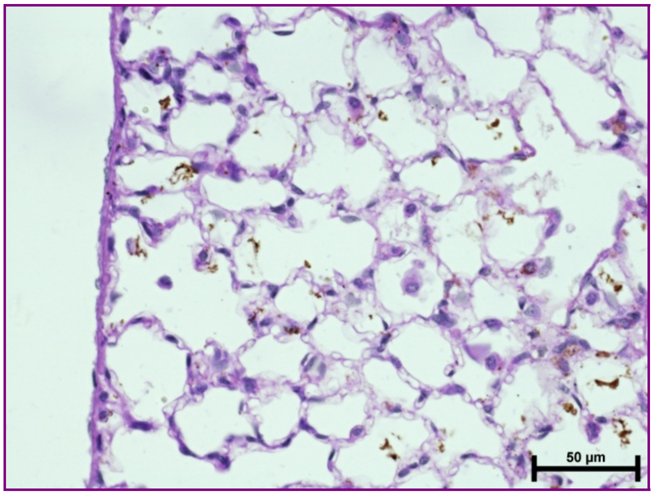

Fig. S35IJ. Lungs from inflammed control mice treated with 4B8M peptide; H&E staining. In the lungs of mice treated with 4B8M, moderate interstitial edema and mild alveolar edema were observed. These lesions were located around large pulmonary vessels and near the pleura. In the alveoli and alveolar septa, mainly hemosiderin deposits were observed, while there were fewer siderophages as compared to the control group; hemosyderophages indicated by arrows; magnification 100× (G) and 400× (H).

Fig. S35I.

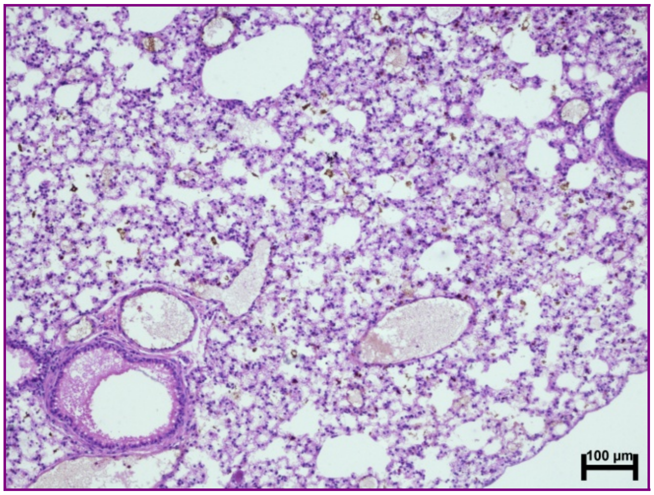

Fig. S35J.

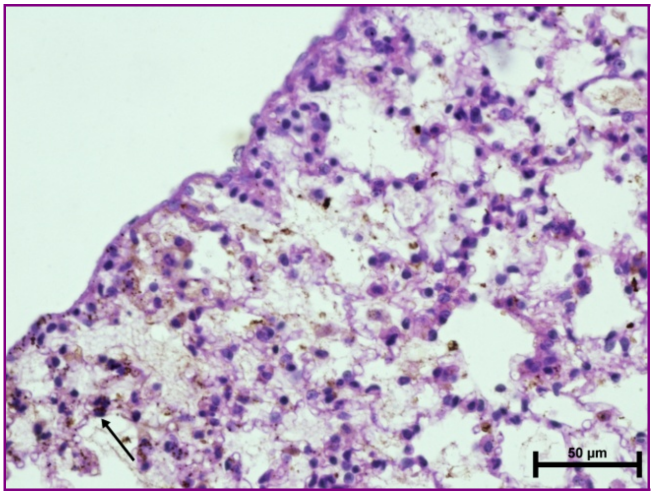

Mechanism of action

Fig. S36 presents the effect of the 4B8M compound on proliferation of CTLL-2 mouse cytotoxic T cells. CTLL-2 cells were cultured in the culture medium for 48h with addition of 15 units of IL-2 and 4B8M peptide at concentrations 100-12.5 µg/ml. Appropriate concentrations of DMSO were added to control cultures. 4B8M moderately inhibited in a dose-dependent manner IL-2 driven growth of CTLL-2 cells.

Fig. S36. IL-2-driven proliferation of CTLL-2 mouse cell line.

Statistics (4B8M vs DMSO): 100 µg/ml P=0.0001; 75 µg/ml P=0.0010; 50 µg/ml P=0.0047; 25 µg/ml NS (P=0.9994); 12.5 µg/ml NS (P=1.0000) (ANOVA)

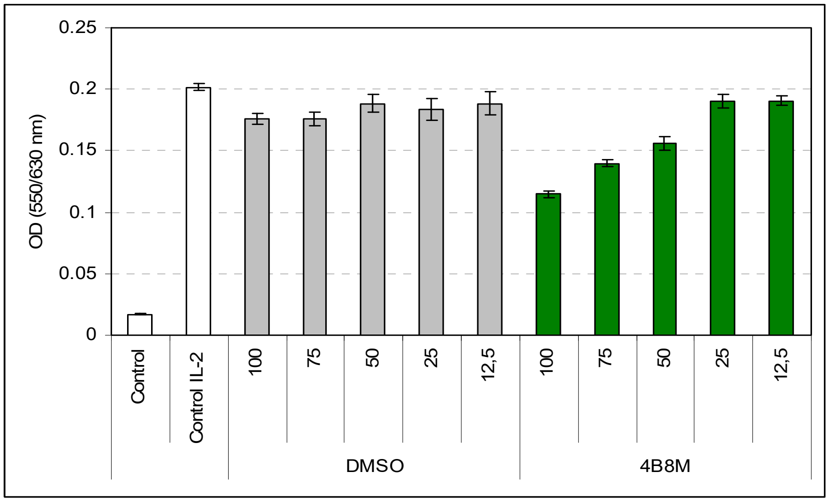

References

Zabrocki J, Zimecki M, Kaszuba A, Kaczmarek K. Cyclic tetrapeptides and therapeutic applications thereof, Patent Number: US 09382292: Official Gazette of the United States Patent and Trademark Office Patents Published: July 5 2016

Hansen MB, Nielsen SE, Berg K: Reexamination and further development of a precise and rapid dye method for measuring cell growth/cell kill. J Immunol Methods, 1989, 119, 203-210

Espevik T, Nissen-Meyer J: A highly sensitive cell line WEHI 164 clone 13, for measuring cytotoxic factor/tumor necrosis factor from human monocytes. J Immunol Methods, 1986, 95, 99-103

Van Snick J, Vink A, Uyttenhove C, Coulie PG, Rubira MR, Simpson RJ: purification and NH_2-terminal amino acid activity for B-cell lymphokine with growth factor activity for B-cell hybridomas. PNAS, 1986, 83, 9679-9683

Mishell RI, Dutton RW: Immunization of dissociated spleen cell cultures from normal mice. J Exp Med, 1967, 126, 423-442

Lagrange PH, Mackaness GB, Miller TE, Pardon P: Influence of dose and route of antigen injection on the immunological function of T cell. J Exp Med, 1974, 139, 528-542

Noonan FP, Halliday WJ: Studies on contact hypersensitivity and tolerance in vivo and in vitro. I. Basic characteristics of the reactions and confirmation of an immune response in tolerant mice. Int Arch Allergy Appl Immunol, 1978, 56, 523-532

Yamamoto S, Sugahara S, Ikeda K, Shimizu Y: Pharmacological profile of a novel phosphodiesterase 7A and – 4 dual inhibitor, YM-393059, on acute and chronic inflammation models. European J Pharmacol, 2006, 550, 166-172

Katayama S, Shionoya H, Ohtake S: A new method for extraction of extravasated dye in the skin and the influence of fasting stress on passive cutaneous anaphylaxis in guinea pigs and rats. Microbiol Immunol 1978, 22(2), 89-101.

Patrick E, Burkhalter A, Maibach HL: Recent investigations of mechanisms of chemically induced skin irritation in laboratory mice. J Invest Dermatol 1987, 88 (3 suppl) 24s-31s

Bottomley KM, Griffiths RJ, Rising TJ, Steward A: A modified mouse air pouch model for evaluating the effects of compounds on granuloma induced cartilage degradation. Br J Pharmacol, 1988, 93, 627-35.

Alves AF, Vieira GC, Gadelha FAAF, Cavalcante-Silva LHA, Martins MA, Barbosa-Filho JA, Piuvezam MR: Milonine, an alkaloid of Cissampelos sympodialis eichl. (Menispermaceae) inhibits histamine release of activated mast cells. Inflammation 2017, 40, 2118-2128

Sampaio ALF, Rae GA, Henriques MGMO: Role of endothelins on lymphocyte accumulation in allergic pleurisy. J Leukoc Biol, 2000, 67, 189-195

Melgar S, Karlsson A, Michaelsson E: Acute colitis induced by dextran sulfate sodium progresses to chronicity in C57BL/6 but not BALB/c mice: correlation between symptoms and inflammation. Am J Physiol Gastrointestinal Liver Physiol, 2005, 288, G1328-G1338
